# Kidney function and cardiovascular diseases: a large-scale observational and Mendelian randomization study

**DOI:** 10.3389/fimmu.2023.1190938

**Published:** 2023-07-17

**Authors:** Chang Hu, Yiming Li, Yaoyao Qian, Zhenying Wu, Bo Hu, Zhiyong Peng

**Affiliations:** ^1^ Department of Critical Care Medicine, Zhongnan Hospital of Wuhan University, Wuhan, Hubei, China; ^2^ Clinical Research Center of Hubei Critical Care Medicine, Zhongnan Hospital of Wuhan University, Wuhan, Hubei, China; ^3^ Department of Critical Care Medicine, Center of Critical Care Nephrology, University of Pittsburgh School of Medicine, Pittsburgh, PA, United States

**Keywords:** Mendelian randomization, chronic kidney disease, estimated glomerular filtration rate, cardiovascular diseases, kidney function

## Abstract

**Background:**

Prior observational studies have found an association between kidney function and cardiovascular diseases (CVDs). However, these studies did not investigate causality. Therefore, the aim of this study is to examine the causal relationship between kidney function and CVDs.

**Methods:**

We utilized data from the eICU Collaborative Research Database (eICU-CRD) from the years 2014-2015 to evaluate the observational association between renal failure (RF) and CVDs. To investigate the causal effects of kidney function (estimated glomerular filtration rate [eGFR] and chronic kidney disease [CKD]) and CVDs (including atrial fibrillation [AF], coronary artery disease [CAD], heart failure [HF], any stroke [AS], and any ischemic stroke [AIS]), we conducted a two-sample bidirectional Mendelian randomization (MR) analysis.

**Results:**

In the observational analysis, a total of 157,883 patients were included. After adjusting for potential confounding factors, there was no significant association between baseline RF and an increased risk of developing CVDs during hospitalization [adjusted odds ratio (OR): 1.056, 95% confidence interval (CI): 0.993 to 1.123, *P* = 0.083]. Conversely, baseline CVDs was significantly associated with an increased risk of developing RF during hospitalization (adjusted OR: 1.189, 95% CI: 1.139 to 1.240, *P* < 0.001). In the MR analysis, genetically predicted AF was associated with an increased risk of CKD (OR: 1.050, 95% CI: 1.016 to 1.085, *P* = 0.004). HF was correlated with lower eGFR (β: -0.056, 95% CI: -0.090 to -0.022, *P* = 0.001). A genetic susceptibility for AS and AIS was linked to lower eGFR (β: -0.057, 95% CI: -0.079 to -0.036, *P* < 0.001; β: -0.029, 95% CI: -0.050 to -0.009, *P* = 0.005; respectively) and a higher risk of CKD (OR: 1.332, 95% CI: 1.162 to 1.528, *P* < 0.001; OR: 1.197, 95% CI: 1.023 to 1.400, *P* = 0.025; respectively). Regarding the reverse direction analysis, there was insufficient evidence to prove the causal effects of kidney function on CVDs. Outcomes remained consistent in sensitivity analyses.

**Conclusion:**

Our study provides evidence for causal effects of CVDs on kidney function. However, the evidence to support the causal effects of kidney function on CVDs is currently insufficient. Further mechanistic studies are required to determine the causality.

## Background

Kidney dysfunction, especially chronic kidney disease (CKD), is a widespread global condition and remains the leading cause of disability and rising health care costs (1). A recent study estimated that there were nearly 700 million global cases of CKD resulting in 35.8 million disability-adjusted life-years in 2017 (2). Consequently, CKD presents significant health and economic burdens due to population growth and aging, and therefore, its prevention is a priority in global public health.

Cardiovascular disease (CVD) is also a growing global issue, affecting approximately 523 million cases in 2019, with marked growth associated with the aging population (3). Previous studies have reported that CVDs and CKD are highly prevalent in elderly individuals and share common predisposing risk factors, such as smoking and hypertension (4, 5). In addition, numerous observational studies have demonstrated that CVDs and CKD are risk factors for each other (6–9). In summary, CKD is linked to an increased risk of CVDs, and conversely, CVDs are associated with an increased risk of CKD, suggesting a bidirectional relationship between these two diseases. However, it remains challenging to determine the existence of a causal link due to overlapping risk factors. Therefore, it is essential to analyze the causal link between CKD and CVDs to gain a better comprehension of the disease’s etiology, as well as to develop effective preventive approaches.

Mendelian randomization (MR) is a valuable method for identifying causal effects between modifiable risk factors and health outcomes from observational data (10). MR utilizes genetic variation [e.g., single nucleotide polymorphism (SNP)]] associated with exposure to investigate their possible causal effect with outcome while reducing bias from confounding (11). This promising technique has been successfully used to investigate the causal effects between lung function and CVDs (12), kidney function and obstructive lung disease (13), and obesity and cardiovascular outcomes (14). However, there is currently a lack of evidence regarding the causal effect between kidney function and CVDs.

In this study, we first examined the observational association between kidney function and CVDs in a large-scale, multicenter cohort. We then conducted a bidirectional MR analysis between kidney function and CVDs to investigate the causal effect between kidney function and CVDs using genome-wide association study (GWAS) data. We hypothesized that there may be a directional causal relationship between these two diseases.

## Methods

### Study design

**Figure 1:**
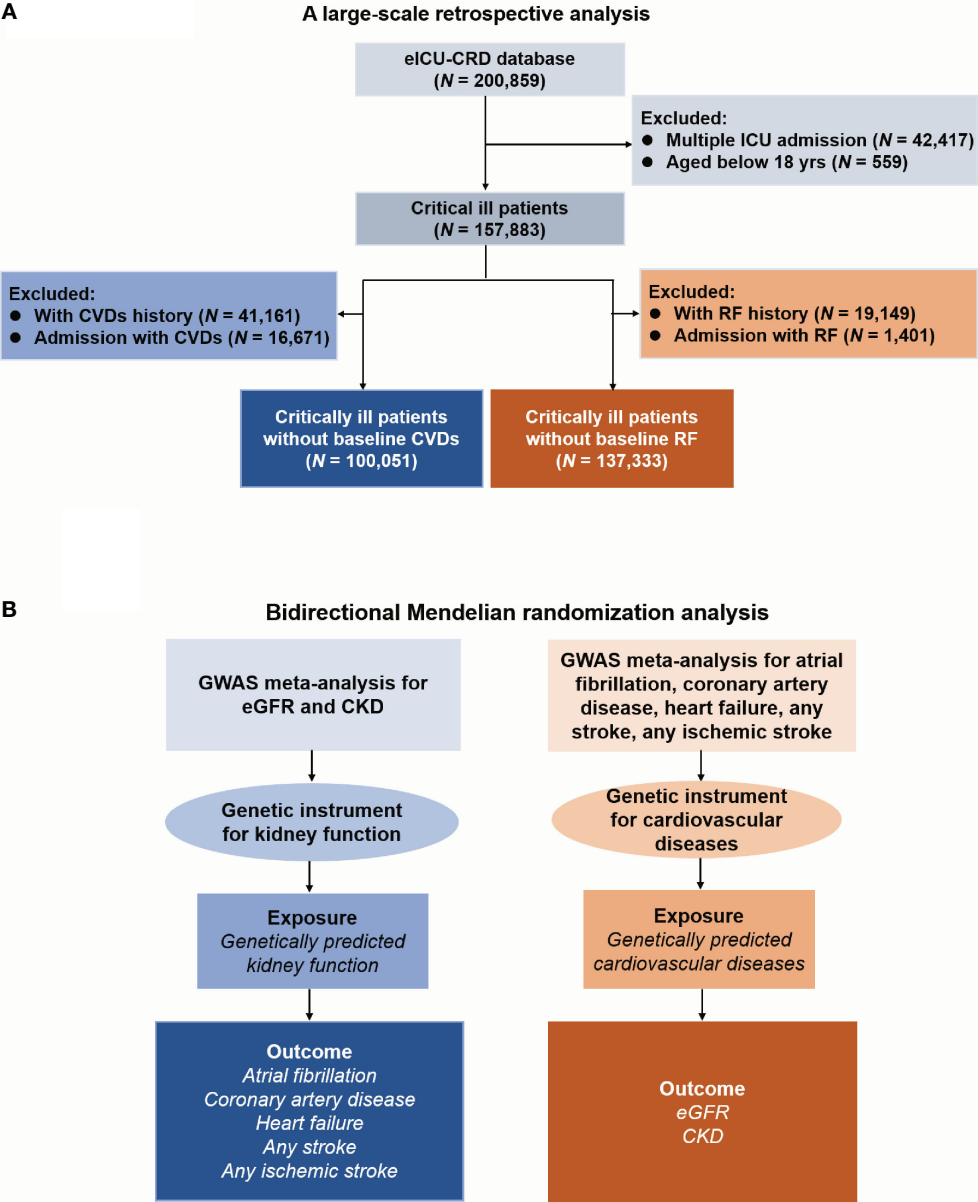
Flow Diagram of this study. eICU-CRD, eICU Collaborative Research Database; ICU, intensive care unit; CVD, cardiovascular disease; RF, renal failure; GWAS, genome-wide association study; eGFR, estimated glomerular filtration rate; CKD, chronic kidney disease. **(A)** Flow chart of the respective analysis; **(B)** Flow chart of the Mendelian randomization analysis.

The study design is presented in **Figure 1**. Firstly, we conducted a retrospective analysis to explore the association between renal failure (RF) and CVDs. Secondly, we used bidirectional MR analysis to investigate the causal effect between kidney function and CVDs.

The necessity for obtaining informed consent was waived because the data used for this retrospective analysis and MR analysis were anonymous and readily available to the public. Additionally, this study was conducted in accordance with the 1964 Helsinki Declaration as well as its subsequent amendments (15). Furthermore, the study was reported in line with the Reporting of Studies Conducted Using Observational Routinely Collected Health Data reporting (RECORD) guideline (16) and Strengthening the Reporting of Observational Studies in Epidemiology Using Mendelian Randomization (STROBE-MR) guideline (17).

### Observational analysis: data source

The eICU Collaborative Research Database (eICU-CRD) is a multi-center intensive care unit (ICU) database developed by Philips Healthcare, comprising over 200,000 ICU admissions from 208 different ICUs across the United States during 2014-2015. Author Chang Hu was granted access to the eICU-CRD (Certification ID: 47460147) after successfully completing a recognized course in protecting human research participants and signing a data use agreement. All The patient data in the eICU-CRD were de-identified and compliant with the Health Insurance Portability and Accountability Act (HIPAA).

### Observational analysis: study population and data extraction

All patients documented in the eICU-CRD were included in this study, except for those with multiple ICU admissions (only the first admission was considered for analysis) and those under 18 years of age. Multiple ICU admissions refer to situations where a patient is admitted to the ICU more than once during a particular hospital stay. For example, a patient may be admitted to the ICU, discharged to a regular ward, and then readmitted to the ICU.

The study extracted the following parameters from the eICU-CRD: age, gender, height, weight, ethnicity, hospital discharge year, ICU type, Acute Physiology and Chronic Health Evaluation (APACHE)-IV score, baseline CVDs, baseline RF, new-onset CVDs during hospitalization and new-onset RF during hospitalization. Among them, CVDs and RF were defined using the International Classification of Diseases, 9th edition (ICD-9) codes (**Supplementary Table S1**). All patient data was acquired from the eICU-CRD using PostgreSQL.

### Observational analysis: association between kidney dysfunction and CVDs

In this analysis, we explored the bidirectional associations of RF with CVDs. Firstly, we examined the association between baseline RF and the risk of new-onset CVDs during hospitalization by excluding patients with baseline CVDs. Second, the association of baseline CVDs with the risk of new-onset RF were excluding patients with baseline RF.

Univariate and multivariate logistic regression analyses were used to investigate the observational association between RF and CVDs. Model 1 was adjusted for age, gender, height, weight, ethnicity, hospital discharge year, and ICU type. Model 2 was adjusted for the same confounders as model 1, plus hospital discharge year and ICU type. Model 3 was adjusted for the confounders included in model 2, as well as the APACHE-IV score.

### MR analysis: data source

The study obtained summary-level GWAS data that are linked to estimated glomerular filtration rate (eGFR), which is based on serum creatinine, from the most large and widely accessible publicly available GWAS meta-analysis (18). The meta-analysis encompassed participants of 1,508,659 European-ancestry. The GWAS information utilized in the study were gathered from the CKDGen (19), MVP (20), Pan-UK Biobank, PAGE (21) and SUMMIT (22) consortia. GWAS data connected to CKD were sourced from the CKDGen Consortium that included 23 cohorts of European ancestry (41,395 cases and 439,303 controls) (19). CKD was defined as having an eGFR of < 60 mL/min/1.73 m^2^.

The GWAS data related to CVDs were extracted from four large-scale meta-analyses. In particular, the GWAS data for atrial fibrillation (AF) consisting of 60,620 cases and 970,216 controls were obtained (23). The AF cases were defined as patients having the billing code ICD-9 to be 427.3 or billing code ICD-10 to be I48, while the controls were individuals without AF. The summary-level GWAS data for coronary artery disease (CAD) were acquired from the Coronary Artery Disease Genome-Wide Replication and Meta-analysis combined with the Coronary Artery Disease Genetics (CARDIoGRAMplusC4D) consortium, which included 60,801 cases and 123,504 controls, with 77% of them being of European ancestry (24). CAD was defined through an all-embracing diagnostic criterion such as myocardial infarction (MI), chronic stable angina, acute coronary syndrome, or coronary stenosis > 50%. The Heart Failure Molecular Epidemiology for Therapeutic Targets (HERMES) consortium contributed to the summary-level GWAS data of HF containing 47,309 cases and 930,014 controls (25). Cases included participants who had clinical diagnosis of heart failure (HF) independently of any criteria based on LV ejection fraction, whereas controls were individuals without HF. The summary-level GWAS data for stroke and ischemic stroke were furnished by METASTROKE, containing 40,585 cases and 406,111 controls, and 34,217 cases and 406,111 controls, respectively (26). In the study, stroke was defined according to the guidelines by the World Health Organization (WHO), that is, the sudden manifestation of signs indicating regional (or global) disturbance of cerebral function, lasting for over 24 hours or leading to death, with no apparent cause other than vascular origin. Strokes were defined to be either ischemic or intracerebral hemorrhage depending on clinical and imaging criteria.

### MR analysis: selection of genetic instrument

We selected the genetic instruments for this study based on published criteria (27). The selection criteria were as follows: 1) SNPs significantly associated with the exposures (*P* < 5 × 10^−8^); 2) Linkage disequilibrium (LD) r^2^ < 0.001 and clump window < 10,000 kb. Furthermore, we excluded the genetic instruments that correspond to the exposures associated with the outcomes (*P* < 5 × 10^−8^), and SNPs with *F*-statistics < 10. We then utilized *F*-statistics to evaluate the strength of the instruments, using the following formula: *F* = *R* ^2^(*N*-*K*-1)/[*K*(1-*R* ^2^)]. In this formula, *R* ^2^ refers to the cumulative explained variance of the selected SNPs during exposure, *K* refers to the number of SNPs in this study, and *N* refers to the number of individuals in the selected GWAS. SNPs with *F*-statistics < 10 were deemed weak instruments. Lastly, exposures with more than two SNPs were selected for further MR analysis. The final selected SNPs are documented in **Supplementary Tables S2**-**S8**.

### MR analysis: primary and secondary analysis

We employed five distinct MR methodologies, namely: random-effects inverse-variance weighted (IVW), MR Egger, weighted median, simple mode, and weighted mode, to establish a statistically significant causal association between kidney function and CVDs, with a *P* value below 0.05. IVW is the dominant method employed in MR analysis, as it combines the Wald ratio estimated for each single-nucleotide polymorphism (SNP) on the outcome and then produces a pooled causal estimate (28). Therefore, in this study, IVW was selected as the principal methodology for identifying preliminary associations between kidney function and CVDs. In addition, four supplementary MR analyses were conducted in conjunction with IVW, as these methodologies could provide more robust estimates across a wider array of scenarios.

### MR analysis: sensitivity analyses

The subsequent techniques were employed to conduct sensitivity analysis. Initially, the Cochran’s Q test was utilized to identify heterogeneity in estimated values based on IVW of SNPs (existence of heterogeneity was inferred at *P* < 0.05). Subsequently, we adopted the MR-Egger intercept method to evaluate whether there was any evidence of horizontal pleiotropy among selected SNPs (existence of horizontal pleiotropy was inferred at *P* < 0.05). Thirdly, we performed the MR Pleiotropy RESidual Sum and Outlier (MR-PRESSO) approach to pinpoint and exclude any outliers, and generate estimates accordingly. Fourthly, we used leave-one-out analysis to examine if any individual SNP had a noticeable effect on the outcome. Finally, we utilized funnel plot to directly detect the presence of pleiotropy.

### Statistical analyses

The normality distribution for continuous variables were accessed using the Kolmogorov–Smirnov test. Normally distributed variables were reported as means ± standard deviation (SD), skewed distributed variables as median and interquartile range (IQR), and categorical variables as numbers and percentages. Student t-test or Mann–Whitney U test were used to compare continuous variables among groups, and Pearson’s chi-squared test or Fisher’s exact test were used for categorical variables, as appropriate.

The association was expressed as odds ratios (OR) and 95% confidence intervals (CI). All statistical assessments were conducted through the implementation of the TwoSampleMR (version 0.4.25) and MR-PRESSO (version 1.0) packages in R (version 4.1.2^®^ Project for Statistical Computing, Vienna, Austria) and SPSS software (version 24.0, Chicago, IL, USA). A two-tailed *P* value of less than 0.05 was considered statistically significant.

## Results

### Observational analysis: characteristics of the cohort

**Figure 1A** presents the flowchart of the observational analysis. Out of the 200,859 patients screened, 42,417 with multiple ICU admissions and 559 non-adult patients were excluded, leaving a total of 157,883 patients enrolled in this study. Of whom, 100,051 patients without baseline CVDs, while 137,333 patients without baseline RF. **Table 1** shows the baseline characteristics of the cohort. Among patients without baseline CVDs, those who developed CVDs during hospitalization tended to be older [70(59-80) vs. 60(47-72) years, *P* < 0.001], had a higher weight [81(67-99) vs. 79(65-95) kg, *P* < 0.001], and had a higher APACHE-IV score [61(45-85) vs. 47(34-64), *P* < 0.001] compared to those who did not develop CVDs during hospitalization. Similarly, among patients without baseline RF, those who developed RF during hospitalization were also older [67(56-78) vs. 64(51-75), *P* < 0.001], had a higher weight [82(68-100) vs. 80(66-96), *P* < 0.001], and tend to have a higher APACHE-IV score [65(49-86) vs. 47(34-62), *P* < 0.001] compared to those who did not develop RF during hospitalization.

**Table 1:** Baseline characteristics of cohort.

	Patients without baseline CVDs (N = 100,051)			Patients without baseline RF (N = 137,333)		
	Incident CVDs (N = 15,256)	No incident CVDs (N = 84,795)	P value	Incident RF (N = 13,627)	No incident RF (N = 123,706)	P value
**Age, median (IQR), yrs**	70(59-80)	60(47-72)	<0.001	67(56-78)	64(51-75)	<0.001
**Gender ^a^, n (%)**			<0.001			<0.001
Male	8407(55.1)	44528(52.6)		7533(55.3)	65973(53.4)	
Female	6843(44.9)	40205(47.4)		6092(44.7)	57658(46.6)	
**Height, median (IQR), cm**	170(163-178)	170(163-178)	0.043	170(163-178)	170(163-178)	0.348
**Weight, median (IQR), kg**	81(67-99)	79(65-95)	<0.001	82(68-100)	80(66-96)	<0.001
**Ethnicity, n (%)**			<0.001			<0.001
Caucasian	12241(80.2)	64874(76.5)		10566(77.5)	96127(77.7)	
African American	1221(8.0)	8965(10.6)		1570(11.5)	12374(10.0)	
Hispanic	695(4.6)	3283(3.9)		651(4.8)	4518(3.7)	
Asian	251(1.6)	1488(1.8)		174(1.3)	2119(1.7)	
Other/Unknown	848(5.6)	6185(7.3)		666(4.9)	8568(6.9)	
**Hospital discharge year, n (%)**			<0.001			0.321
2014	7652(50.2)	40318(47.5)		6585(48.3)	59225(47.9)	
2015	7604(49.8)	44477(52.5)		7042(51.7)	64481(52.1)	
**ICU type, n (%)**			<0.001			<0.001
MS-ICU	10795(70.8)	60343(71.2)		9742(71.5)	87054(70.4)	
Med-ICU	1156(7.6)	7671(9.0)		1341(9.8)	9748(7.9)	
Surg-ICU	3305(21.7)	16781(19.8)		2544(18.7)	26904(21.7)	
**APACHE-IV, median (IQR)**	61(45-85)	47(34-64)	<0.001	65(49-86)	47(34-62)	<0.001
**Baseline CVDs, n (%)**	0	0		5232(38.4)	41262(33.4)	<0.001
**Baseline RF, n (%)**	1959(12.8)	7253(8.6)	<0.001	0	0	

^a^68 patients with missing information on gender in the group of patients without baseline CVDs and 77 patients with missing information on gender in the group of patients without baseline RF.

CVD, cardiovascular disease; RF, renal failure; ICU, intensive care unit; APACHE, Acute Physiology and Chronic Health Evaluation; IQR, interquartile range.

### Observational analysis: association between kidney dysfunction and CVDs

**Figure 2:**
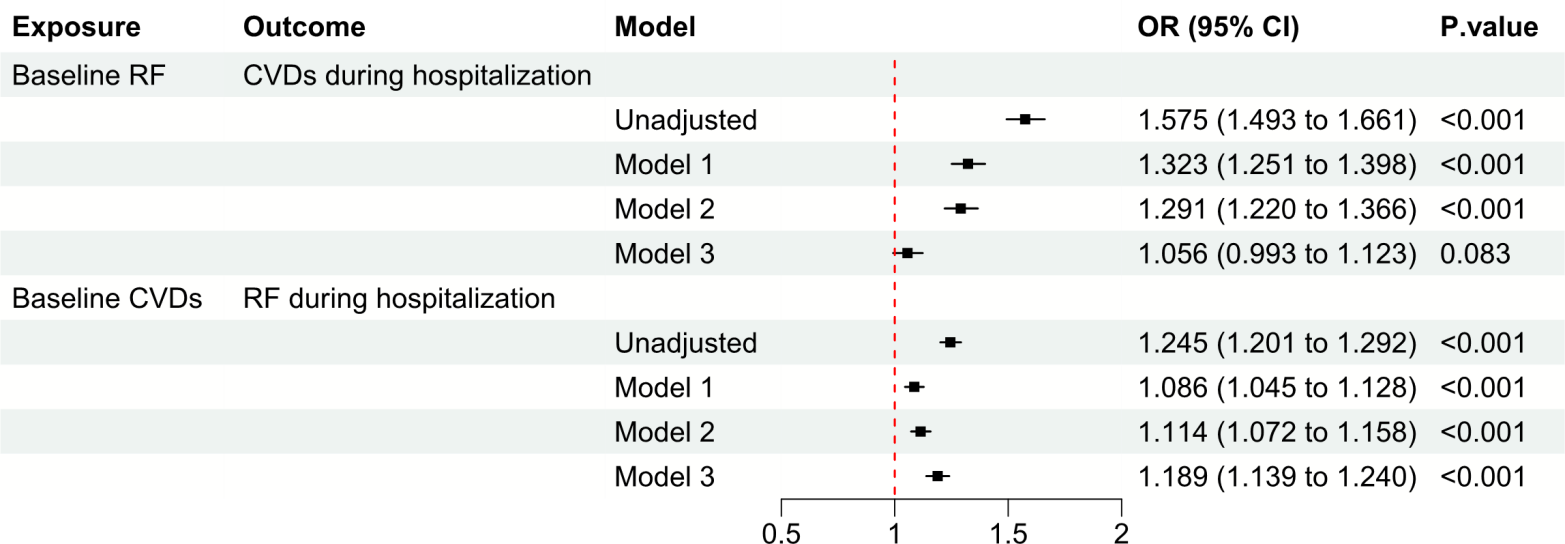
Observational analysis explores the association between CVDs and RF. CVD, cardiovascular disease; RF, renal failure; OR, odds ratio; CI, confidence interval.

Among patients without baseline CVDs, those with baseline RF had a higher risk of developing CVDs during hospitalization compared to those without baseline RF (unadjusted OR: 1.575, OR: 1.493 to 1.661, *P* < 0.001). However, after adjusting for covariates such as age, gender, height, weight, ethnicity, hospital discharge year, ICU type, and APACHE-IV score, there was no significant association between baseline RF and the risk of developing CVDs during hospitalization (adjusted OR: 1.056, 95% CI: 0.993 to 1.123, *P* = 0.083) (**Figure 2**). Conversely, among patients without baseline RF, those with baseline CVDs had higher risk of developing RF during hospitalization compared to those without baseline CVDs (unadjusted OR: 1.245, OR: 1.201 to 1.292, *P* < 0.001). After adjustment, baseline CVDs was found to be significantly associated with an increased risk of developing RF during hospitalization (adjusted OR: 1.189, 95% CI: 1.139 to 1.240, *P* < 0.001) (**Figure 2**).

### MR analysis: characteristics of the genetic instruments

**Figure 1B** shows the flowchart of the MR analysis. **Table 2** provides comprehensive data regarding the GWAS data sources, years, sample size, and ancestry in the analysis. The GWAS investigations for seven distinct phenotypes were published between 2015 and 2022, and the population sizes ranged from 184,305 to 1,508,659. Except for CARDIoGRAMplusC4D (which reported 23% of non-European ancestry), the GWAS data were carried out among populations of European descent. Following the selection process, we incorporated the use of 404 SNPs for eGFR, 20 for CKD, 92 for AF, 19 for CAD, 9 for HF, 7 for AS, and 9 for AIS, respectively, as our instrumental variables. The instrumental variable for each phenotype is presented in **Supplementary Tables S2**-**S9**.

**Table 2:** GWAS data for kidney function and cardiovascular diseases.

Phenotype	Data source	Year	Sample size (cases/controls)	Ancestry
Kidney function				
eGFR	CKDGen, Pan-UK Biobank, MVP, PAGE, SUMMIT	2022	1,508,659	European
CKD	CKDGen	2019	480,698 (41,395/439,303)	European
Cardiovascular diseases				
Atrial fibrillation	HUNT, deCODE, the MGI, DiscovEHR, UK Biobank, AFGen	2018	1,030,836 (60,620/970,216)	European
Coronary artery disease	CARDIoGRAMplusC4D	2015	184,305 (60,801/123,504)	77% European
Heart failure	HERMES	2020	977,323 (47,309/930,014)	European
Any stroke	METASTROKE	2018	446,696 (40,585/406,111)	European
Any ischemic stroke	METASTROKE	2018	440,328 (34,217/406,111)	European

eGFR, estimated glomerular filtration rate; CKD, chronic kidney disease.

### MR analysis: kidney function as exposure, CVDs as outcome

**Figure 3:**
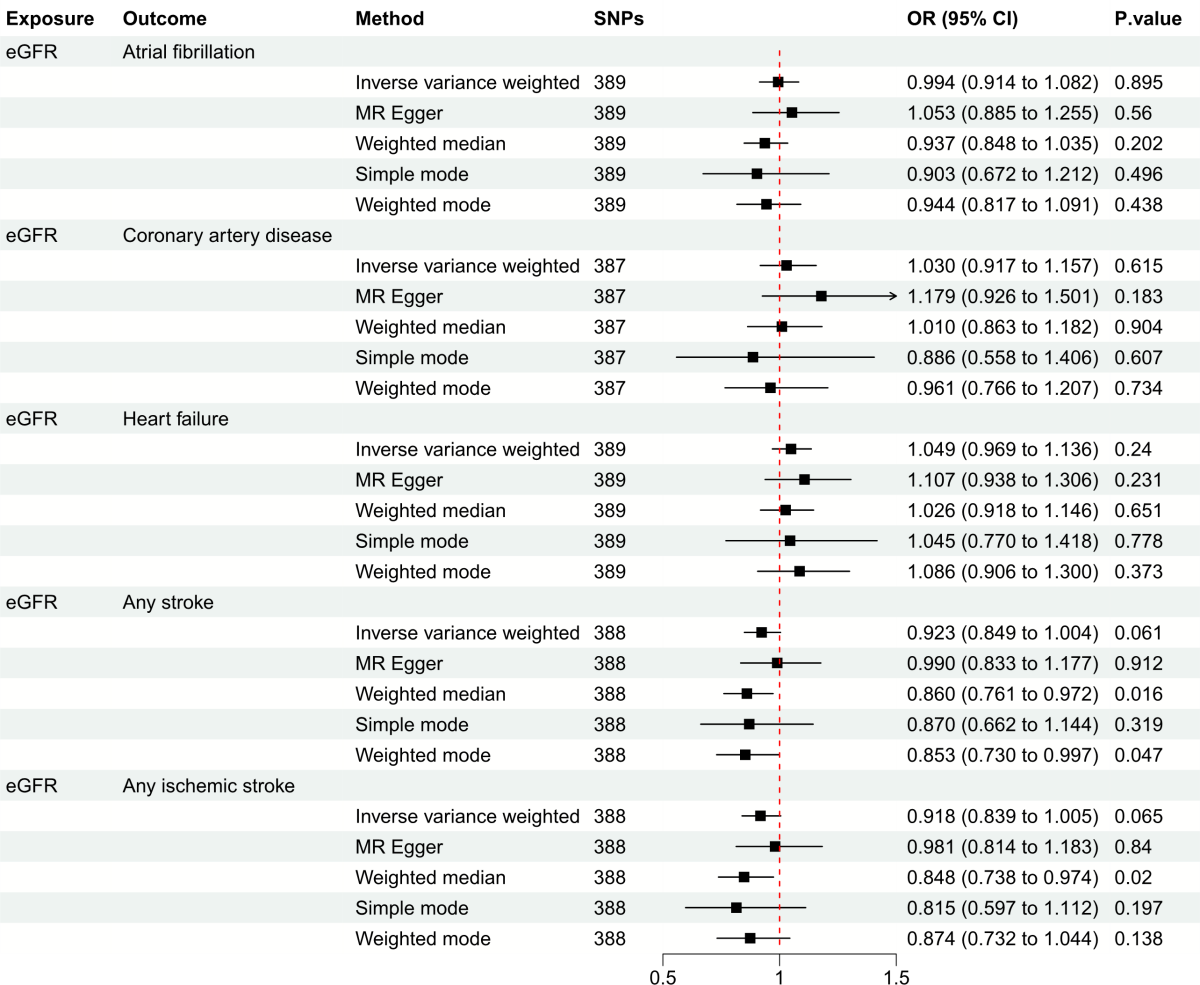
Mendelian randomization estimates of eGFR on the risk for CVDs. SNPs, single-nucleotide polymorphisms; eGFR, estimated glomerular filtration rate; CVD, cardiovascular disease; OR, odds ratio; CI, confidence interval.

**Figure 4:**
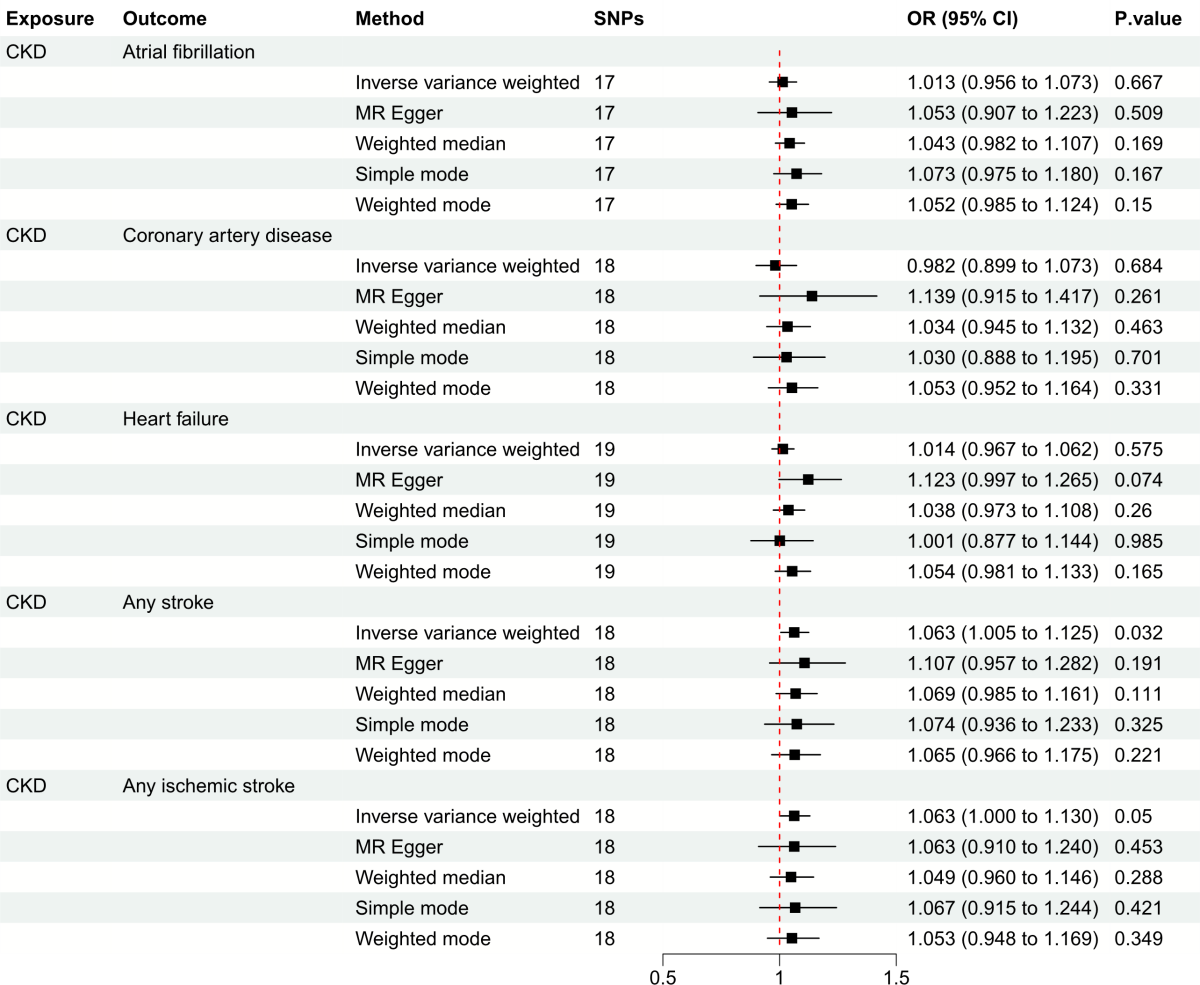
Mendelian randomization estimates of CKD on the risk for CVDs. SNPs, single-nucleotide polymorphisms; CKD, chronic kidney disease; CVD, cardiovascular disease; OR, odds ratio; CI, confidence interval.

**Figure 3** depicts the causal effect of eGFR on the risk for CVDs. The random-effect IVW method produced non-significant estimates for eGFR in relation to AF (OR: 0.994, 95%CI: 0.914 to 1.082, *P* = 0.895), CAD (OR: 1.030, 95%CI: 0.917 to 1.157, *P* = 0.615), HF (OR: 1.049, 95%CI: 0.969 to 1.136, *P* = 0.240), AS (OR: 0.923, 95%CI: 0.849 to 1.004, *P* = 0.061) and AIS (OR: 0.918, 95%CI: 0.839 to 1.005, *P* = 0.065) (**Supplementary Figure S1**). Furthermore, **Figure 4** illustrates the associations between CKD and the risk for CVDs. The genetically predicted CKD was significantly related with a higher risk of AS (OR: 1.063, 95%CI: 1.005 to 1.125, *P* = 0.032). However, non-significant estimates were found for CKD in relation to AF (OR: 1.013, 95%CI: 0.956 to 1.073, *P* = 0.667), CAD (OR: 0.982, 95%CI: 0.899 to 1.073, *P* = 0.684), HF (OR: 1.014, 95%CI: 0.967 to 1.062, *P* = 0.575) and AIS (OR: 1.063, 95%CI: 1.000 to 1.130, *P* = 0.050) (**Supplementary Figure S2**).

### MR analysis: CVDs as exposure, kidney function as outcome

**Table 3** lists the MR estimates of the CVDs’ impact on kidney function. For AF, the genetically predicted AF exhibited a significant association with an increased risk of CKD (OR: 1.050, 95%CI: 1.016 to 1.085, *P* = 0.004); however, it revealed a non-significant association with eGFR change (β: -0.007, 95%CI: -0.014 to 0.00, *P* = 0.050) (**Supplementary Figure S3**). For CAD, no significant correlations were found between CAD and eGFR (β: -0.001, 95%CI: -0.023 to 0.021, *P* = 0.917), or CKD (OR: 0.994, 95%CI: 0.900 to 1.097, *P* = 0.899) (**Supplementary Figure S4**). For HF, genetically predicted HF was significantly associated with a lower eGFR (β: -0.056, 95%CI: -0.090 to -0.022, *P* = 0.001); nonetheless, it exhibited no significant association with the risk of CKD (OR: 1.115, 95%CI: 0.871 to 1.427, *P* = 0.389) (**Supplementary Figure S5**). For AS and AIS, a genetic predisposition for both AS and AIS showed a significant association with lower eGFR (β: -0.057, 95%CI: -0.079 to -0.036, *P* < 0.001; β: -0.029, 95%CI: -0.050 to -0.009, *P* = 0.005; respectively) and an increased risk of CKD (OR: 1.332, 95%CI: 1.162 to 1.528, *P* < 0.001; OR: 1.197, 95%CI: 1.023 to 1.400, *P* = 0.025; respectively) in the MR analysis (**Supplementary Figures S6**, **S7**).

**Table 3:** Mendelian randomization estimates from CVDs on genetically predicted kidney function.

Exposure	Outcome	Method	No. SNPs	β/OR	95% CI	*P* value
**AF**	eGFR	IVW	83	-0.007	-0.014 to 0.000	0.050
		MR Egger	83	-0.006	-0.020 to 0.007	0.375
		Weighted median	83	-0.010	-0.017 to -0.003	0.007
		Simple mode	83	0.004	-0.017 to 0.024	0.724
		Weighted mode	83	-0.004	-0.011 to 0.003	0.306
	CKD	IVW	90	1.050	1.016 to 1.085	0.004
		Weighted median	90	1.033	0.985 to 1.084	0.180
		MR Egger	90	1.016	0.953 to 1.083	0.629
		Simple mode	90	1.044	0.949 to 1.149	0.380
		Weighted mode	90	1.032	0.976 to 1.092	0.266
**CAD**	eGFR	IVW	13	-0.001	-0.023 to 0.021	0.917
		Weighted median	13	-0.003	-0.017 to 0.011	0.635
		MR Egger	13	-0.052	-0.101 to -0.004	0.058
		Simple mode	13	0.007	-0.021 to 0.035	0.636
		Weighted mode	13	-0.013	-0.023 to -0.003	0.027
	CKD	IVW	18	0.994	0.900 to 1.097	0.899
		Weighted median	18	1.024	0.942 to 1.113	0.573
		MR Egger	18	1.063	0.835 to 1.353	0.627
		Simple mode	18	0.886	0.730 to 1.074	0.235
		Weighted mode	18	1.027	0.938 to 1.124	0.572
**HF**	eGFR	IVW	6	-0.056	-0.090 to -0.022	0.001
		Weighted median	6	-0.050	-0.075 to -0.026	<0.001
		MR Egger	6	-0.053	-0.203 to 0.096	0.523
		Simple mode	6	-0.073	-0.122 to -0.024	0.033
		Weighted mode	6	-0.048	-0.079 to -0.017	0.030
	CKD	IVW	8	1.115	0.871 to 1.427	0.389
		Weighted median	8	1.218	1.011 to 1.469	0.038
		MR Egger	8	1.102	0.314 to 3.866	0.884
		Simple mode	8	1.419	1.069 to 1.883	0.046
		Weighted mode	8	1.315	1.033 to 1.675	0.062
**AS**	eGFR	IVW	6	-0.057	-0.079 to -0.036	<0.001
		Weighted median	6	-0.050	-0.074 to -0.026	<0.001
		MR Egger	6	0.003	-0.133 to 0.138	0.973
		Simple mode	6	-0.038	-0.076 to 0.001	0.053
		Weighted mode	6	-0.041	-0.070 to -0.013	0.021
	CKD	IVW	7	1.332	1.162 to 1.528	<0.001
		MR Egger	7	1.722	0.631 to 4.699	0.337
		Weighted median	7	1.291	1.099 to 1.516	0.002
		Simple mode	7	1.209	0.955 to 1.529	0.165
		Weighted mode	7	1.229	0.969 to 1.559	0.141
**AIS**	eGFR	IVW	6	-0.029	-0.050 to -0.009	0.005
		MR Egger	6	-0.135	--0.230 to -0.040	0.032
		Weighted median	6	-0.029	-0.049 to -0.009	0.004
		Simple mode	6	-0.026	-0.052 to 0.000	0.050
		Weighted mode	6	-0.028	-0.052 to -0.005	0.008
	CKD	IVW	9	1.197	1.023 to 1.400	0.025
		Weighted median	9	1.198	1.037 to 1.384	0.014
		MR Egger	9	2.023	0.624 to 6.555	0.279
		Simple mode	9	1.191	0.943 to 1.504	0.180
		Weighted mode	9	1.204	0.954 to 1.521	0.157

CVD, cardiovascular disease; AF, atrial fibrillation; CAD, coronary artery disease; HF, heart failure; AS, any stroke; AIS, any ischemic stroke; eGFR, estimated glomerular filtration rate; CKD, chronic kidney disease; IVW, inverse-variance weighted.

### MR analysis: sensitivity analyses

We conducted a series of sensitivity analyses, including the Cochran’s Q test, MR-Egger intercept test, MR-PRESSO global test, leave-one-out analysis, and funnel plot, to evaluate the durability of our findings. The MR-Egger intercept tests exhibited *P* values greater than 0.05, suggesting the lack of horizontal pleiotropy in our study (**Supplementary Tables S9**, **S10**). Although some Cochran’s Q test results indicated heterogeneity, the MR estimates in our study were not invalidated because of the IVW method, which helped to stabilize the pooled heterogeneity (**Supplementary Tables S9**, **S10**). In addition, we utilized the MR-PRESSO approach to eliminate exceptional SNPs and identified stable outcomes, excluding CKD on AS and AIS (**Supplementary Tables S9**, **S10**). Furthermore, the leave-one-out analysis confirmed the robustness of the results when individual SNP was excluded (**Supplementary Figures S8**-**S14**). Finally, careful examination of the funnel plots did not reveal significant signs of asymmetry in the MR analysis (**Supplementary Figures S15**-**S21**).

## Discussion

This study first demonstrated that baseline CVDs were associated with an increased risk of developing RF during hospitalization, while non-significant association was found between baseline RF and the risk of developing CVDs during hospitalization in the eICU-CRD. Further bidirectional MR analysis revealed a significantly and causally link between CVDs (excluding CAD) and decline of kidney function. However, there is limited evidence to support a causal effect of kidney function on CVDs in reverse.

Presently, numerous observational studies have reported the correlation between kidney function and the risk of CVDs (29–31). For example, Guo and colleagues reported in a prospective cohort of 37,691 participants that a decline in eGFR was significantly associated with an increased risk of CVDs after adjusting for potential covariates (29). Another prospective cohort study of 7,098 individuals also indicated that kidney dysfunction, such as lower eGFR and higher albuminuria, was linked with incident CVDs (30). Furthermore, the Acute Decompensated Heart Failure National Registry (ADHERE) had disclosed that roughly 30% of HF patients experience acute or chronic renal insufficiency (31). In our observational analysis of a large cohort, we investigated the association between CVDs and RF and found that baseline CVDs were associated with an increased risk of developing RF. However, we did not find a significant association between baseline RF and the development of CVDs. Nonetheless, the observational findings might be influenced by unnoticed confounding effects and reverse causation, which undermine the robustness of the results. Hence, it is necessary to ascertain whether a causal effect exists in the bidirectional association between the two conditions.

In the present study, we further utilized bidirectional MR analysis to determine the causal effects between kidney function and CVDs. MR is a sophisticated approach that can surmount unobserved confounding effects and reverse causation (11). The MR analysis involves three primary stages. Firstly, it is essential to establish a robust association between the genetic instrument and the exposure. To achieve this, we selected SNPs that were significantly (*P* < 5 × 10^-8^) associated with the exposures (either kidney function or CVDs, as appropriate). Secondly, the effect of the genetic instrument should have a direct impact on the exposure. Therefore, we conducted an MR-Egger intercept test and verified the absence of horizontal pleiotropy in our study. Thirdly, the genetic instrument should not be influenced by confounding factors that affect the outcome. In this MR analysis, we found that CVDs substantively increase the risk of kidney dysfunction. However, the causal effect of kidney function on CVDs remains unclear.

Although the precise mechanism linking CVDs with kidney function has not been fully elucidated, several possible rationales have been proposed, which are as follows: 1) Patients with CVDs, particularly those with AF and HF, often experience lower cardiac output, which can increase kidney venous pressure and result in a decline in kidney function (32). 2) As a vital autonomous nervous system, the renin-angiotensin-aldosterone system, plays a crucial role in kidney function. Nonetheless, the overactivation of the renin-angiotensin-aldosterone system caused by the CVDs may stimulate renal fibrosis and the progression to end-stage renal disease (33, 34). 3) As a frequent complication in AF and stroke, thromboembolism may lead to acute or chronic renal infarction, which would impair the kidney function (35). 4) Other potential factors such as inflammatory cytokine release, oxidative stress and endothelial dysfunction due to CVDs, may affect the kidney function (36).

The non-significant causal effects of kidney function on CVDs should be interpreted with caution. The non-significant causal effects of kidney function on cardiovascular diseases (CVDs) should be interpreted with caution. Although we observed a positive outcome that indicated a significant association between genetically predicted CKD and a higher risk of AS using the IVW method, the OR was relatively low (OR: 1.063, 95% CI: 1.005 to 1.125, *P* = 0.032). Moreover, we employed the MR-PRESSO method to account for the effect of outliers and found little evidence of a causal relationship between CKD and AS. To the best of our knowledge, the majority of CKD cases were mild to moderate in severity. Only 18% (7514/41395) of CKD patients had established CKD stages 3-5 in the CKDGen Consortium (19). Thus, a slight change in kidney function was improbable to significantly increase the risk of CVDs. Despite several previous observational studies found that patients with kidney dysfunction have a greater risk for CVDs, there was a lack of potential confounding factors adjustment and causality verification (29, 37, 38). In our observational analysis, we observed that patients with baseline RF had a significantly higher risk of developing CVDs during hospitalization compared to those without baseline RF. However, after adjusting for potential confounding factors such as age, gender, height, weight, ethnicity, hospital discharge year, ICU type, and APACHE-IV score, we found no significant association between baseline RF and the risk of developing CVDs during hospitalization. To further address potential confounding factors and provide a more accurate estimate of the causal relationship between CKD and CVD, we used the MR method in our study. The MR analysis results confirmed our findings from the observational analysis.

Our findings contribute to the comprehension of the causal impacts of CVDs on kidney function, thereby potentially enhancing clinical practices for both researchers and practitioners. For instance, medical professionals can adopt appropriate measures to mitigate the risks of kidney insufficiency among patients with CVDs. Moreover, clinicians may need to reinforce monitoring of kidney function changes in patients with CVDs, who are at increased risk of kidney dysfunction. Additionally, early screening for kidney impairment among patients with CVDs could be beneficial. These strategies have the potential to improve clinical outcomes in patients with CVDs.

However, there are several limitations in this study. First, the participants included in the observational analysis and MR analysis were derived from different sources, and the vast majority of those who participated in the MR analysis were of European ancestry, which may limit the generalizability of our conclusions. Second, despite careful adjustments for various confounding factors in the observational analysis, there may still be a potential for bias from residual and unmeasured confounding in our study. Third, due to limitations in the genetic data utilized in our GWAS, we were unable to conduct stratified analyses based on factors of interest, such as age or gender. Fourth, although our findings highlight the causal relationship between CVDs and kidney function, it relies on a series of pre-existing assumptions, and clinical studies will be needed in the future to confirm causality and explore potential mechanisms.

## Conclusion

Our study provides evidence for causal effects of CVDs on kidney function. However, the evidence to support the causal effects of kidney function on CVDs is currently insufficient. Further mechanistic studies are required to determine the causality.

## Data availability statement

The original contributions presented in the study are included in the article/**Supplementary Material**. Further inquiries can be directed to the corresponding authors.

## Author contributions

CH: Conceptualization, Software, Validation, Writing-original draft. YL: Software, Visualization. YQ: Data curation. ZW: Data curation. BH: Conceptualization, Methodology, Supervision. ZP: Conceptualization, Methodology, Supervision. All authors contributed to the article and approved the submitted version.
