# Supplementary material for: Kidney function and cardiovascular diseases: a large-scale observational and Mendelian randomization study
Source: Front Immunol. 2023 Jul 17;14:1190938. doi: 10.3389/fimmu.2023.1190938 (PMC10390297; doi:10.3389/fimmu.2023.1190938)
Supplement: Supplementary file 1 [file DataSheet_1.docx]

**Kidney Function and** **Cardiovascular Diseases: A Large-Scale** **Observational and Mendelian Randomization Study**

Chang Hu, Yiming Li, Yaoyao Qian, Zhenying Wu, Bo Hu, Zhiyong Peng

**Online Data Supplement**

**Supplementary Tables and Figures cited in main text**

- Table S1. Diagnostic criteria for CVDs and RF in the eICU-CRD.
- Table S2. 404 index SNPs represented genetically predicted eGFR.
- Table S3. 20 index SNPs represented genetically predicted CKD.
- Table S4. 92 index SNPs represented genetically predicted atrial fibrillation.
- Table S5. 19 index SNPs represented genetically predicted coronary artery disease.
- Table S6. 9 index SNPs represented genetically predicted heart failure.
- Table S7. 7 index SNPs represented genetically predicted any stroke.
- Table S8. 9 index SNPs represented genetically predicted any ischemic stroke.
- Table S9. The results of pleiotropy test, Cochrane’s Q and MR-PRESSO for kidney function on CVDs.
- Table S10. The results of pleiotropy test, Cochrane’s Q and MR-PRESSO for CVDs on kidney function.
- Figure S1. Scatter plots of MR tests from eGFR on cardiovascular diseases.
- Figure S2. Scatter plots of MR tests from CKD on cardiovascular diseases.
- Figure S3. Scatter plots of MR tests from atrial fibrillation on kidney function.
- Figure S4. Scatter plots of MR tests from coronary artery disease on kidney function.
- Figure S5. Scatter plots of MR tests from heart failure on kidney function.
- Figure S6. Scatter plots of MR tests from any stroke on kidney function.
- Figure S7. Scatter plots of MR tests from any ischemic stroke on kidney function.
- Figure S8. Leave-one-out plots of MR tests from eGFR on cardiovascular diseases.
- Figure S9. Leave-one-out plots of MR tests from CKD on cardiovascular diseases.
- Figure S10. Leave-one-out plots of MR tests from atrial fibrillation on kidney function.
- Figure S11. Leave-one-out plots of MR tests from coronary artery disease on kidney function.
- Figure S12. Leave-one-out plots of MR tests from heart failure on kidney function.
- Figure S13. Leave-one-out plots of MR tests from any stroke on kidney function.
- Figure S14. Leave-one-out plots of MR tests from any ischemic stroke on kidney function.
- Figure S15. Funnel plots of MR tests from eGFR on cardiovascular diseases.
- Figure S16. Funnel plots of MR tests from CKD on cardiovascular diseases.
- Figure S17. Funnel plots of MR tests from atrial fibrillation on kidney function.
- Figure S18. Funnel plots of MR tests from coronary artery disease on kidney function.
- Figure S19. Funnel plots of MR tests from heart failure on kidney function.
- Figure S20. Funnel plots of MR tests from any stroke on kidney function.
- Figure S21. Funnel plots of MR tests from any ischemic stroke on kidney function.
- Acknowledgments.

**Table S1. Diagnostic criteria for CVDs and RF in the** **eICU-CRD.**

| **Phenotype** | **ICD-9 code** |
| --- | --- |
| **CVDs during hospitalization** |  |
| Atrial fibrillation | 427, 427.1, 427.31, 427.32, 427.41, 427.5, 427.61, 427.69, 427.81, 427.89, 427.9 |
| Congestive heart failure | 428, 428.1, 428.2, 428.21, 428.22, 428.23, 428.3, 428.31, 428.32, 428.33, 428.4, 428.41, 428.42, 428.43, 428.9 |
| Ischemic heart disease | 410.1, 410.11, 410.21, 410.31, 410.41, 410.51, 410.61, 410.7, 410.71, 410.81, 410.9, 411.89, 411.81, 411.1, 411, 413.1, 414, 414.01, 414.02, 414.03, 414.04, 414.06 |
| Stroke | 434.11, 434.9, 434.91 |
| **RF during hospitalization** |  |
| Chronic kidney disease | 585.1, 585.2, 585.3, 585.4, 585.5, 585.6, 585.9 |
| Renal failure | 584.5, 584.9 |

CVD, cardiovascular disease; RF, renal failure; eICU-CRD, eICU Collaborative Research Database; ICD, International Classification of Diseases.

**Table S2. 404 index SNPs represented genetically predicted eGFR.**

| **SNP** | **other_allele** | **effect_allele** | **eaf** | **beta** | **se** | **pval** | **F-statistic** |
| --- | --- | --- | --- | --- | --- | --- | --- |
| rs1694595 | T | G | 0.8073 | 0.008404 | 0.00146 | 8.56E-09 | 10.31196 |
| rs1165227 | T | C | 0.8469 | -0.01144 | 0.001684 | 1.08E-11 | 11.97344 |
| rs499600 | T | G | 0.816 | 0.015243 | 0.001486 | 1.10E-24 | 31.59273 |
| rs4442348 | A | G | 0.5079 | -0.01069 | 0.001154 | 2.01E-20 | 42.88273 |
| rs848307 | A | G | 0.8561 | 0.012155 | 0.001818 | 2.29E-11 | 11.01414 |
| rs2807660 | A | G | 0.574 | -0.00831 | 0.001181 | 1.94E-12 | 24.23149 |
| rs1173643 | A | G | 0.5164 | -0.00673 | 0.001154 | 5.48E-09 | 16.98799 |
| rs953492 | A | G | 0.5423 | 0.012948 | 0.001158 | 4.88E-29 | 62.09587 |
| rs3107148 | T | C | 0.6218 | 0.008381 | 0.001192 | 2.08E-12 | 23.23784 |
| rs754984 | C | G | 0.1018 | 0.020458 | 0.001904 | 6.39E-27 | 21.10609 |
| rs2235537 | T | C | 0.4262 | 0.006929 | 0.001184 | 4.86E-09 | 16.75008 |
| rs7413585 | T | C | 0.4745 | -0.00657 | 0.001169 | 1.91E-08 | 15.75127 |
| rs267738 | T | G | 0.2004 | 0.026182 | 0.001443 | 1.28E-73 | 105.5805 |
| rs760418 | C | G | 0.4891 | 0.009152 | 0.001168 | 4.69E-15 | 30.6796 |
| rs12722725 | T | C | 0.1247 | -0.0303 | 0.001851 | 3.03E-60 | 58.51582 |
| rs11166440 | A | G | 0.3835 | -0.01122 | 0.001187 | 3.14E-21 | 42.29984 |
| rs61830291 | A | C | 0.0934 | 0.019811 | 0.002131 | 1.43E-20 | 14.64115 |
| rs9659443 | G | A | 0.3336 | -0.01422 | 0.001221 | 2.46E-31 | 60.28538 |
| rs706406 | C | A | 0.3883 | 0.012513 | 0.001198 | 1.56E-25 | 51.81838 |
| rs236326 | T | A | 0.2535 | 0.011887 | 0.001324 | 2.68E-19 | 30.52777 |
| rs57445665 | G | A | 0.212 | 0.010338 | 0.001429 | 4.66E-13 | 17.48936 |
| rs17602729 | G | A | 0.1241 | 0.013831 | 0.001879 | 1.83E-13 | 11.77966 |
| rs1166698 | G | A | 0.2252 | 0.012937 | 0.001381 | 7.25E-21 | 30.63902 |
| rs4971059 | G | A | 0.3976 | 0.008744 | 0.001194 | 2.37E-13 | 25.70999 |
| rs1123571 | G | A | 0.4384 | 0.007829 | 0.001292 | 1.36E-09 | 18.0833 |
| rs3850625 | G | A | 0.1169 | 0.025163 | 0.001808 | 4.99E-44 | 39.99049 |
| rs11261022 | C | A | 0.3457 | -0.01173 | 0.001212 | 3.74E-22 | 42.37301 |
| rs1198975 | G | A | 0.3849 | -0.01146 | 0.0012 | 1.39E-21 | 43.12263 |
| rs3795503 | C | T | 0.3306 | 0.012369 | 0.001242 | 2.35E-23 | 43.88211 |
| rs10920308 | C | A | 0.4962 | -0.0077 | 0.001154 | 2.47E-11 | 22.27684 |
| rs2749151 | C | T | 0.2 | 0.009835 | 0.001595 | 7.09E-10 | 12.15845 |
| rs4653921 | C | A | 0.3324 | 0.007898 | 0.001222 | 1.03E-10 | 18.53876 |
| rs10857788 | G | A | 0.6983 | 0.015205 | 0.001254 | 8.08E-34 | 61.91753 |
| rs12727107 | G | A | 0.5237 | 0.006677 | 0.001153 | 7.00E-09 | 16.73034 |
| rs12402954 | G | A | 0.6326 | -0.01044 | 0.001194 | 2.27E-18 | 35.53273 |
| rs4415537 | C | T | 0.7393 | 0.010621 | 0.00133 | 1.41E-15 | 24.57812 |
| rs6744604 | A | G | 0.6 | -0.00866 | 0.001255 | 5.24E-12 | 22.84651 |
| rs1548945 | T | C | 0.5703 | -0.01898 | 0.00118 | 3.52E-58 | 126.7379 |
| rs12614953 | T | C | 0.5008 | 0.012868 | 0.001168 | 3.09E-28 | 60.71133 |
| rs847161 | A | G | 0.768 | -0.01034 | 0.001383 | 7.60E-14 | 19.92228 |
| rs1260326 | T | C | 0.5988 | -0.02672 | 0.001195 | 9.03E-111 | 240.3262 |
| rs187355703 | C | G | 0.0263 | -0.06071 | 0.003894 | 8.78E-55 | 12.445 |
| rs12464085 | A | G | 0.3321 | 0.016223 | 0.001225 | 4.82E-40 | 77.82799 |
| rs77375846 | T | C | 0.1249 | -0.01506 | 0.001873 | 8.98E-16 | 14.13077 |
| rs3770636 | T | G | 0.0836 | 0.029009 | 0.002136 | 5.05E-42 | 28.26965 |
| rs10194077 | T | C | 0.3782 | 0.007268 | 0.001187 | 9.24E-10 | 17.6276 |
| rs12713261 | T | C | 0.3435 | -0.01266 | 0.00123 | 7.35E-25 | 47.81248 |
| rs55944332 | A | G | 0.2191 | -0.01027 | 0.001392 | 1.57E-13 | 18.64244 |
| rs13410232 | A | T | 0.2271 | 0.034489 | 0.001454 | 2.09E-124 | 197.5905 |
| rs4491726 | A | G | 0.3059 | -0.01472 | 0.00125 | 4.95E-32 | 58.9303 |
| rs1446308 | T | C | 0.3474 | 0.009509 | 0.001209 | 3.69E-15 | 28.04865 |
| rs13032786 | C | G | 0.3178 | -0.01374 | 0.001254 | 5.95E-28 | 52.08722 |
| rs2301343 | T | G | 0.2379 | 0.013621 | 0.001356 | 9.76E-24 | 36.58135 |
| rs34602370 | T | C | 0.3131 | 0.009357 | 0.001259 | 1.08E-13 | 23.75238 |
| rs2311597 | A | G | 0.4317 | 0.008546 | 0.001179 | 4.26E-13 | 25.76992 |
| rs9678909 | G | A | 0.2408 | -0.00913 | 0.001347 | 1.19E-11 | 16.81258 |
| rs1047891 | C | A | 0.3048 | -0.03315 | 0.001269 | 1.54E-150 | 289.4784 |
| rs35472707 | C | T | 0.0485 | -0.03389 | 0.002885 | 7.06E-32 | 12.74263 |
| rs7605824 | G | A | 0.3323 | -0.01125 | 0.001226 | 4.64E-20 | 37.33187 |
| rs72818972 | C | T | 0.1317 | -0.0131 | 0.001802 | 3.62E-13 | 12.08476 |
| rs4954189 | G | A | 0.491 | -0.00733 | 0.001152 | 1.99E-10 | 20.23123 |
| rs807624 | G | T | 0.4028 | 0.017418 | 0.001177 | 1.58E-49 | 105.3171 |
| rs77964389 | C | A | 0.1385 | -0.01523 | 0.001763 | 5.77E-18 | 17.80187 |
| rs1949651 | C | T | 0.4796 | -0.00807 | 0.001152 | 2.43E-12 | 24.50854 |
| rs4674331 | G | A | 0.4073 | 0.006759 | 0.001189 | 1.33E-08 | 15.58784 |
| rs3791699 | G | A | 0.2838 | -0.00808 | 0.001296 | 4.52E-10 | 15.80351 |
| rs60980181 | T | A | 0.1539 | -0.01365 | 0.001597 | 1.22E-17 | 19.04271 |
| rs74902775 | C | T | 0.1374 | 0.021055 | 0.00177 | 1.24E-32 | 33.54573 |
| rs11123169 | C | T | 0.6725 | 0.014378 | 0.001229 | 1.35E-31 | 60.24914 |
| rs4575719 | C | A | 0.6016 | 0.011363 | 0.001193 | 1.62E-21 | 43.50921 |
| rs6721675 | G | A | 0.6497 | 0.006808 | 0.001224 | 2.67E-08 | 14.08147 |
| rs2581066 | G | C | 0.7563 | 0.010287 | 0.00136 | 3.94E-14 | 21.08496 |
| rs838717 | G | A | 0.5753 | -0.0078 | 0.001165 | 2.08E-11 | 21.93623 |
| rs7584922 | C | T | 0.7866 | 0.015656 | 0.001405 | 7.83E-29 | 41.67885 |
| rs1519104 | G | A | 0.6514 | 0.009058 | 0.001226 | 1.47E-13 | 24.80279 |
| rs4666821 | G | T | 0.527 | 0.010748 | 0.001154 | 1.24E-20 | 43.24067 |
| rs2306623 | T | C | 0.6749 | 0.010945 | 0.001247 | 1.68E-18 | 33.80555 |
| rs704570 | A | G | 0.6826 | -0.0079 | 0.001238 | 1.77E-10 | 17.63799 |
| rs357466 | A | G | 0.8642 | -0.01375 | 0.001774 | 9.12E-15 | 14.10142 |
| rs13061908 | A | G | 0.505 | -0.007 | 0.001209 | 7.08E-09 | 16.75475 |
| rs6788293 | T | C | 0.587 | -0.00678 | 0.001169 | 6.63E-09 | 16.31091 |
| rs2289746 | T | C | 0.6244 | 0.008026 | 0.001191 | 1.59E-11 | 21.30173 |
| rs1274961 | T | C | 0.7806 | 0.00925 | 0.001473 | 3.39E-10 | 13.50883 |
| rs80292783 | A | C | 0.1336 | -0.01546 | 0.001692 | 6.42E-20 | 19.32714 |
| rs111746012 | T | C | 0.4637 | -0.00826 | 0.001159 | 1.02E-12 | 25.2707 |
| rs16847759 | A | G | 0.1251 | 0.01302 | 0.001767 | 1.71E-13 | 11.89005 |
| rs6438900 | C | G | 0.2693 | -0.00921 | 0.001316 | 2.69E-12 | 19.24586 |
| rs9823161 | A | G | 0.3829 | -0.0111 | 0.001218 | 7.72E-20 | 39.28127 |
| rs7221 | T | C | 0.2391 | 0.008134 | 0.001352 | 1.80E-09 | 13.16472 |
| rs10439970 | T | G | 0.3234 | -0.01541 | 0.001232 | 6.67E-36 | 68.4804 |
| rs6445387 | C | G | 0.1544 | -0.01547 | 0.001616 | 1.01E-21 | 23.94512 |
| rs35004449 | G | T | 0.2787 | 0.010755 | 0.001284 | 5.48E-17 | 28.20743 |
| rs6446277 | G | A | 0.2999 | 0.011177 | 0.001273 | 1.59E-18 | 32.39377 |
| rs2700420 | G | A | 0.4823 | -0.01471 | 0.001154 | 3.28E-37 | 81.13274 |
| rs2874616 | C | T | 0.2664 | 0.007124 | 0.001303 | 4.58E-08 | 11.68219 |
| rs56065557 | G | C | 0.3195 | -0.01622 | 0.001254 | 2.71E-38 | 72.80318 |
| rs6782349 | C | T | 0.3099 | -0.00946 | 0.001246 | 3.03E-14 | 24.68619 |
| rs34053392 | G | A | 0.3988 | 0.008244 | 0.001198 | 5.90E-12 | 22.71115 |
| rs12638420 | C | T | 0.2994 | 0.007287 | 0.001321 | 3.49E-08 | 12.75987 |
| rs28631273 | G | A | 0.2657 | 0.012918 | 0.001379 | 7.68E-21 | 34.21587 |
| rs4679215 | C | T | 0.6102 | -0.01112 | 0.001239 | 2.94E-19 | 38.28564 |
| rs310758 | C | T | 0.7215 | 0.010602 | 0.001284 | 1.52E-16 | 27.38629 |
| rs1355438 | C | T | 0.7255 | -0.02246 | 0.001291 | 8.69E-68 | 120.5571 |
| rs4561809 | G | C | 0.5447 | -0.00695 | 0.001175 | 3.24E-09 | 17.37744 |
| rs9883099 | C | A | 0.5992 | 0.006853 | 0.001175 | 5.41E-09 | 16.34807 |
| rs6599204 | C | T | 0.6104 | -0.01646 | 0.001181 | 3.83E-44 | 92.37734 |
| rs1229984 | T | C | 0.756 | 0.00824 | 0.001381 | 2.40E-09 | 13.14022 |
| rs4604059 | T | C | 0.5148 | -0.01047 | 0.001154 | 1.22E-19 | 41.07029 |
| rs55929207 | C | G | 0.5117 | -0.01176 | 0.001152 | 1.75E-24 | 52.11563 |
| rs71606723 | A | T | 0.229 | -0.01132 | 0.00137 | 1.40E-16 | 24.12192 |
| rs4861000 | A | G | 0.3817 | 0.007413 | 0.001204 | 7.41E-10 | 17.89346 |
| rs12509595 | T | C | 0.2904 | 0.017255 | 0.001286 | 4.94E-41 | 74.17238 |
| rs34241040 | T | C | 0.3492 | -0.00908 | 0.001225 | 1.27E-13 | 24.95042 |
| rs4864954 | T | C | 0.3232 | -0.00855 | 0.001317 | 8.36E-11 | 18.44977 |
| rs72719149 | T | C | 0.3094 | -0.01163 | 0.001245 | 9.45E-21 | 37.29642 |
| rs2903386 | T | C | 0.3812 | 0.008354 | 0.001185 | 1.83E-12 | 23.4287 |
| rs4699032 | C | T | 0.4989 | 0.015215 | 0.001151 | 7.19E-40 | 87.32263 |
| rs1398016 | G | A | 0.4069 | -0.0398 | 0.001174 | 7.63E-252 | 554.7551 |
| rs6839650 | C | T | 0.3046 | -0.0089 | 0.001269 | 2.33E-12 | 20.83566 |
| rs59950280 | G | A | 0.3568 | 0.009289 | 0.001221 | 2.81E-14 | 26.56042 |
| rs17485995 | G | A | 0.1764 | -0.00921 | 0.001532 | 1.83E-09 | 10.50232 |
| rs62325228 | G | T | 0.1396 | 0.01241 | 0.001661 | 7.96E-14 | 13.40839 |
| rs4647930 | C | A | 0.2563 | -0.0077 | 0.001325 | 6.32E-09 | 12.85975 |
| rs35044129 | T | A | 0.3212 | -0.00809 | 0.001233 | 5.31E-11 | 18.77692 |
| rs3774921 | C | T | 0.5359 | -0.00945 | 0.001172 | 7.57E-16 | 32.323 |
| rs278941 | G | A | 0.6626 | -0.00907 | 0.001224 | 1.25E-13 | 24.55763 |
| rs434215 | A | G | 0.7125 | 0.023419 | 0.001812 | 3.25E-38 | 68.44366 |
| rs4705067 | C | G | 0.7605 | 0.010423 | 0.001352 | 1.26E-14 | 21.65457 |
| rs7727632 | T | C | 0.8131 | 0.010736 | 0.001481 | 4.23E-13 | 15.96702 |
| rs27028 | A | G | 0.6357 | 0.008311 | 0.001197 | 3.84E-12 | 22.32757 |
| rs1045051 | T | G | 0.3174 | 0.007038 | 0.001257 | 2.13E-08 | 13.59369 |
| rs78660602 | A | G | 0.0899 | 0.024708 | 0.002018 | 1.76E-34 | 24.53996 |
| rs7736027 | A | G | 0.2888 | -0.00995 | 0.00127 | 4.73E-15 | 25.21116 |
| rs11746047 | C | T | 0.3787 | -0.00728 | 0.001204 | 1.49E-09 | 17.19586 |
| rs72759880 | G | T | 0.106 | -0.02632 | 0.00187 | 5.41E-45 | 37.54745 |
| rs35969577 | G | T | 0.391 | -0.0276 | 0.001196 | 1.02E-117 | 253.4199 |
| rs3812036 | C | T | 0.2492 | -0.0372 | 0.001353 | 1.53E-166 | 283.1433 |
| rs12520984 | G | C | 0.3158 | 0.011747 | 0.001239 | 2.49E-21 | 38.85399 |
| rs6596100 | C | T | 0.2384 | -0.01154 | 0.001351 | 1.35E-17 | 26.47795 |
| rs13157326 | G | A | 0.4473 | -0.01373 | 0.001173 | 1.23E-31 | 67.72228 |
| rs62331274 | G | C | 0.2557 | -0.00933 | 0.001461 | 1.73E-10 | 15.51309 |
| rs80138475 | C | T | 0.1078 | 0.033143 | 0.001959 | 3.43E-64 | 55.04548 |
| rs4705857 | C | T | 0.3329 | 0.008043 | 0.001224 | 5.00E-11 | 19.17797 |
| rs289232 | G | A | 0.5787 | 0.006428 | 0.001168 | 3.73E-08 | 14.7665 |
| rs3822939 | A | G | 0.5761 | 0.012916 | 0.001166 | 1.55E-28 | 59.97444 |
| rs1933736 | T | C | 0.4357 | -0.0066 | 0.001162 | 1.31E-08 | 15.89252 |
| rs9493650 | T | G | 0.25 | 0.008474 | 0.00133 | 1.89E-10 | 15.21648 |
| rs62432758 | T | C | 0.2181 | 0.010322 | 0.001415 | 2.96E-13 | 18.15562 |
| rs2608916 | T | G | 0.244 | -0.01669 | 0.00136 | 1.36E-34 | 55.518 |
| rs9463019 | A | G | 0.2121 | -0.01001 | 0.001409 | 1.20E-12 | 16.87229 |
| rs9368060 | A | G | 0.4432 | -0.00683 | 0.001161 | 4.05E-09 | 17.07589 |
| rs3127575 | C | T | 0.1155 | -0.04177 | 0.001802 | 8.93E-119 | 109.706 |
| rs72912510 | G | A | 0.195 | -0.01278 | 0.001475 | 4.68E-18 | 23.55064 |
| rs1772976 | C | T | 0.4944 | 0.007729 | 0.001168 | 3.64E-11 | 21.89649 |
| rs113990079 | G | A | 0.0782 | -0.02601 | 0.00227 | 2.10E-30 | 18.93422 |
| rs693906 | G | C | 0.1446 | -0.02026 | 0.001642 | 5.65E-35 | 37.65887 |
| rs11759908 | C | T | 0.4176 | -0.01336 | 0.001184 | 1.54E-29 | 61.95969 |
| rs9480866 | C | T | 0.1618 | 0.016667 | 0.001565 | 1.76E-26 | 30.75971 |
| rs11753995 | G | A | 0.1626 | 0.020864 | 0.001654 | 1.70E-36 | 43.35184 |
| rs9483082 | C | A | 0.2302 | -0.01097 | 0.001368 | 1.09E-15 | 22.77374 |
| rs3799354 | C | A | 0.1165 | 0.020795 | 0.001886 | 2.86E-28 | 25.0268 |
| rs1857859 | G | A | 0.2887 | 0.008078 | 0.001275 | 2.36E-10 | 16.48786 |
| rs6458868 | C | T | 0.6628 | -0.01059 | 0.001218 | 3.56E-18 | 33.77136 |
| rs881858 | G | A | 0.6915 | -0.02614 | 0.001249 | 2.62E-97 | 186.9973 |
| rs11243143 | G | A | 0.6093 | -0.0156 | 0.001266 | 6.64E-35 | 72.32672 |
| rs1087289 | G | T | 0.5788 | 0.009617 | 0.001224 | 3.90E-15 | 30.10781 |
| rs9465741 | C | A | 0.5013 | 0.010783 | 0.001168 | 2.71E-20 | 42.59724 |
| rs700753 | C | G | 0.6639 | -0.01721 | 0.001219 | 2.98E-45 | 88.94287 |
| rs6951584 | T | C | 0.7077 | 0.009232 | 0.001266 | 3.01E-13 | 22.01127 |
| rs6968865 | A | T | 0.5836 | 0.011536 | 0.00117 | 6.02E-23 | 47.28105 |
| rs12670952 | T | C | 0.5665 | -0.00819 | 0.001162 | 1.76E-12 | 24.42589 |
| rs2080140 | T | C | 0.5857 | 0.00701 | 0.001186 | 3.36E-09 | 16.96838 |
| rs3817576 | A | G | 0.5521 | 0.008382 | 0.001176 | 1.01E-12 | 25.13582 |
| rs288754 | T | C | 0.6779 | -0.01319 | 0.001328 | 2.93E-23 | 43.10562 |
| rs13245051 | A | G | 0.5288 | 0.008514 | 0.001225 | 3.65E-12 | 24.07147 |
| rs3750081 | T | G | 0.4164 | 0.011568 | 0.001185 | 1.58E-22 | 46.35564 |
| rs3757387 | T | C | 0.4237 | -0.01337 | 0.001182 | 1.16E-29 | 62.48218 |
| rs62491533 | T | C | 0.176 | 0.016006 | 0.001512 | 3.57E-26 | 32.48602 |
| rs62462688 | A | T | 0.2664 | -0.02387 | 0.001302 | 4.43E-75 | 131.3938 |
| rs17151639 | A | G | 0.2706 | 0.010459 | 0.001297 | 7.32E-16 | 25.67667 |
| rs10224210 | T | C | 0.2678 | -0.04519 | 0.001408 | 4.85E-226 | 404.1243 |
| rs17152083 | G | C | 0.0989 | -0.0166 | 0.001928 | 7.37E-18 | 13.21016 |
| rs6971211 | C | T | 0.4114 | -0.01307 | 0.001174 | 8.67E-29 | 60.02822 |
| rs59860440 | C | T | 0.3105 | 0.010605 | 0.001262 | 4.35E-17 | 30.23447 |
| rs3918226 | C | T | 0.0793 | -0.01989 | 0.002274 | 2.19E-18 | 11.17229 |
| rs2302429 | G | A | 0.2059 | 0.013884 | 0.001424 | 1.81E-22 | 31.09976 |
| rs1405809 | G | A | 0.3067 | 0.012253 | 0.001253 | 1.37E-22 | 40.67744 |
| rs6964974 | C | A | 0.599 | 0.006758 | 0.001177 | 9.47E-09 | 15.82811 |
| rs71518321 | C | A | 0.5874 | 0.010632 | 0.001666 | 1.75E-10 | 19.74293 |
| rs62435145 | G | T | 0.6203 | -0.03337 | 0.001247 | 9.70E-158 | 337.3718 |
| rs7005025 | A | C | 0.6381 | -0.01035 | 0.001216 | 1.72E-17 | 33.45628 |
| rs11784717 | T | C | 0.698 | -0.00715 | 0.001254 | 1.20E-08 | 13.69763 |
| rs3739241 | C | G | 0.6134 | 0.008291 | 0.001204 | 5.82E-12 | 22.47623 |
| rs1519844 | C | G | 0.3906 | -0.01002 | 0.00126 | 1.79E-15 | 30.12682 |
| rs28601761 | C | G | 0.3977 | -0.01483 | 0.001193 | 1.84E-35 | 73.99833 |
| rs2941476 | T | C | 0.1899 | 0.010414 | 0.001469 | 1.35E-12 | 15.46206 |
| rs4734652 | C | T | 0.1933 | -0.00895 | 0.001479 | 1.41E-09 | 11.4304 |
| rs28651387 | C | T | 0.4927 | -0.00818 | 0.001168 | 2.52E-12 | 24.50915 |
| rs2953516 | C | T | 0.2897 | 0.012804 | 0.001269 | 6.24E-24 | 41.88339 |
| rs17644096 | G | A | 0.1977 | 0.010153 | 0.001524 | 2.72E-11 | 14.07525 |
| rs7012814 | G | A | 0.4825 | 0.015681 | 0.001174 | 1.12E-40 | 89.06063 |
| rs11557049 | C | T | 0.0623 | -0.02435 | 0.002632 | 2.20E-20 | 10.00128 |
| rs12544197 | G | A | 0.4931 | -0.00756 | 0.00117 | 1.04E-10 | 20.86853 |
| rs10283362 | C | T | 0.1735 | 0.011714 | 0.001542 | 3.08E-14 | 16.54368 |
| rs2096551 | G | C | 0.4051 | -0.00832 | 0.001173 | 1.30E-12 | 24.25632 |
| rs11786896 | C | T | 0.0472 | -0.03169 | 0.002965 | 1.12E-26 | 10.28046 |
| rs34861762 | C | T | 0.3955 | -0.02579 | 0.001177 | 2.28E-106 | 229.4902 |
| rs11135735 | C | T | 0.5543 | 0.007239 | 0.001158 | 4.13E-10 | 19.29493 |
| rs10092747 | C | T | 0.765 | 0.007818 | 0.001361 | 9.36E-09 | 11.85464 |
| rs900240 | C | T | 0.6026 | 0.00651 | 0.00118 | 3.47E-08 | 14.57267 |
| rs10111039 | G | A | 0.5613 | 0.007955 | 0.001162 | 7.70E-12 | 23.06848 |
| rs4744712 | A | C | 0.6087 | 0.024644 | 0.001186 | 7.76E-96 | 205.5692 |
| rs544169 | A | G | 0.2773 | -0.01094 | 0.001287 | 1.91E-17 | 28.95219 |
| rs4745360 | A | G | 0.4626 | 0.007075 | 0.001178 | 1.89E-09 | 17.94128 |
| rs12346653 | T | C | 0.3138 | 0.007152 | 0.00126 | 1.36E-08 | 13.88443 |
| rs10758189 | T | C | 0.3495 | -0.00779 | 0.001208 | 1.10E-10 | 18.93445 |
| rs7041037 | T | C | 0.3531 | -0.00928 | 0.001208 | 1.54E-14 | 26.97407 |
| rs12377027 | A | G | 0.1738 | 0.012648 | 0.001634 | 9.86E-15 | 17.20931 |
| rs7036795 | T | C | 0.1986 | 0.009933 | 0.001453 | 8.03E-12 | 14.88403 |
| rs4837127 | C | T | 0.2408 | 0.008387 | 0.001351 | 5.33E-10 | 14.09579 |
| rs10982063 | C | T | 0.2672 | 0.007659 | 0.001305 | 4.44E-09 | 13.47993 |
| rs1321917 | G | C | 0.4246 | -0.01206 | 0.001166 | 4.36E-25 | 52.30451 |
| rs11103387 | C | T | 0.3624 | 0.012004 | 0.001227 | 1.37E-22 | 44.20347 |
| rs10819321 | G | A | 0.7556 | 0.009326 | 0.001346 | 4.24E-12 | 17.73245 |
| rs3793662 | C | T | 0.8054 | 0.011265 | 0.001485 | 3.30E-14 | 18.03909 |
| rs1258182 | C | G | 0.5022 | -0.01034 | 0.001151 | 2.76E-19 | 40.30248 |
| rs11001034 | T | C | 0.5543 | 0.00648 | 0.001167 | 2.82E-08 | 15.2307 |
| rs10786215 | C | G | 0.3849 | -0.00662 | 0.0012 | 3.47E-08 | 14.40708 |
| rs6481598 | C | G | 0.2196 | -0.01082 | 0.001391 | 7.31E-15 | 20.74113 |
| rs7075982 | T | G | 0.2161 | 0.007775 | 0.001401 | 2.89E-08 | 10.42847 |
| rs80282103 | A | T | 0.094 | -0.04272 | 0.001973 | 5.22E-104 | 79.89229 |
| rs11009954 | A | G | 0.4616 | -0.00858 | 0.001171 | 2.42E-13 | 26.65544 |
| rs1008982 | T | C | 0.3679 | 0.007767 | 0.001196 | 8.36E-11 | 19.61441 |
| rs2393730 | G | A | 0.3991 | 0.007372 | 0.001178 | 3.87E-10 | 18.79009 |
| rs7475348 | C | T | 0.4662 | 0.016204 | 0.00117 | 1.38E-43 | 95.39609 |
| rs10788623 | G | A | 0.474 | 0.010014 | 0.001169 | 1.10E-17 | 36.56416 |
| rs1589067 | C | T | 0.3553 | 0.009117 | 0.001203 | 3.48E-14 | 26.31562 |
| rs12240572 | T | A | 0.1262 | -0.01195 | 0.001769 | 1.46E-11 | 10.05469 |
| rs10994856 | G | A | 0.1787 | 0.022426 | 0.001505 | 3.45E-50 | 65.14368 |
| rs9419939 | G | A | 0.1824 | 0.015362 | 0.001512 | 2.99E-24 | 30.78867 |
| rs816850 | G | C | 0.2693 | -0.00942 | 0.001299 | 4.02E-13 | 20.71503 |
| rs2068888 | G | A | 0.4673 | -0.01503 | 0.001157 | 1.47E-38 | 83.96198 |
| rs4245596 | G | A | 0.5625 | 0.007405 | 0.00116 | 1.76E-10 | 20.04071 |
| rs945488 | C | T | 0.5353 | 0.006441 | 0.001156 | 2.55E-08 | 15.43527 |
| rs11245344 | C | T | 0.5583 | -0.01393 | 0.001162 | 3.87E-33 | 70.94152 |
| rs1783977 | T | C | 0.5823 | -0.0084 | 0.001167 | 6.34E-13 | 25.16912 |
| rs11042594 | A | G | 0.6962 | -0.01273 | 0.001256 | 3.90E-24 | 43.44353 |
| rs10790453 | T | C | 0.7911 | 0.013064 | 0.001416 | 2.84E-20 | 28.12808 |
| rs4980598 | T | C | 0.6546 | -0.01007 | 0.001215 | 1.18E-16 | 31.04 |
| rs4923535 | A | G | 0.4821 | -0.00672 | 0.001154 | 5.96E-09 | 16.90304 |
| rs4945268 | T | C | 0.2076 | 0.01328 | 0.001419 | 8.28E-21 | 28.79984 |
| rs1042752 | A | G | 0.4324 | -0.00757 | 0.001162 | 7.27E-11 | 20.83494 |
| rs61897431 | T | C | 0.3615 | -0.0127 | 0.001258 | 5.58E-24 | 47.0835 |
| rs963837 | T | C | 0.4316 | 0.031515 | 0.001181 | 5.53E-157 | 349.6977 |
| rs571856 | T | C | 0.2781 | -0.00718 | 0.001303 | 3.55E-08 | 12.19914 |
| rs61910261 | T | G | 0.4007 | -0.00736 | 0.001192 | 6.62E-10 | 18.31355 |
| rs1541940 | A | C | 0.3249 | 0.013359 | 0.001229 | 1.64E-27 | 51.8157 |
| rs81205 | A | C | 0.4892 | -0.01608 | 0.001178 | 1.88E-42 | 93.19161 |
| rs61327861 | G | A | 0.1294 | -0.01673 | 0.001847 | 1.30E-19 | 18.49457 |
| rs7931740 | G | A | 0.3126 | 0.00746 | 0.00126 | 3.18E-09 | 15.07195 |
| rs34869311 | C | A | 0.2801 | -0.00998 | 0.001282 | 7.08E-15 | 24.42957 |
| rs10767859 | C | T | 0.4275 | -0.0089 | 0.001167 | 2.44E-14 | 28.45961 |
| rs11600923 | C | T | 0.1388 | 0.012984 | 0.001779 | 2.88E-13 | 12.74008 |
| rs11604451 | C | T | 0.3299 | -0.01968 | 0.001224 | 4.01E-58 | 114.2152 |
| rs2351958 | C | A | 0.4125 | -0.00729 | 0.001172 | 4.88E-10 | 18.77008 |
| rs12808675 | G | A | 0.3679 | 0.008004 | 0.001194 | 2.01E-11 | 20.90972 |
| rs17885785 | C | T | 0.1901 | -0.01186 | 0.001504 | 3.20E-15 | 19.13515 |
| rs11062102 | T | C | 0.6099 | -0.02118 | 0.001308 | 5.93E-59 | 124.7364 |
| rs2634675 | A | G | 0.5013 | -0.01158 | 0.001167 | 3.27E-23 | 49.24412 |
| rs2277383 | T | G | 0.1442 | -0.01223 | 0.001644 | 1.01E-13 | 13.65841 |
| rs73119306 | A | G | 0.232 | 0.017327 | 0.00137 | 1.16E-36 | 56.99937 |
| rs11616030 | A | C | 0.0858 | 0.021797 | 0.002213 | 6.99E-23 | 15.21453 |
| rs704061 | T | C | 0.4585 | 0.007638 | 0.001159 | 4.37E-11 | 21.57128 |
| rs10846157 | A | C | 0.2363 | 0.019661 | 0.001355 | 1.06E-47 | 75.98232 |
| rs216247 | T | C | 0.4856 | 0.006605 | 0.001173 | 1.77E-08 | 15.85234 |
| rs7300900 | G | T | 0.2074 | -0.0109 | 0.001438 | 3.43E-14 | 18.89518 |
| rs11170566 | C | T | 0.1443 | 0.011251 | 0.001641 | 7.13E-12 | 11.60475 |
| rs836968 | C | T | 0.3191 | 0.009476 | 0.001253 | 3.97E-14 | 24.84966 |
| rs10850001 | T | A | 0.4448 | -0.00757 | 0.001177 | 1.23E-10 | 20.45251 |
| rs7979282 | G | T | 0.0952 | -0.02144 | 0.001966 | 1.13E-27 | 20.47563 |
| rs1275609 | G | A | 0.3651 | 0.010556 | 0.001213 | 3.29E-18 | 35.09898 |
| rs6416254 | G | A | 0.5447 | 0.007731 | 0.001156 | 2.26E-11 | 22.18624 |
| rs11062547 | C | T | 0.7263 | 0.008208 | 0.00131 | 3.68E-10 | 15.61509 |
| rs4396370 | G | A | 0.7438 | 0.007865 | 0.001338 | 4.18E-09 | 13.16375 |
| rs2104480 | C | G | 0.6744 | 0.010805 | 0.00123 | 1.62E-18 | 33.8633 |
| rs4771077 | A | G | 0.9047 | -0.01691 | 0.001961 | 6.47E-18 | 12.82473 |
| rs4142040 | T | C | 0.2209 | -0.00925 | 0.001411 | 5.64E-11 | 14.78096 |
| rs17575422 | T | C | 0.4611 | 0.007474 | 0.001304 | 9.98E-09 | 16.32293 |
| rs9515196 | T | G | 0.4002 | -0.00889 | 0.001178 | 4.45E-14 | 27.34447 |
| rs12585865 | T | C | 0.399 | 0.010878 | 0.001176 | 2.24E-20 | 41.03671 |
| rs7326821 | A | G | 0.1681 | -0.00985 | 0.001561 | 2.86E-10 | 11.12191 |
| rs77279628 | C | T | 0.1729 | 0.009742 | 0.001564 | 4.69E-10 | 11.09742 |
| rs9316505 | G | A | 0.564 | 0.006885 | 0.001178 | 5.10E-09 | 16.79656 |
| rs9529913 | C | T | 0.5464 | -0.01583 | 0.001156 | 1.20E-42 | 92.87958 |
| rs9530201 | C | T | 0.5026 | -0.00999 | 0.001151 | 3.96E-18 | 37.67108 |
| rs7996468 | C | T | 0.6997 | 0.008747 | 0.001256 | 3.31E-12 | 20.38075 |
| rs875908 | C | G | 0.616 | 0.007832 | 0.001201 | 7.03E-11 | 20.11141 |
| rs10142359 | A | G | 0.5254 | -0.01006 | 0.001153 | 2.62E-18 | 37.9829 |
| rs17563 | A | G | 0.522 | -0.00922 | 0.001171 | 3.63E-15 | 30.88552 |
| rs4900538 | T | C | 0.656 | 0.008672 | 0.001255 | 4.85E-12 | 21.55034 |
| rs8008655 | T | G | 0.2778 | -0.01013 | 0.001304 | 7.60E-15 | 24.2502 |
| rs10162419 | A | G | 0.3461 | 0.008048 | 0.001214 | 3.33E-11 | 19.90242 |
| rs58006834 | A | T | 0.3593 | -0.00826 | 0.001292 | 1.62E-10 | 18.82313 |
| rs7147635 | A | T | 0.4953 | -0.00782 | 0.00125 | 3.90E-10 | 19.57977 |
| rs72711990 | A | G | 0.3421 | -0.01103 | 0.001241 | 5.89E-19 | 35.60793 |
| rs11624899 | T | C | 0.3951 | -0.00765 | 0.001182 | 9.87E-11 | 20.00322 |
| rs28711639 | G | C | 0.333 | 0.011172 | 0.001222 | 5.91E-20 | 37.1597 |
| rs9652384 | G | A | 0.4628 | 0.007071 | 0.001155 | 9.13E-10 | 18.64808 |
| rs10151563 | G | A | 0.6244 | -0.00914 | 0.001189 | 1.51E-14 | 27.7166 |
| rs12588550 | C | T | 0.7102 | -0.01148 | 0.00129 | 5.44E-19 | 32.62799 |
| rs1433891 | T | C | 0.8232 | 0.01204 | 0.00151 | 1.51E-15 | 18.51793 |
| rs16962440 | T | C | 0.5699 | 0.006385 | 0.001165 | 4.23E-08 | 14.72724 |
| rs783538 | C | G | 0.3076 | 0.007265 | 0.001247 | 5.75E-09 | 14.4484 |
| rs34605751 | A | T | 0.3788 | 0.012717 | 0.001706 | 8.99E-14 | 26.15613 |
| rs11071738 | T | C | 0.4388 | 0.012064 | 0.00116 | 2.53E-25 | 53.25105 |
| rs28715410 | C | G | 0.3625 | 0.008367 | 0.001215 | 5.70E-12 | 21.92218 |
| rs4886755 | A | G | 0.4961 | -0.02322 | 0.001152 | 2.53E-90 | 203.0863 |
| rs872192 | A | G | 0.4105 | -0.0454 | 0.001187 | 0 | 708.5723 |
| rs2412608 | C | T | 0.4687 | 0.018258 | 0.001238 | 3.46E-49 | 108.2451 |
| rs62021209 | G | C | 0.229 | 0.008708 | 0.001392 | 4.00E-10 | 13.81145 |
| rs956006 | C | T | 0.3217 | 0.008367 | 0.001251 | 2.22E-11 | 19.53843 |
| rs12915294 | C | T | 0.2068 | -0.01356 | 0.001505 | 1.97E-19 | 26.66256 |
| rs17730281 | G | A | 0.2483 | 0.021193 | 0.001335 | 9.29E-57 | 94.09359 |
| rs4966019 | C | T | 0.6033 | 0.012059 | 0.001196 | 6.57E-24 | 48.66518 |
| rs11852715 | C | T | 0.7089 | 0.00972 | 0.00127 | 1.92E-14 | 24.19175 |
| rs351242 | G | A | 0.759 | -0.01046 | 0.00137 | 2.26E-14 | 21.32615 |
| rs11150220 | A | G | 0.7654 | -0.00826 | 0.001381 | 2.19E-09 | 12.85546 |
| rs11117317 | T | C | 0.6402 | -0.00726 | 0.001202 | 1.49E-09 | 16.83464 |
| rs7188071 | T | C | 0.6614 | -0.00883 | 0.001217 | 3.84E-13 | 23.61459 |
| rs11865979 | T | C | 0.2719 | -0.0087 | 0.0013 | 2.16E-11 | 17.74746 |
| rs9937801 | T | C | 0.4314 | -0.00839 | 0.001179 | 1.14E-12 | 24.8146 |
| rs12935539 | T | C | 0.2375 | -0.01763 | 0.001373 | 8.83E-38 | 59.78895 |
| rs11865670 | A | G | 0.4015 | -0.00707 | 0.00127 | 2.62E-08 | 14.8838 |
| rs728538 | T | G | 0.1719 | -0.01153 | 0.001534 | 5.56E-14 | 16.09154 |
| rs564381127 | T | A | 0.1429 | -0.02049 | 0.002364 | 4.40E-18 | 18.40502 |
| rs9934475 | G | A | 0.3942 | 0.008897 | 0.00118 | 4.77E-14 | 27.13911 |
| rs77924615 | G | A | 0.197 | 0.058611 | 0.001468 | 0 | 504.6094 |
| rs154656 | T | A | 0.4199 | -0.017 | 0.00117 | 7.27E-48 | 102.929 |
| rs7206646 | G | A | 0.1816 | 0.010972 | 0.001496 | 2.25E-13 | 15.98376 |
| rs4783809 | G | T | 0.4605 | -0.00945 | 0.001178 | 1.09E-15 | 31.92824 |
| rs193538 | G | T | 0.6887 | -0.00827 | 0.001243 | 2.89E-11 | 18.97355 |
| rs3961283 | G | A | 0.7391 | 0.013995 | 0.00133 | 6.63E-26 | 42.72312 |
| rs4786429 | C | T | 0.7086 | -0.0113 | 0.001368 | 1.44E-16 | 28.18342 |
| rs3736164 | T | G | 0.6911 | 0.007954 | 0.001264 | 3.09E-10 | 16.914 |
| rs1010269 | A | G | 0.7657 | 0.038089 | 0.001378 | 4.23E-168 | 274.0678 |
| rs2350633 | A | G | 0.4746 | -0.01027 | 0.001169 | 1.63E-18 | 38.44566 |
| rs57437384 | A | G | 0.2942 | -0.00693 | 0.001263 | 4.09E-08 | 12.50337 |
| rs57937546 | A | G | 0.3208 | -0.00837 | 0.00126 | 3.09E-11 | 19.22493 |
| rs227724 | A | T | 0.3345 | -0.00947 | 0.00124 | 2.29E-14 | 25.94009 |
| rs67597968 | A | G | 0.0808 | -0.02066 | 0.002271 | 9.20E-20 | 12.2955 |
| rs2252281 | T | C | 0.3733 | -0.0219 | 0.001192 | 2.50E-75 | 157.8245 |
| rs10491128 | G | A | 0.2557 | 0.028988 | 0.001322 | 1.37E-106 | 183.0623 |
| rs62084703 | C | T | 0.2192 | 0.008394 | 0.001515 | 3.04E-08 | 10.50209 |
| rs740755 | G | C | 0.2785 | -0.01101 | 0.001303 | 2.89E-17 | 28.70221 |
| rs59853503 | C | T | 0.162 | 0.0123 | 0.001654 | 1.05E-13 | 15.00911 |
| rs12940987 | G | A | 0.7627 | -0.02444 | 0.001354 | 6.95E-73 | 118.0338 |
| rs8076195 | G | C | 0.5226 | -0.00759 | 0.001169 | 8.64E-11 | 21.0108 |
| rs9894634 | C | T | 0.5694 | -0.01005 | 0.001289 | 6.39E-15 | 29.80392 |
| rs4245230 | A | G | 0.5373 | 0.010358 | 0.001172 | 1.00E-18 | 38.81236 |
| rs1790861 | T | C | 0.2727 | -0.00867 | 0.001311 | 3.72E-11 | 17.35773 |
| rs8096658 | C | G | 0.4586 | -0.03037 | 0.001214 | 3.11E-138 | 311.0918 |
| rs1719934 | A | G | 0.4364 | -0.01476 | 0.001161 | 5.14E-37 | 79.48183 |
| rs9960646 | G | A | 0.245 | 0.014905 | 0.001339 | 8.47E-29 | 45.87079 |
| rs151245 | G | T | 0.5959 | -0.01194 | 0.001182 | 5.58E-24 | 49.1204 |
| rs8101667 | T | C | 0.6387 | -0.02641 | 0.001198 | 1.19E-107 | 224.2241 |
| rs76301301 | T | G | 0.1866 | 0.009032 | 0.00151 | 2.23E-09 | 10.85553 |
| rs56255430 | A | C | 0.0795 | -0.01957 | 0.002128 | 3.75E-20 | 12.37448 |
| rs34647824 | A | C | 0.2772 | 0.00883 | 0.001313 | 1.74E-11 | 18.12841 |
| rs76881507 | A | T | 0.2197 | 0.008262 | 0.00139 | 2.82E-09 | 12.1057 |
| rs281380 | T | C | 0.4036 | 0.011728 | 0.001194 | 8.79E-23 | 46.47257 |
| rs34188292 | G | C | 0.2816 | -0.00825 | 0.001337 | 6.87E-10 | 15.39792 |
| rs74862090 | C | T | 0.1151 | 0.016115 | 0.00201 | 1.07E-15 | 13.09914 |
| rs10423928 | T | A | 0.2013 | -0.00909 | 0.001442 | 2.94E-10 | 12.77078 |
| rs75472426 | C | T | 0.1293 | -0.01317 | 0.001784 | 1.52E-13 | 12.28007 |
| rs7250933 | G | C | 0.5472 | 0.009438 | 0.001332 | 1.36E-12 | 24.89638 |
| rs1469024 | C | T | 0.6479 | -0.01018 | 0.001208 | 3.37E-17 | 32.44718 |
| rs6603079 | C | T | 0.574 | -0.00686 | 0.001164 | 3.75E-09 | 16.99508 |
| rs2762943 | T | G | 0.9226 | -0.02686 | 0.002414 | 9.17E-29 | 17.68573 |
| rs6141766 | A | G | 0.2308 | 0.009008 | 0.001386 | 8.03E-11 | 15.00152 |
| rs17216707 | T | C | 0.1919 | 0.024939 | 0.001489 | 6.16E-63 | 86.96903 |
| rs62187537 | C | T | 0.0672 | 0.022198 | 0.002434 | 7.51E-20 | 10.4275 |
| rs58625759 | C | A | 0.1911 | 0.012824 | 0.001488 | 6.64E-18 | 22.97772 |
| rs1970576 | C | T | 0.198 | -0.00939 | 0.001445 | 8.14E-11 | 13.41011 |
| rs8114057 | G | A | 0.4618 | -0.0076 | 0.001187 | 1.54E-10 | 20.36707 |
| rs2076668 | G | A | 0.363 | -0.01971 | 0.001214 | 3.30E-59 | 121.7682 |
| rs1041606 | C | T | 0.2325 | -0.00929 | 0.001364 | 1.00E-11 | 16.53177 |
| rs11698103 | G | C | 0.2377 | 0.01202 | 0.001446 | 9.25E-17 | 25.0502 |
| rs6039147 | C | T | 0.2474 | 0.00827 | 0.00143 | 7.34E-09 | 12.45385 |
| rs2314639 | C | T | 0.239 | -0.01361 | 0.001425 | 1.27E-21 | 33.19021 |
| rs2235826 | T | A | 0.8048 | -0.01516 | 0.001476 | 9.23E-25 | 33.16544 |
| rs219778 | A | G | 0.2655 | 0.012828 | 0.001378 | 1.27E-20 | 33.81334 |
| rs2834321 | G | A | 0.1736 | -0.01661 | 0.00152 | 8.47E-28 | 34.26576 |
| rs2823139 | G | A | 0.3345 | -0.01722 | 0.001224 | 5.81E-45 | 88.14279 |
| rs6517422 | G | A | 0.2926 | -0.01265 | 0.001266 | 1.72E-23 | 41.29881 |
| rs738683 | A | G | 0.7439 | 0.008404 | 0.001326 | 2.30E-10 | 15.3157 |
| rs738527 | T | C | 0.6939 | -0.01482 | 0.001255 | 3.77E-32 | 59.18222 |
| rs45462093 | A | G | 0.2155 | -0.00885 | 0.00145 | 1.04E-09 | 12.5939 |
| rs5763646 | T | C | 0.4261 | 0.012095 | 0.001164 | 2.78E-25 | 52.78863 |
| rs133872 | C | G | 0.4748 | -0.00641 | 0.00117 | 4.21E-08 | 14.98811 |
| rs112880707 | C | T | 0.1295 | 0.02605 | 0.001742 | 1.56E-50 | 50.39239 |
| rs6001787 | G | A | 0.2435 | -0.00994 | 0.001438 | 4.88E-12 | 17.58624 |
| rs5760006 | C | T | 0.8304 | 0.009977 | 0.001556 | 1.46E-10 | 11.57343 |
| rs132645 | G | A | 0.8276 | 0.013807 | 0.001531 | 1.90E-19 | 23.21191 |
| rs2267375 | G | T | 0.5842 | -0.01453 | 0.00117 | 2.04E-35 | 74.94452 |

SNP, single nucleotide polymorphisms; eGFR, estimated glomerular filtration rate.

**Table S3. 20 index SNPs represented genetically predicted CKD.**

| **SNP** | **effect_allele** | **other_allele** | **eaf** | **beta** | **se** | **pval** | **F-statistic** |
| --- | --- | --- | --- | --- | --- | --- | --- |
| rs2484639 | A | G | 0.51 | -0.0774 | 0.0092 | 4.00E-17 | 35.377951 |
| rs13391258 | T | C | 0.24 | -0.06 | 0.0108 | 2.77E-08 | 11.259476 |
| rs2580350 | A | G | 0.55 | 0.055 | 0.0098 | 2.00E-08 | 15.591601 |
| rs6532328 | T | C | 0.23 | -0.0904 | 0.011 | 2.07E-16 | 23.923232 |
| rs1458038 | T | C | 0.31 | -0.059 | 0.01 | 3.64E-09 | 14.892117 |
| rs700221 | A | G | 0.59 | -0.0719 | 0.0098 | 2.19E-13 | 26.043132 |
| rs35716097 | T | C | 0.32 | 0.0785 | 0.0105 | 7.65E-14 | 24.325948 |
| rs881858 | A | G | 0.7 | 0.0616 | 0.0101 | 1.07E-09 | 15.62357 |
| rs9474801 | A | G | 0.34 | 0.0522 | 0.0096 | 5.40E-08 | 13.269714 |
| rs11761603 | T | C | 0.3 | -0.0674 | 0.0119 | 1.48E-08 | 13.473658 |
| rs10224002 | A | G | 0.72 | -0.1083 | 0.0102 | 2.47E-26 | 45.458631 |
| rs4871907 | A | C | 0.55 | -0.0628 | 0.0097 | 9.53E-11 | 20.749038 |
| rs1889937 | A | G | 0.63 | -0.0624 | 0.01 | 4.38E-10 | 18.153319 |
| rs3925584 | T | C | 0.56 | 0.08 | 0.0092 | 3.45E-18 | 37.265494 |
| rs77713116 | C | G | 0.65 | -0.0752 | 0.0116 | 9.01E-11 | 19.122584 |
| rs7178881 | A | C | 0.41 | -0.0544 | 0.0092 | 3.36E-09 | 16.916148 |
| rs1049518 | A | G | 0.38 | 0.0788 | 0.0094 | 5.16E-17 | 33.115409 |
| rs17730281 | A | G | 0.23 | -0.0869 | 0.011 | 2.79E-15 | 22.106547 |
| rs77924615 | A | G | 0.2 | -0.2237 | 0.0128 | 2.16E-68 | 97.757146 |
| rs8096658 | C | G | 0.51 | -0.064 | 0.011 | 5.95E-09 | 16.919375 |

SNP, single nucleotide polymorphisms; CKD, chronic kidney disease

**Table S4. 92 index SNPs represented genetically predicted atrial fibrillation.**

| **SNP** | **other_allele** | **effect_allele** | **eaf** | **beta** | **se** | **pval** | **F-statistic** |
| --- | --- | --- | --- | --- | --- | --- | --- |
| rs284277 | A | C | 0.3826 | 0.0422 | 0.0069 | 9.60E-10 | 17.67156 |
| rs7529220 | T | C | 0.8469 | 0.0621 | 0.0098 | 2.35E-10 | 10.41291 |
| rs2885697 | T | G | 0.3518 | 0.0439 | 0.007 | 3.58E-10 | 17.93802 |
| rs72694603 | T | C | 0.6853 | 0.0553 | 0.0072 | 1.58E-14 | 25.44501 |
| rs4073778 | A | C | 0.4361 | -0.0486 | 0.0067 | 4.05E-13 | 25.87922 |
| rs6689306 | A | G | 0.5872 | -0.046 | 0.0068 | 1.34E-11 | 22.18513 |
| rs11264280 | T | C | 0.667 | -0.1347 | 0.0071 | 2.92E-80 | 159.9135 |
| rs72700114 | C | G | 0.9244 | -0.2021 | 0.013 | 1.69E-54 | 33.78089 |
| rs577676 | T | C | 0.5617 | 0.0923 | 0.0067 | 3.55E-43 | 93.45407 |
| rs10753933 | T | G | 0.5518 | -0.0609 | 0.0067 | 9.94E-20 | 40.86814 |
| rs6546620 | T | C | 0.7999 | 0.0602 | 0.0086 | 2.56E-12 | 15.68609 |
| rs2540949 | A | T | 0.3847 | -0.0659 | 0.0068 | 3.29E-22 | 44.46415 |
| rs6747542 | T | C | 0.4642 | -0.0554 | 0.0067 | 1.35E-16 | 34.01114 |
| rs7574892 | A | G | 0.5153 | -0.0552 | 0.0067 | 1.74E-16 | 33.90823 |
| rs2288327 | A | G | 0.1564 | 0.0919 | 0.0089 | 5.38E-25 | 28.13619 |
| rs56326533 | T | C | 0.3919 | 0.0685 | 0.0068 | 7.23E-24 | 48.36856 |
| rs35544454 | A | T | 0.1918 | -0.0589 | 0.0087 | 1.29E-11 | 14.21002 |
| rs4642101 | T | G | 0.6397 | 0.0706 | 0.0069 | 1.43E-24 | 48.26156 |
| rs73041705 | T | C | 0.2985 | -0.0443 | 0.0073 | 1.29E-09 | 15.42302 |
| rs6790396 | C | G | 0.5959 | 0.0627 | 0.0068 | 2.95E-20 | 40.94736 |
| rs34080181 | A | G | 0.621 | 0.0446 | 0.0069 | 1.02E-10 | 19.66708 |
| rs62254082 | T | C | 0.3865 | 0.0404 | 0.007 | 7.86E-09 | 15.79671 |
| rs6771054 | T | C | 0.4035 | -0.0457 | 0.0068 | 1.81E-11 | 21.74237 |
| rs10804493 | A | G | 0.3495 | -0.0558 | 0.007 | 1.57E-15 | 28.89403 |
| rs1278493 | A | G | 0.4355 | 0.0389 | 0.0068 | 1.06E-08 | 16.0905 |
| rs7612445 | T | G | 0.8121 | -0.0493 | 0.0084 | 4.38E-09 | 10.51248 |
| rs60902112 | T | C | 0.7738 | -0.0445 | 0.0079 | 1.77E-08 | 11.10762 |
| rs1458038 | T | C | 0.6913 | -0.0434 | 0.0072 | 1.66E-09 | 15.50791 |
| rs976568 | T | G | 0.3374 | 0.1104 | 0.0072 | 4.58E-53 | 105.134 |
| rs17042059 | A | G | 0.8924 | -0.4029 | 0.0102 | 0 | 299.7239 |
| rs6838973 | T | C | 0.5594 | 0.1514 | 0.0067 | 4.64E-113 | 251.7702 |
| rs10520260 | A | G | 0.3214 | -0.0457 | 0.0073 | 3.84E-10 | 17.09554 |
| rs6596717 | A | C | 0.3951 | 0.0404 | 0.0068 | 2.83E-09 | 16.8722 |
| rs337705 | T | G | 0.3749 | 0.0564 | 0.0068 | 1.09E-16 | 32.24394 |
| rs1838747 | A | G | 0.4954 | 0.0391 | 0.0067 | 5.35E-09 | 17.02721 |
| rs17171711 | T | C | 0.8225 | -0.1086 | 0.0087 | 9.27E-36 | 45.4992 |
| rs6580277 | A | G | 0.2369 | 0.067 | 0.0079 | 2.23E-17 | 26.00651 |
| rs6882776 | A | G | 0.7165 | 0.0711 | 0.0074 | 7.39E-22 | 37.50507 |
| rs10073154 | C | G | 0.4875 | -0.0445 | 0.0068 | 5.98E-11 | 21.39976 |
| rs73366713 | A | G | 0.8604 | 0.1035 | 0.0099 | 1.40E-25 | 26.25647 |
| rs34969716 | A | G | 0.6949 | -0.0702 | 0.0078 | 2.26E-19 | 34.34734 |
| rs3176326 | A | G | 0.8018 | 0.0626 | 0.0085 | 1.78E-13 | 17.23919 |
| rs2031522 | A | G | 0.3764 | -0.0436 | 0.0068 | 1.44E-10 | 19.29959 |
| rs4946333 | A | G | 0.4897 | 0.0639 | 0.0066 | 3.60E-22 | 46.85095 |
| rs72966339 | T | C | 0.6321 | 0.0616 | 0.0069 | 4.36E-19 | 37.07009 |
| rs117984853 | T | G | 0.8987 | -0.1228 | 0.012 | 1.41E-24 | 19.06758 |
| rs55734480 | A | G | 0.7506 | -0.0548 | 0.0078 | 2.13E-12 | 18.48051 |
| rs6462079 | A | G | 0.2792 | -0.0466 | 0.0076 | 8.70E-10 | 15.13249 |
| rs35005436 | T | C | 0.1551 | 0.0612 | 0.0097 | 2.80E-10 | 10.43302 |
| rs56201652 | A | G | 0.733 | 0.0531 | 0.0075 | 1.44E-12 | 19.62091 |
| rs11773845 | A | C | 0.4144 | -0.1054 | 0.0067 | 9.22E-56 | 120.1247 |
| rs7789146 | A | G | 0.8213 | 0.0584 | 0.0087 | 1.91E-11 | 13.2266 |
| rs7508 | A | G | 0.2891 | -0.0711 | 0.0075 | 2.54E-21 | 36.9418 |
| rs6994744 | A | C | 0.4954 | 0.0405 | 0.0066 | 8.44E-10 | 18.82619 |
| rs10821415 | A | C | 0.5868 | -0.0821 | 0.0067 | 1.60E-34 | 72.81938 |
| rs2274115 | A | G | 0.7003 | 0.0487 | 0.0076 | 1.48E-10 | 17.23606 |
| rs12245149 | A | C | 0.5261 | 0.047 | 0.0067 | 2.30E-12 | 24.53808 |
| rs7915134 | T | C | 0.8561 | 0.1168 | 0.0095 | 9.67E-35 | 37.24505 |
| rs10458662 | T | G | 0.1722 | 0.0544 | 0.0088 | 6.34E-10 | 10.89494 |
| rs11191116 | T | C | 0.652 | 0.041 | 0.007 | 4.71E-09 | 15.56805 |
| rs11598047 | A | G | 0.1621 | 0.1537 | 0.009 | 2.17E-65 | 79.23197 |
| rs34936990 | A | G | 0.8793 | -0.1294 | 0.0101 | 1.41E-37 | 34.84287 |
| rs4757877 | A | G | 0.7552 | -0.0723 | 0.0078 | 1.87E-20 | 31.76898 |
| rs4935786 | A | T | 0.2673 | 0.0463 | 0.0079 | 4.61E-09 | 13.45451 |
| rs76097649 | A | G | 0.9067 | -0.1151 | 0.0124 | 1.66E-20 | 14.57766 |
| rs10842383 | T | C | 0.8522 | 0.0988 | 0.0095 | 2.48E-25 | 27.24729 |
| rs17380837 | T | C | 0.693 | 0.0501 | 0.0072 | 3.44E-12 | 20.6025 |
| rs2860482 | A | C | 0.726 | -0.054 | 0.0076 | 1.20E-12 | 20.08561 |
| rs71454237 | A | G | 0.791 | 0.062 | 0.0084 | 1.57E-13 | 18.01293 |
| rs775498 | A | G | 0.2798 | 0.0423 | 0.0074 | 1.09E-08 | 13.169 |
| rs12426679 | T | C | 0.4722 | 0.0391 | 0.0067 | 5.35E-09 | 16.97601 |
| rs883079 | T | C | 0.2926 | -0.0981 | 0.0074 | 4.12E-40 | 72.75683 |
| rs6560886 | T | C | 0.7884 | 0.051 | 0.009 | 1.46E-08 | 10.71399 |
| rs9506925 | T | C | 0.7331 | -0.0449 | 0.0075 | 2.14E-09 | 14.02546 |
| rs35569628 | T | C | 0.223 | -0.0452 | 0.008 | 1.60E-08 | 11.06258 |
| rs28631169 | T | C | 0.8018 | -0.0522 | 0.0084 | 5.16E-10 | 12.27401 |
| rs11156751 | T | C | 0.2853 | 0.0719 | 0.0077 | 9.85E-21 | 35.55871 |
| rs10141892 | T | C | 0.5833 | -0.0452 | 0.0068 | 2.99E-11 | 21.47893 |
| rs2738413 | A | G | 0.5049 | -0.0778 | 0.0067 | 3.58E-31 | 67.41638 |
| rs74884082 | T | C | 0.7505 | 0.0493 | 0.0078 | 2.61E-10 | 14.96102 |
| rs7172038 | T | G | 0.1597 | 0.112 | 0.0089 | 2.58E-36 | 42.50523 |
| rs2759301 | A | G | 0.5458 | -0.039 | 0.0067 | 5.85E-09 | 16.79951 |
| rs4965430 | C | G | 0.6136 | -0.0441 | 0.0069 | 1.64E-10 | 19.37041 |
| rs77316573 | T | C | 0.8009 | -0.0529 | 0.0089 | 2.78E-09 | 11.26717 |
| rs2359171 | A | T | 0.824 | -0.1746 | 0.0086 | 1.23E-91 | 119.5669 |
| rs1563304 | T | C | 0.822 | -0.0644 | 0.0092 | 2.56E-12 | 14.33914 |
| rs7224711 | T | C | 0.4778 | 0.0365 | 0.0066 | 3.20E-08 | 15.26218 |
| rs9953366 | T | C | 0.6631 | 0.049 | 0.0073 | 1.92E-11 | 20.13094 |
| rs8088085 | A | C | 0.4646 | -0.0365 | 0.0067 | 5.10E-08 | 14.76485 |
| rs2834618 | T | G | 0.1056 | -0.0944 | 0.0112 | 3.50E-17 | 13.41957 |
| rs464901 | T | C | 0.3353 | -0.0508 | 0.0072 | 1.72E-12 | 22.19015 |
| rs133885 | A | G | 0.5623 | -0.0405 | 0.0068 | 2.59E-09 | 17.46117 |

SNP, single nucleotide polymorphisms.

**Table S5. 19 index SNPs represented genetically predicted coronary artery disease.**

| **SNP** | **effect_allele** | **other_allele** | **eaf** | **beta** | **se** | **pval** | **F-statistic** |
| --- | --- | --- | --- | --- | --- | --- | --- |
| rs10176176 | T | A | 0.470215 | 0.064649 | 0.010257 | 2.92E-10 | 19.79453 |
| rs10455872 | G | A | 0.054356 | 0.284774 | 0.026592 | 9.23E-27 | 11.79047 |
| rs10947786 | A | G | 0.207597 | -0.07187 | 0.012758 | 1.77E-08 | 10.44067 |
| rs113113862 | A | G | 0.218279 | -0.07545 | 0.012693 | 2.78E-09 | 12.05892 |
| rs11556924 | T | C | 0.29769 | -0.06892 | 0.012552 | 4.00E-08 | 12.60656 |
| rs1332329 | C | A | 0.362018 | 0.079145 | 0.010828 | 2.68E-13 | 24.68195 |
| rs1870634 | G | T | 0.617875 | 0.069696 | 0.010732 | 8.35E-11 | 19.91688 |
| rs2019090 | T | A | 0.642344 | -0.06546 | 0.011096 | 3.65E-09 | 15.99289 |
| rs2327426 | C | T | 0.297863 | -0.0625 | 0.01097 | 1.22E-08 | 13.57883 |
| rs2505083 | C | T | 0.394508 | 0.061205 | 0.010566 | 6.93E-09 | 16.03149 |
| rs2681472 | G | A | 0.19414 | 0.072705 | 0.012505 | 6.11E-09 | 10.57674 |
| rs35700460 | G | A | 0.646502 | 0.08183 | 0.012191 | 1.91E-11 | 20.59624 |
| rs4773141 | G | C | 0.345079 | 0.080221 | 0.012879 | 4.69E-10 | 17.53947 |
| rs4977574 | G | A | 0.482238 | 0.188677 | 0.010292 | 4.58E-75 | 167.973 |
| rs532436 | A | G | 0.18758 | 0.110862 | 0.013082 | 2.37E-17 | 21.89023 |
| rs653178 | T | C | 0.558224 | -0.07706 | 0.011579 | 2.84E-11 | 21.84472 |
| rs7173743 | C | T | 0.432422 | -0.06397 | 0.010401 | 7.75E-10 | 18.56746 |
| rs7528419 | G | A | 0.201938 | -0.10131 | 0.01262 | 9.93E-16 | 20.77399 |
| rs9349379 | G | A | 0.41079 | 0.130965 | 0.01065 | 9.37E-35 | 73.2327 |

SNP, single nucleotide polymorphisms.

**Table S6. 9 index SNPs represented genetically predicted heart failure.**

| **SNP** | **effect_allele** | **other_allele** | **eaf** | **beta** | **se** | **pval** | **F-statistic** |
| --- | --- | --- | --- | --- | --- | --- | --- |
| rs17617337 | T | C | 0.2208 | -0.0561 | 0.0095 | 3.52E-09 | 11.99944 |
| rs7859727 | T | C | 0.4775 | 0.0623 | 0.0078 | 1.38E-15 | 31.8339 |
| rs2634071 | T | C | 0.1763 | 0.0923 | 0.0101 | 6.33E-20 | 24.25615 |
| rs600038 | T | C | 0.7909 | -0.0569 | 0.0096 | 3.08E-09 | 11.61962 |
| rs4766578 | A | T | 0.5287 | -0.0433 | 0.0079 | 4.23E-08 | 14.97146 |
| rs56094641 | A | G | 0.5842 | -0.0454 | 0.008 | 1.39E-08 | 15.64638 |
| rs11745324 | A | G | 0.2277 | -0.0528 | 0.0095 | 2.73E-08 | 10.86435 |
| rs4135240 | T | C | 0.6589 | 0.0486 | 0.0084 | 7.22E-09 | 15.04704 |
| rs660240 | T | C | 0.2128 | -0.0611 | 0.0097 | 3.00E-10 | 13.29323 |

SNP, single nucleotide polymorphisms.

**Table S7. 7 index SNPs represented genetically predicted any stroke.**

| **SNP** | **effect_allele** | **other_allele** | **eaf** | **beta** | **se** | **pval** | **F-statistic** |
| --- | --- | --- | --- | --- | --- | --- | --- |
| rs2129977 | A | G | 0.2119 | 0.0833 | 0.0111 | 6.17E-14 | 18.8106 |
| rs4942561 | T | G | 0.7581 | 0.064 | 0.0107 | 2.21E-09 | 13.12187 |
| rs2107595 | A | G | 0.1671 | 0.0803 | 0.0121 | 3.22E-11 | 12.25941 |
| rs1537375 | T | C | 0.4979 | -0.0519 | 0.0091 | 1.18E-08 | 16.26403 |
| rs11242678 | T | C | 0.2551 | 0.0643 | 0.0105 | 9.14E-10 | 14.25259 |
| rs11587860 | C | G | 0.3545 | -0.0689 | 0.0098 | 2.06E-12 | 22.62293 |
| rs10774624 | A | G | 0.5285 | -0.0654 | 0.0094 | 3.46E-12 | 24.12559 |

SNP, single nucleotide polymorphisms.

**Table S8. 9 index SNPs represented genetically predicted any ischemic stroke.**

| **SNP** | **effect_allele** | **other_allele** | **eaf** | **beta** | **se** | **pval** | **F-statistic** |
| --- | --- | --- | --- | --- | --- | --- | --- |
| rs4942561 | T | G | 0.759 | 0.0655 | 0.0116 | 1.64E-08 | 11.66447 |
| rs2107595 | A | G | 0.1673 | 0.0882 | 0.0132 | 2.36E-11 | 12.43982 |
| rs3184504 | T | C | 0.4722 | 0.0779 | 0.0101 | 1.23E-14 | 29.6541 |
| rs34311906 | T | C | 0.5976 | -0.0649 | 0.0113 | 9.28E-09 | 15.86517 |
| rs2758612 | T | C | 0.6453 | 0.0653 | 0.0111 | 4.03E-09 | 15.84335 |
| rs11242678 | T | C | 0.255 | 0.0723 | 0.0114 | 2.27E-10 | 15.28292 |
| rs2634074 | A | T | 0.7877 | -0.0941 | 0.0121 | 7.43E-15 | 20.22867 |
| rs635634 | T | C | 0.1921 | 0.0772 | 0.0134 | 8.35E-09 | 10.30263 |
| rs2066864 | A | G | 0.2452 | 0.0634 | 0.0115 | 3.53E-08 | 11.25056 |

SNP, single nucleotide polymorphisms.

**Table S9. The results of pleiotropy test, Cochrane’s Q and MR-PRESSO for kidney function on CVDs.**

| Exposure | Outcome | *P* for pleiotropy^a^ | *P* for Cochrane’s Q | MR-PRESSO (Raw) | | | MR-PRESSO (Outlier-corrected) | | | *P* for global test | *P* for distortion test^b^ |
| --- | --- | --- | --- | --- | --- | --- | --- | --- | --- | --- | --- |
|  |  |  |  | **OR** | **95% Cl** | ***P*** | **OR** | **95% Cl** | ***P*** |  |  |
| eGFR | AF | 0.460 | <0.001 | 0.994 | 0.914-1.082 | 0.895 | 0.979 | 0.908-1.055 | 0.581 | <0.001 | 0.803 |
|  | CAD | 0.215 | <0.001 | 1.030 | 0.917-1.157 | 0.615 | 0.996 | 0.892-1.111 | 0.941 | <0.001 | 0.053 |
|  | HF | 0.471 | <0.001 | 1.049 | 0.969-1.136 | 0.240 | 1.067 | 0.987-1.153 | 0.102 | <0.001 | 0.740 |
|  | AS | 0.367 | 0.342 | 0.923 | 0.849-1.004 | 0.061 | NA | NA | NA | <0.001 | NA |
|  | AIS | 0.432 | 0.106 | 0.918 | 0.839-1.005 | 0.065 | NA | NA | NA | 0.001 | NA |
| CKD | AF | 0.587 | 0.009 | 1.013 | 0.956-1.073 | 0.673 | 1.030 | 0.980-1.083 | 0.260 | 0.015 | 0.608 |
|  | CAD | 0.168 | 0.013 | 0.982 | 0.899-1.073 | 0.690 | 1.005 | 0.942-1.072 | 0.889 | 0.078 | 0.018 |
|  | HF | 0.085 | 0.437 | 1.014 | 0.967-1.062 | 0.582 | NA | NA | NA | 0.410 | NA |
|  | AS | 0.563 | 0.451 | 1.063 | 0.995-1.135 | 0.067 | NA | NA | NA | 0.490 | NA |
|  | AIS | 0.997 | 0.518 | 1.063 | 0.988-1.148 | 0.153 | NA | NA | NA | 0.558 | NA |

^a^ P-values for pleiotropy were derived from MR-Egger test and *P*-value<0.05 indicates a possible pleiotropic effect.

^b^ P-values for distortion were derived from MR-PRESSO test and *P*-value<0.05 indicates a difference between estimates before and after outlier removal.

eGFR, estimated glomerular filtration rate; CKD, chronic kidney disease; CVD, cardiovascular disease; AF, atrial fibrillation; CAD, coronary artery disease; HF, heart failure; AS, any stroke; AIS, any ischemic stroke.

**Table S10. The results of pleiotropy test, Cochrane’s Q and MR-PRESSO for CVDs on kidney function.**

| Exposure | Outcome | *P* for pleiotropy^a^ | *P* for Cochrane’s Q | MR-PRESSO (Raw) | | | MR-PRESSO (Outlier-corrected) | | | *P* for global test | *P* for distortion test^b^ |
| --- | --- | --- | --- | --- | --- | --- | --- | --- | --- | --- | --- |
|  |  |  |  | **OR/beta** | **95% Cl** | ***P*** | **OR/beta** | **95% Cl** | ***P*** |  |  |
| AF | eGFR | 0.912 | <0.001 | -0.007 | -0.014 to 0.000 | 0.069 | -0.006 | -0.012 to -0.001 | 0.036 | <0.001 | 0.870 |
|  | CKD | 0.243 | 0.026 | 1.050 | 1.016-1.085 | 0.005 | 1.055 | 1.024-1.086 | 0.001 | 0.031 | 0.775 |
| CAD | eGFR | 0.053 | <0.001 | -0.001 | -0.023 to 0.021 | 0.919 | 0.003 | -0.012 to 0.017 | 0.737 | <0.001 | 0.569 |
|  | CKD | 0.556 | <0.001 | 0.994 | 0.900-1.097 | 0.901 | 0.986 | 0.910-1.068 | 0.731 | <0.001 | 0.905 |
| HF | eGFR | 0.956 | <0.001 | -0.056 | -0.090 to -0.022 | 0.024 | -0.065 | -0.094 to -0.036 | 0.012 | 0.016 | 0.540 |
|  | CKD | 0.986 | <0.001 | 1.115 | 0.871-1.427 | 0.418 | 1.290 | 1.081-1.539 | 0.037 | 0.001 | 0.550 |
| AS | eGFR | 0.428 | 0.086 | -0.057 | -0.079 to -0.036 | 0.003 | NA | NA | NA | 0.199 | NA |
|  | CKD | 0.634 | 0.197 | 1.332 | 1.162-1.528 | 0.006 | NA | NA | NA | 0.260 | NA |
| AIS | eGFR | 0.092 | 0.059 | -0.029 | -0.050 to -0.009 | 0.039 | NA | NA | NA | 0.111 | NA |
|  | CKD | 0.407 | 0.006 | 1.197 | 1.023-1.400 | 0.036 | 1.185 | 1.065-1.318 | 0.020 | 0.009 | 0.787 |

^a^ P-values for pleiotropy were derived from MR-Egger test and *P*-value<0.05 indicates a possible pleiotropic effect.

^b^ P-values for distortion were derived from MR-PRESSO test and *P*-value<0.05 indicates a difference between estimates before and after outlier removal.

eGFR, estimated glomerular filtration rate; CKD, chronic kidney disease; CVD, cardiovascular disease; AF, atrial fibrillation; CAD, coronary artery disease; HF, heart failure; AS, any stroke; AIS, any ischemic stroke.

**Figure S1. Scatter plots of MR tests from eGFR on cardiovascular diseases.**

(A). eGFR on atrial fibrillation; (B). eGFR on coronary artery disease; (C). eGFR on heart failure; (D). eGFR on any stroke; (E). eGFR on any ischemic stroke.

Abbreviations: MR, Mendelian randomization; eGFR, estimated glomerular filtration rate.


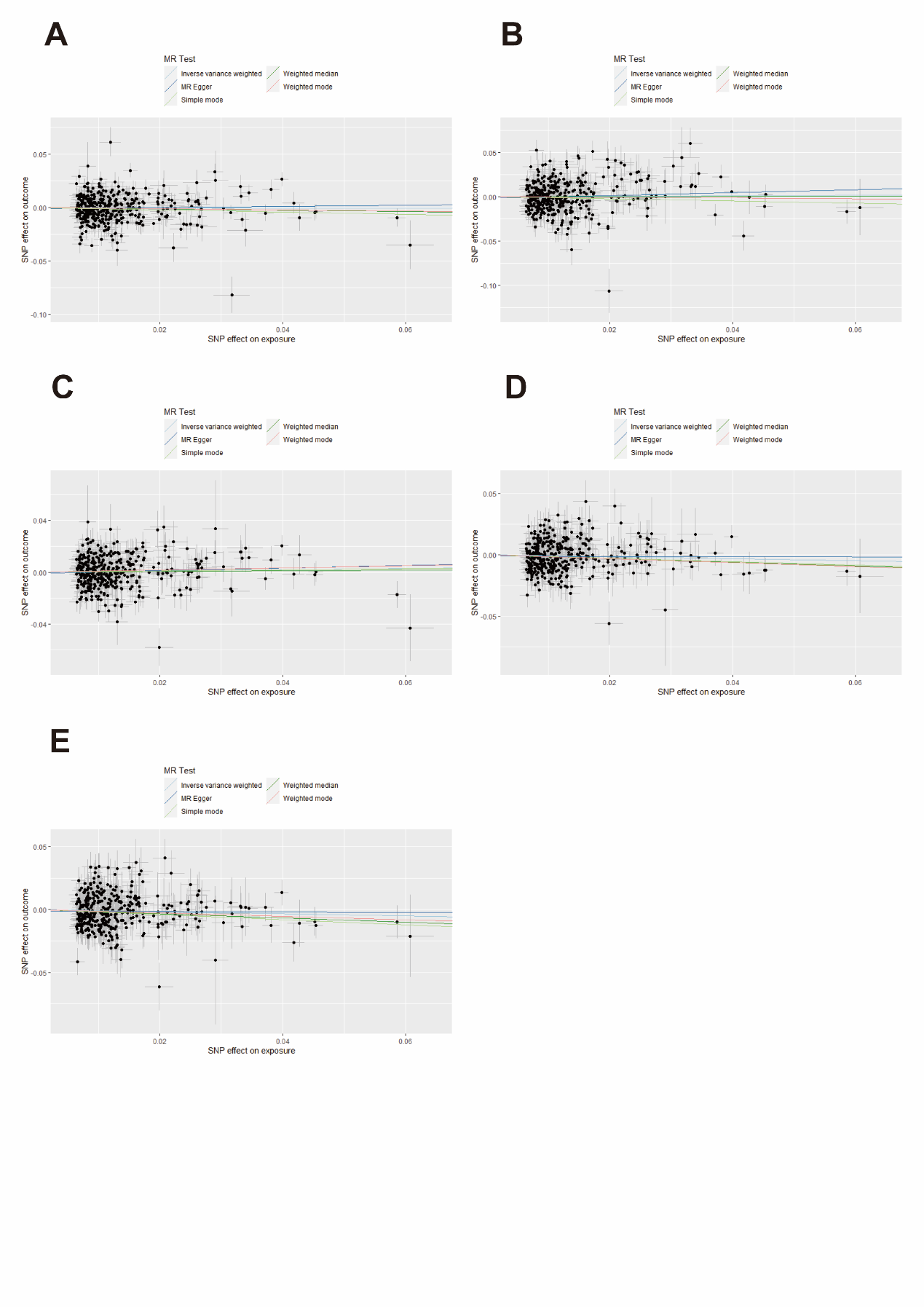


**Figure S2. Scatter plots of MR tests from CKD on cardiovascular diseases.**

(A). CKD on atrial fibrillation; (B). CKD on coronary artery disease; (C). CKD on heart failure; (D). CKD on any stroke; (E). CKD on any ischemic stroke.

Abbreviations: MR, Mendelian randomization; CKD, chronic kidney disease.


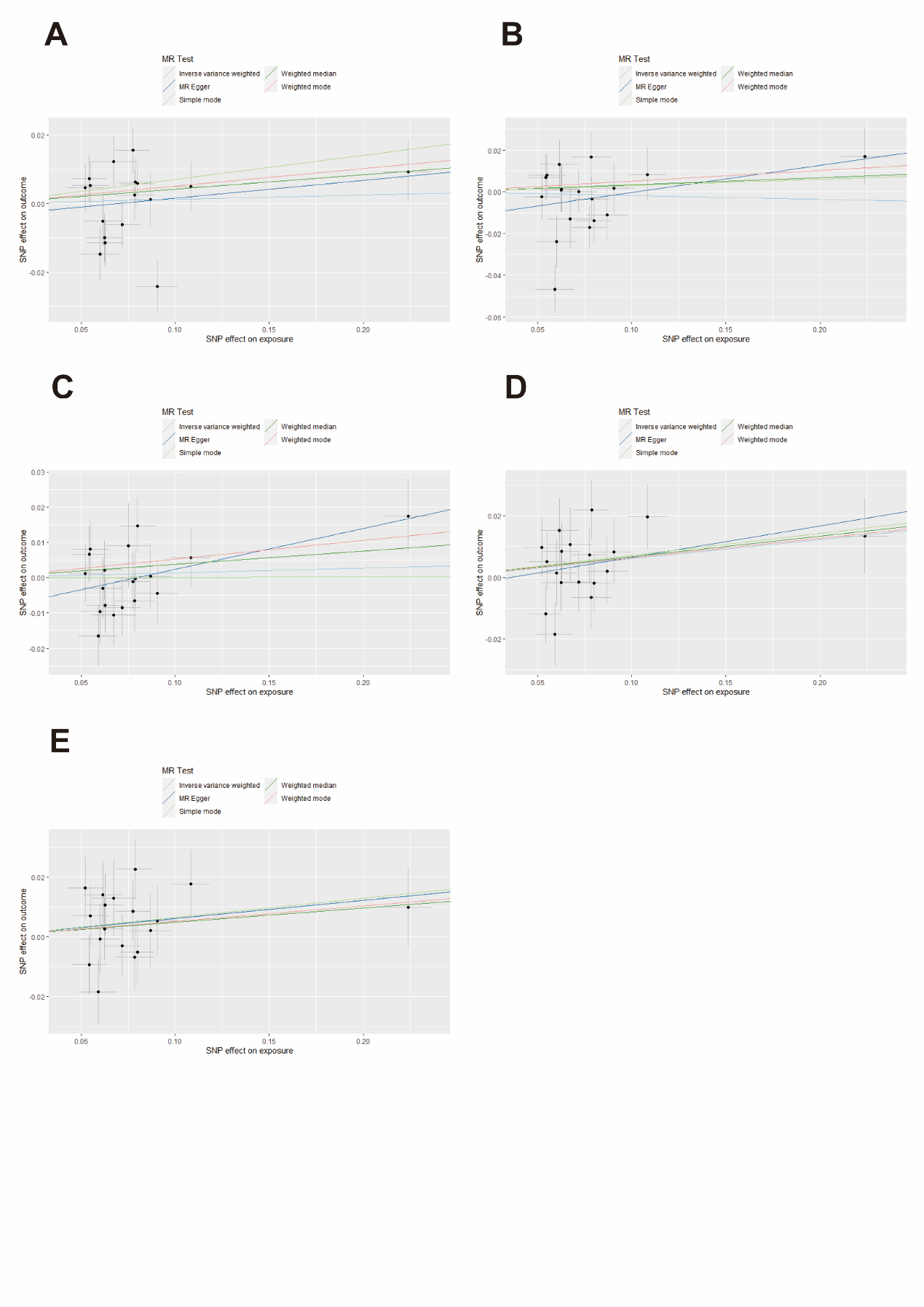


**Figure S3. Scatter plots of MR tests from atrial fibrillation on kidney function.**

(A). atrial fibrillation on eGFR; (B) atrial fibrillation on CKD.

Abbreviations: MR, Mendelian randomization; eGFR, estimated glomerular filtration rate; CKD, chronic kidney disease.


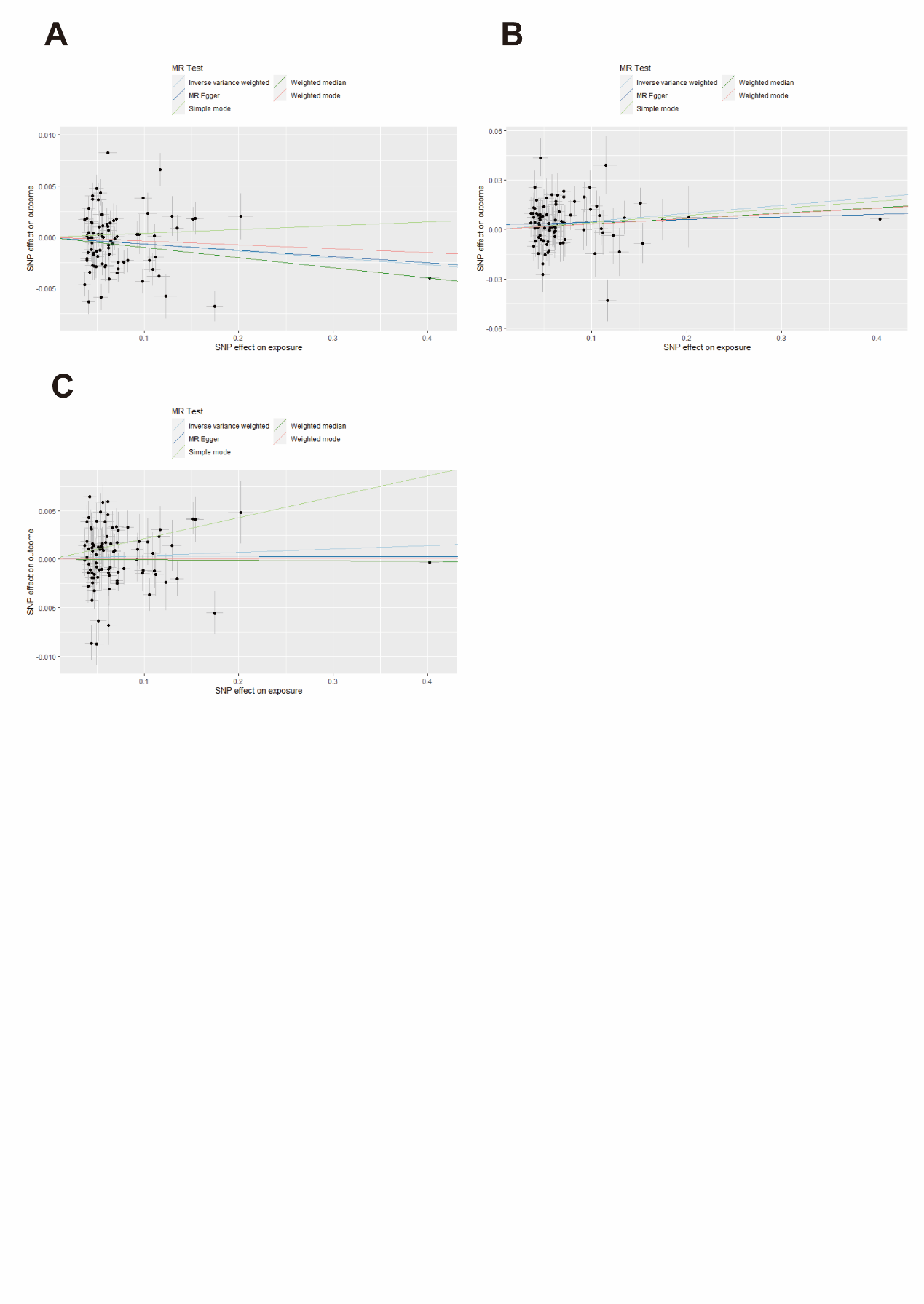


**Figure S4. Scatter plots of MR tests from coronary artery disease on kidney function.**

(A). coronary artery disease on eGFR; (B) coronary artery disease on CKD.

Abbreviations: MR, Mendelian randomization; eGFR, estimated glomerular filtration rate; CKD, chronic kidney disease.


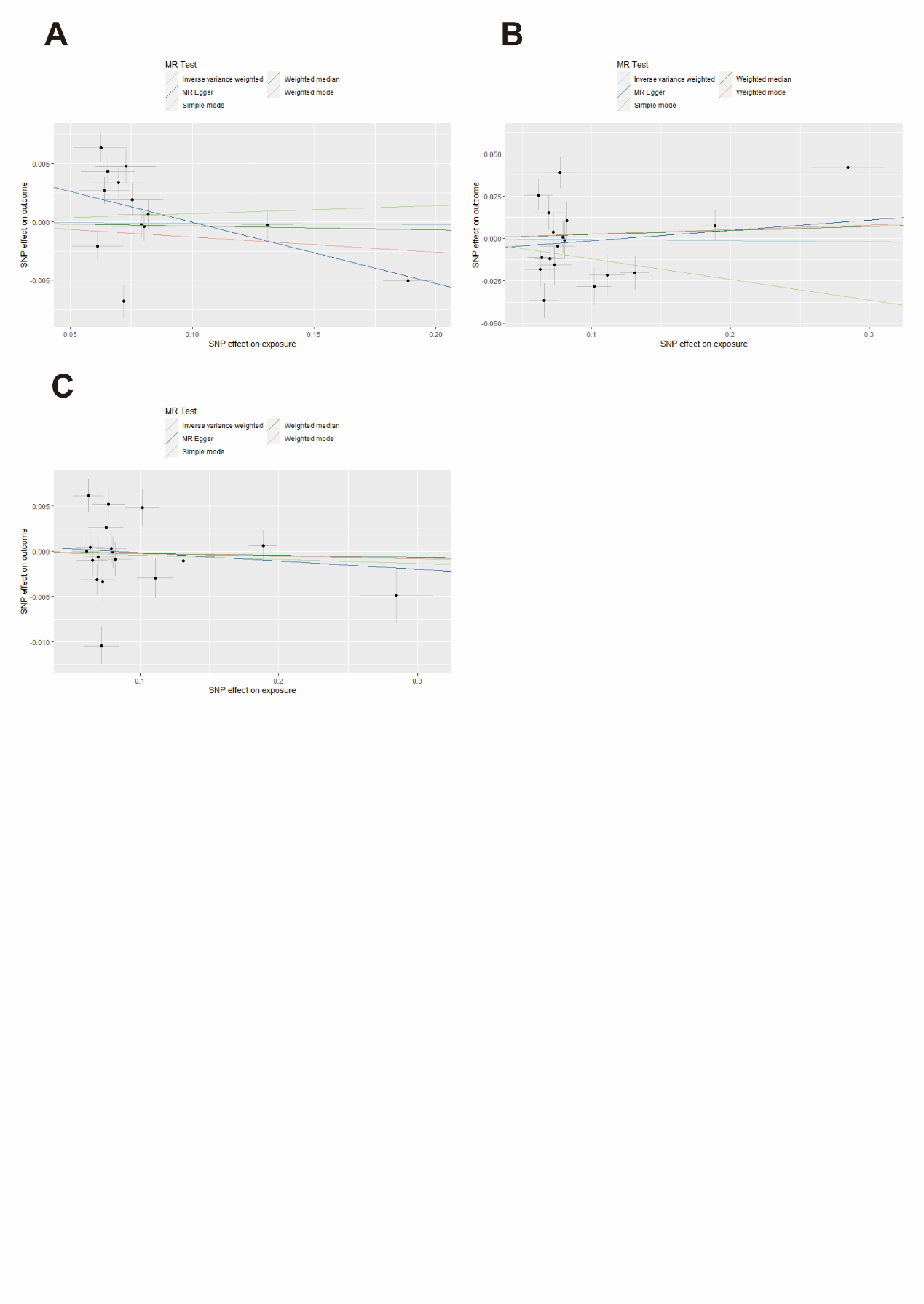


**Figure S5. Scatter plots of MR tests from heart failure on kidney function.**

(A). heart failure on eGFR; (B). heart failure on CKD.

Abbreviations: MR, Mendelian randomization; eGFR, estimated glomerular filtration rate; CKD, chronic kidney disease.


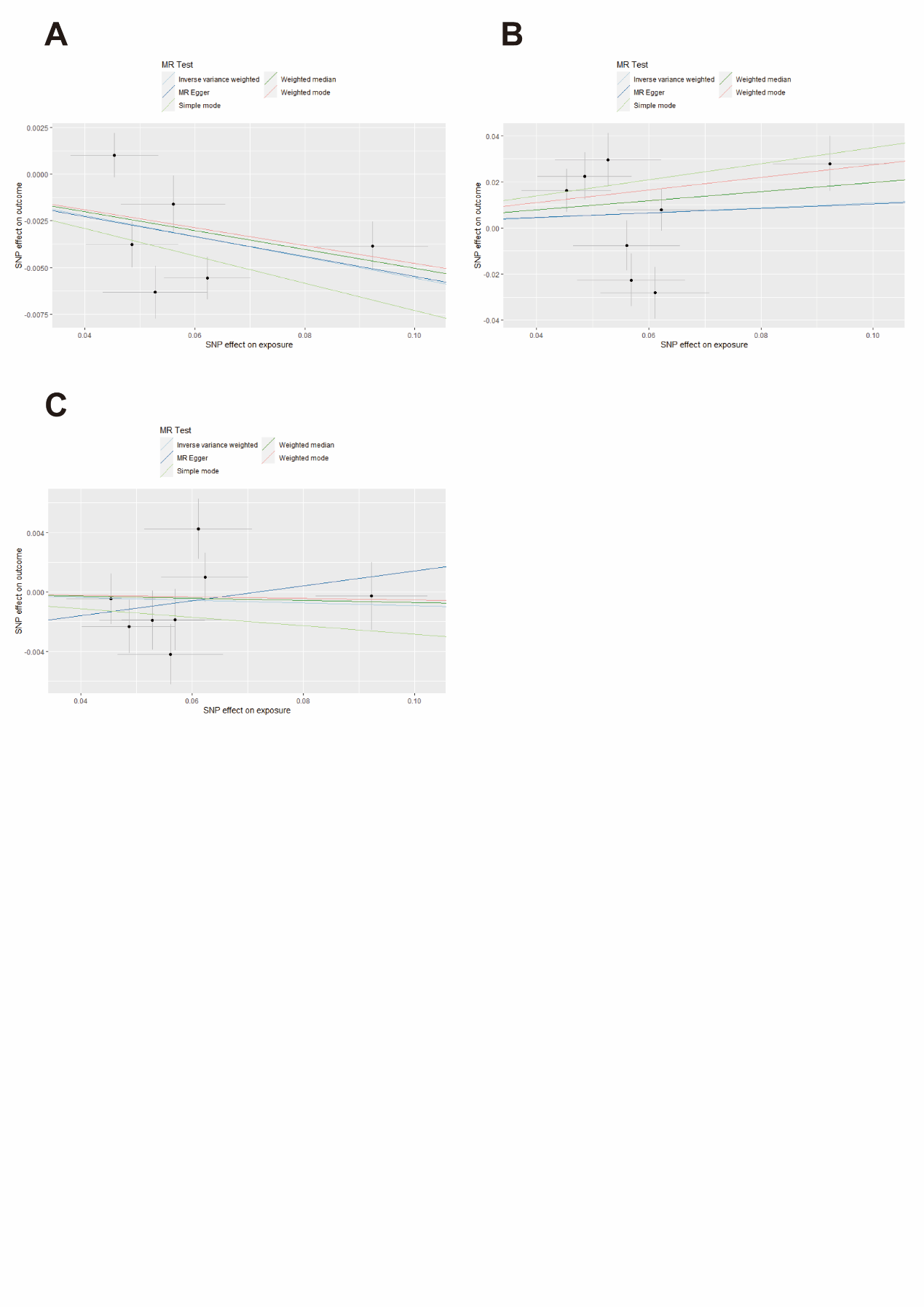


**Figure S6. Scatter plots of MR tests from any stroke on kidney function.**

(A). any stroke on eGFR; (B). any stroke on CKD.

Abbreviations: MR, Mendelian randomization; eGFR, estimated glomerular filtration rate; CKD, chronic kidney disease.
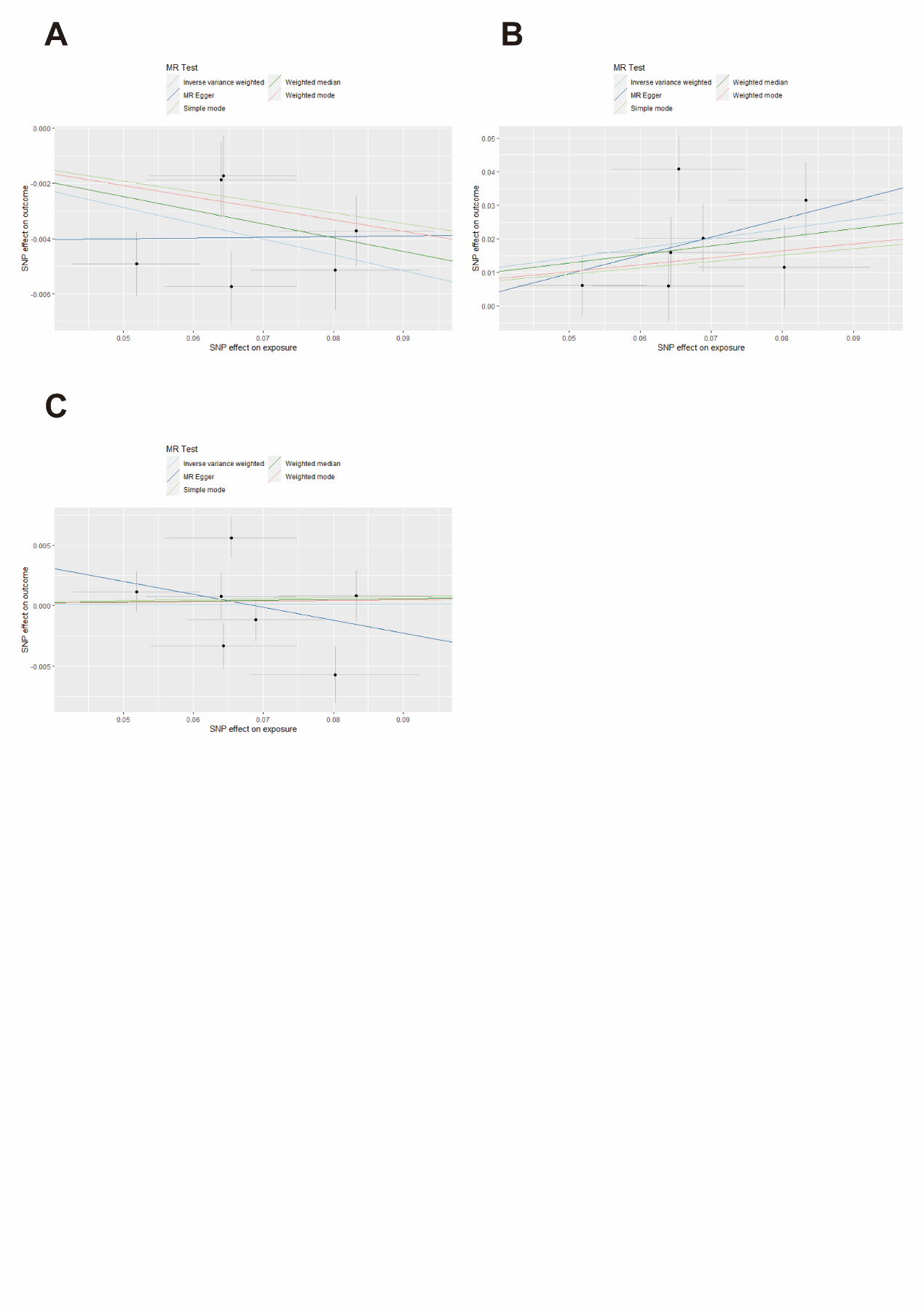


**Figure S7. Scatter plots of MR tests from any ischemic stroke on kidney function.**

(A). any ischemic stroke on eGFR; (B) any ischemic stroke on CKD.

Abbreviations: MR, Mendelian randomization; eGFR, estimated glomerular filtration rate; CKD, chronic kidney disease.


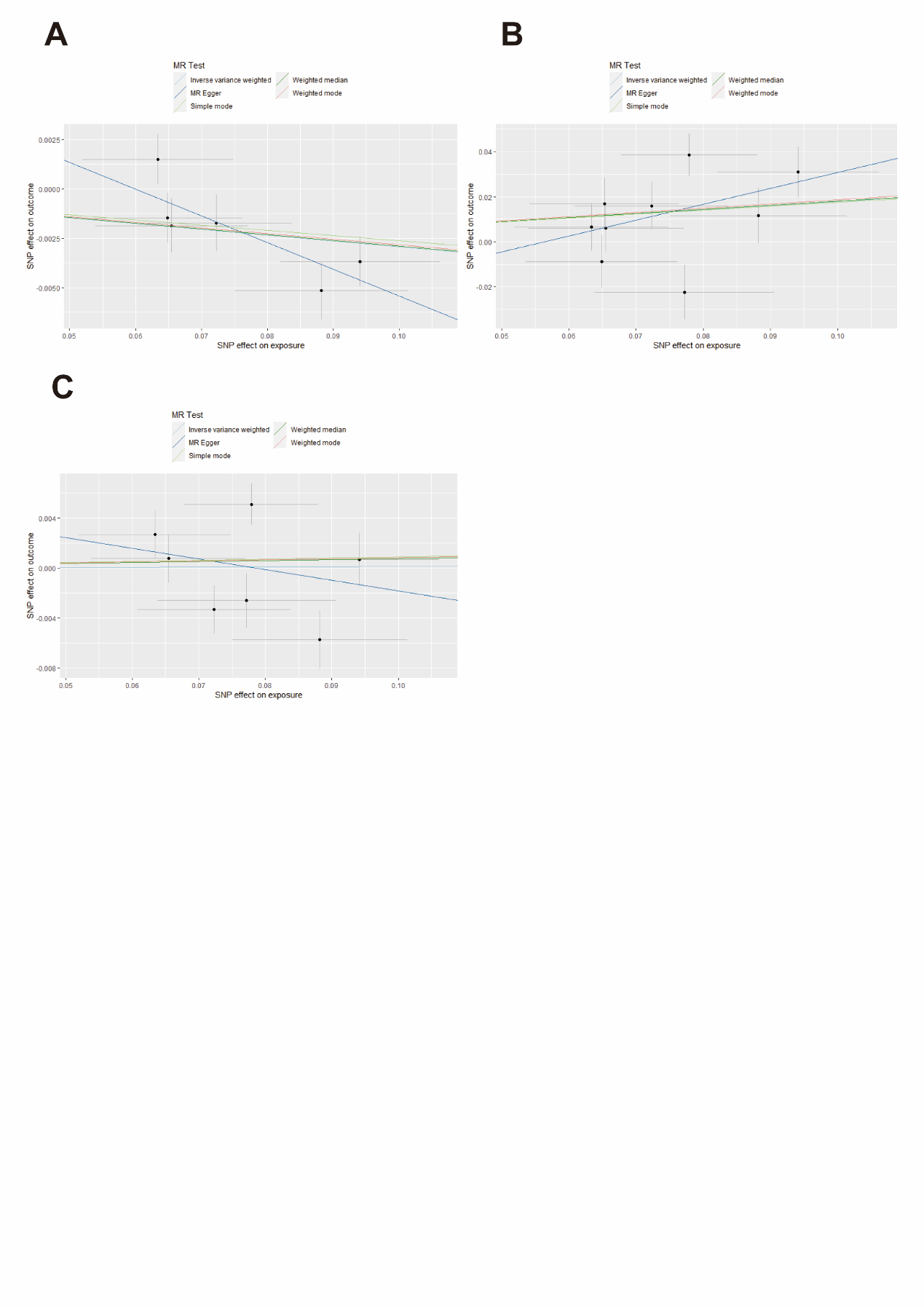


**Figure S8. Leave-one-out plots of MR tests from eGFR on cardiovascular diseases.**

(A). eGFR on atrial fibrillation; (B). eGFR on coronary artery disease; (C). eGFR on heart failure; (D). eGFR on any stroke; (E). eGFR on any ischemic stroke.

Abbreviations: MR, Mendelian randomization; eGFR, estimated glomerular filtration rate.


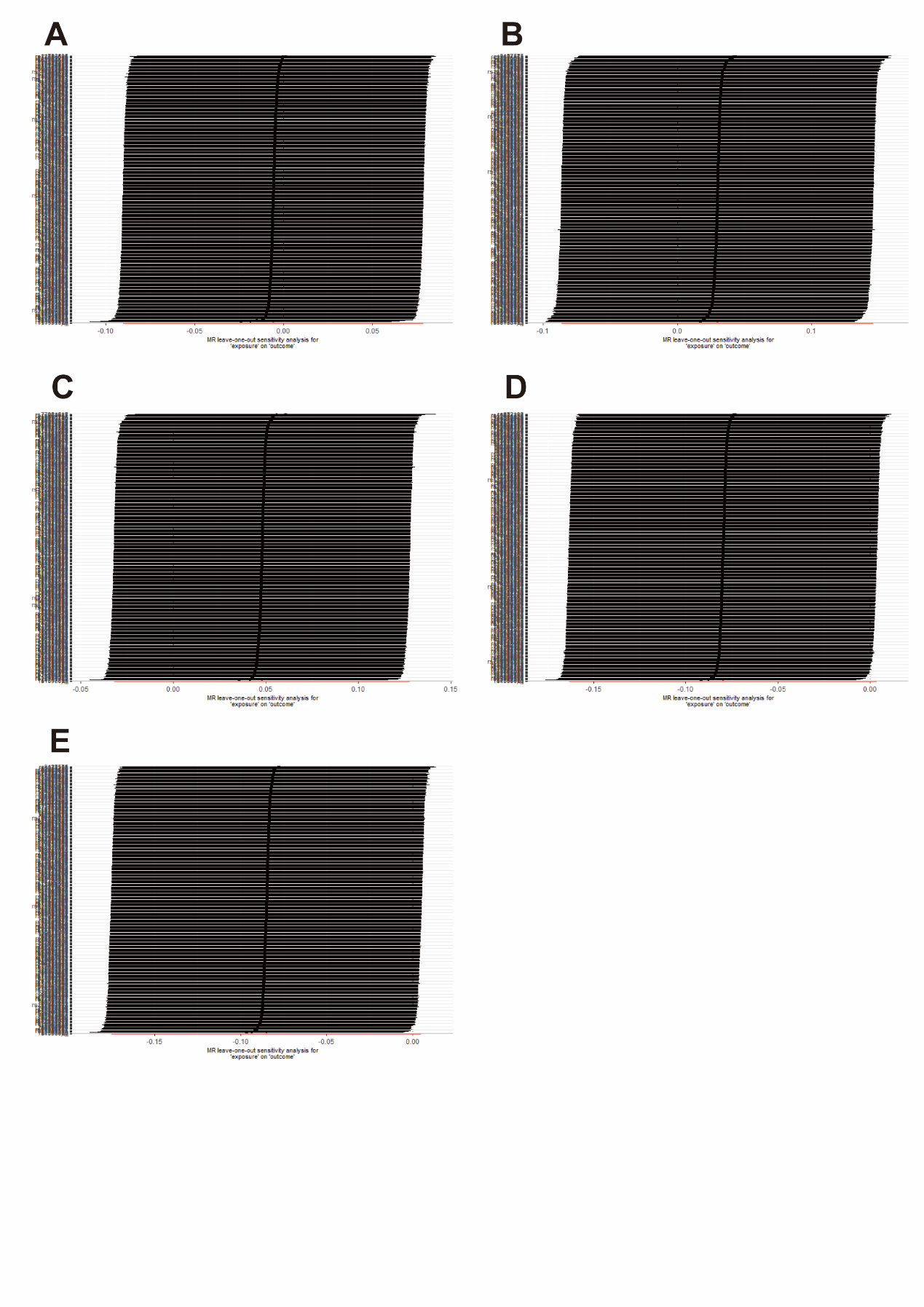


**Figure S9. Leave-one-out plots of MR tests from CKD on cardiovascular diseases.**

(A). CKD on atrial fibrillation; (B). CKD on coronary artery disease; (C). CKD on heart failure; (D). CKD on any stroke; (E). CKD on any ischemic stroke.

Abbreviations: MR, Mendelian randomization; CKD, chronic kidney disease.


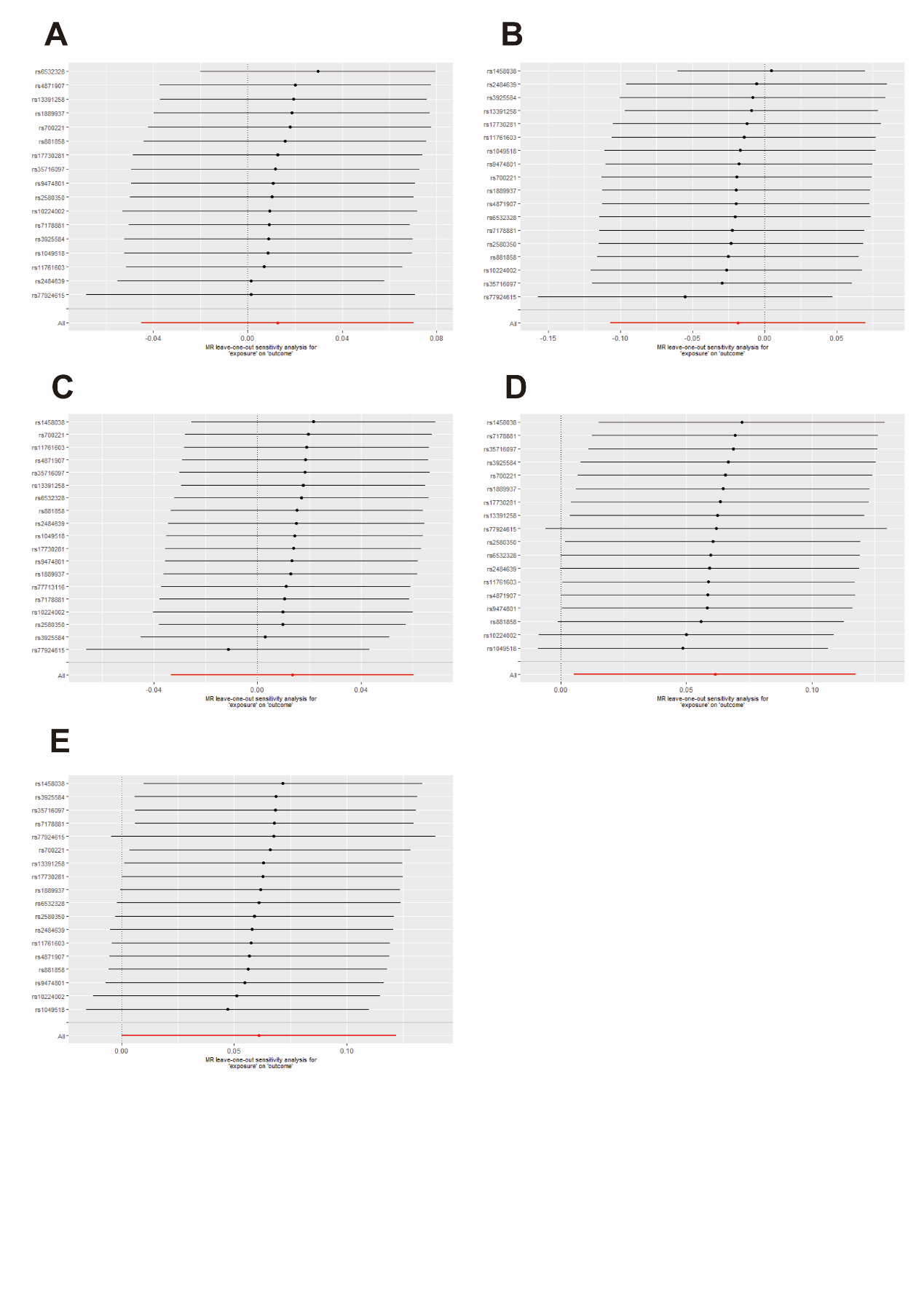


**Figure S10. Leave-one-out plots of MR tests from atrial fibrillation on kidney function.**

(A). atrial fibrillation on eGFR; (B) atrial fibrillation on CKD.

Abbreviations: MR, Mendelian randomization; eGFR, estimated glomerular filtration rate; CKD, chronic kidney disease.


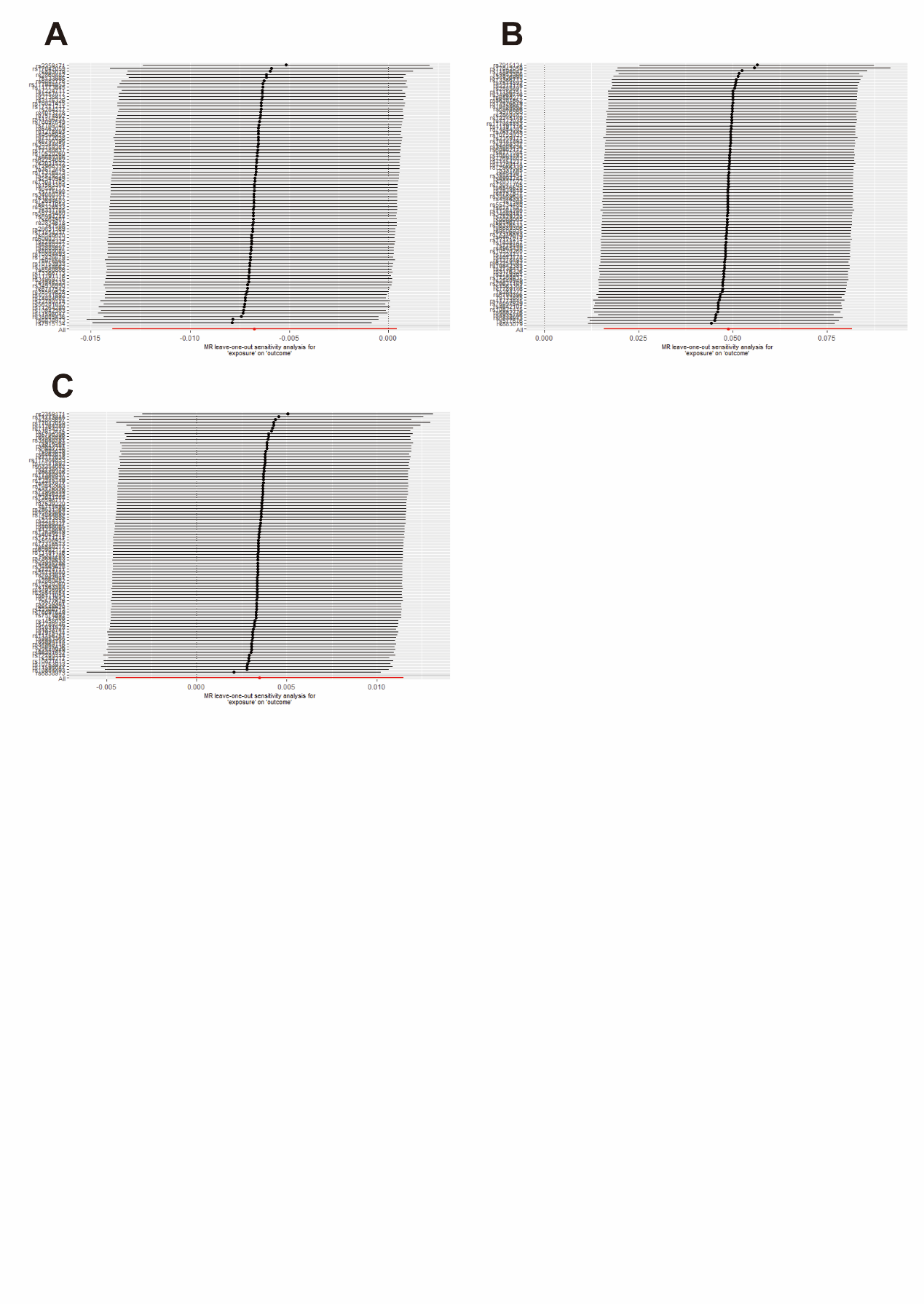


**Figure S11. Leave-one-out plots of MR tests from coronary artery disease on kidney function.**

(A). coronary artery disease on eGFR; (B) coronary artery disease on CKD.

Abbreviations: MR, Mendelian randomization; eGFR, estimated glomerular filtration rate; CKD, chronic kidney disease.


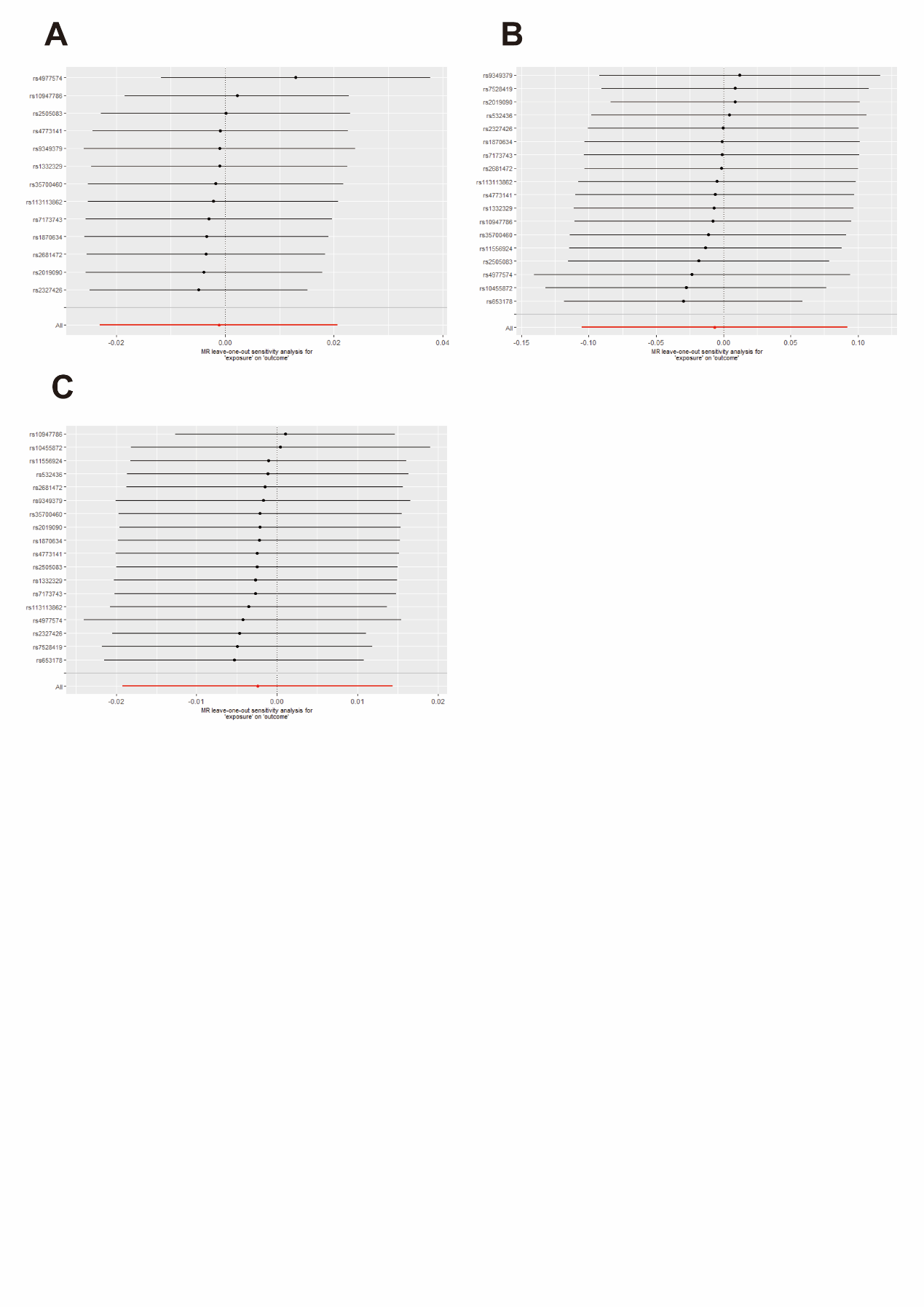


**Figure S12. Leave-one-out plots of MR tests from heart failure on kidney function.**

(A). heart failure on eGFR; (B). heart failure on CKD.

Abbreviations: MR, Mendelian randomization; eGFR, estimated glomerular filtration rate; CKD, chronic kidney disease.


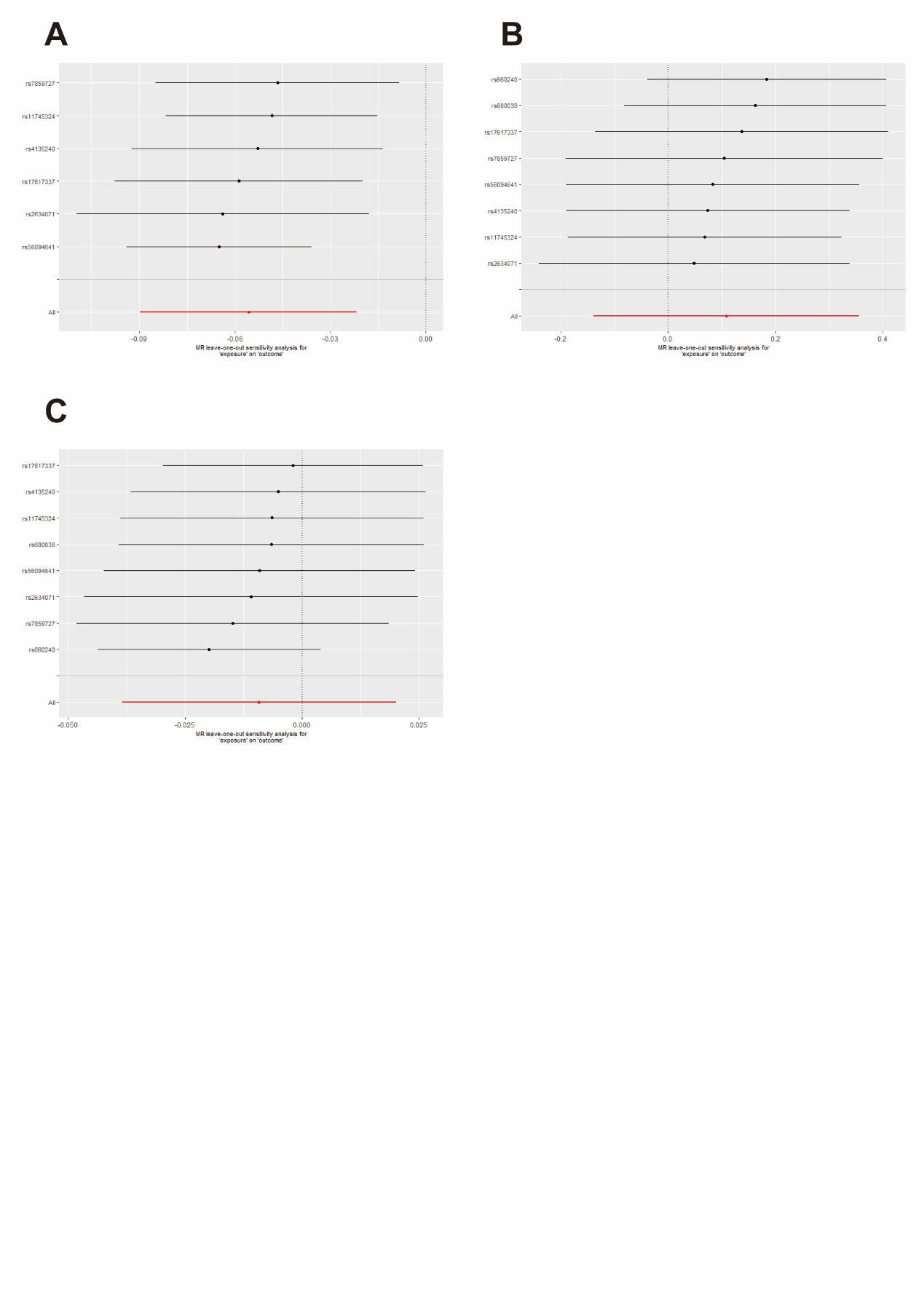


**Figure S13. Leave-one-out plots of MR tests from any stroke on kidney function.**

(A). any stroke on eGFR; (B). any stroke on CKD.

Abbreviations: MR, Mendelian randomization; eGFR, estimated glomerular filtration rate; CKD, chronic kidney disease.


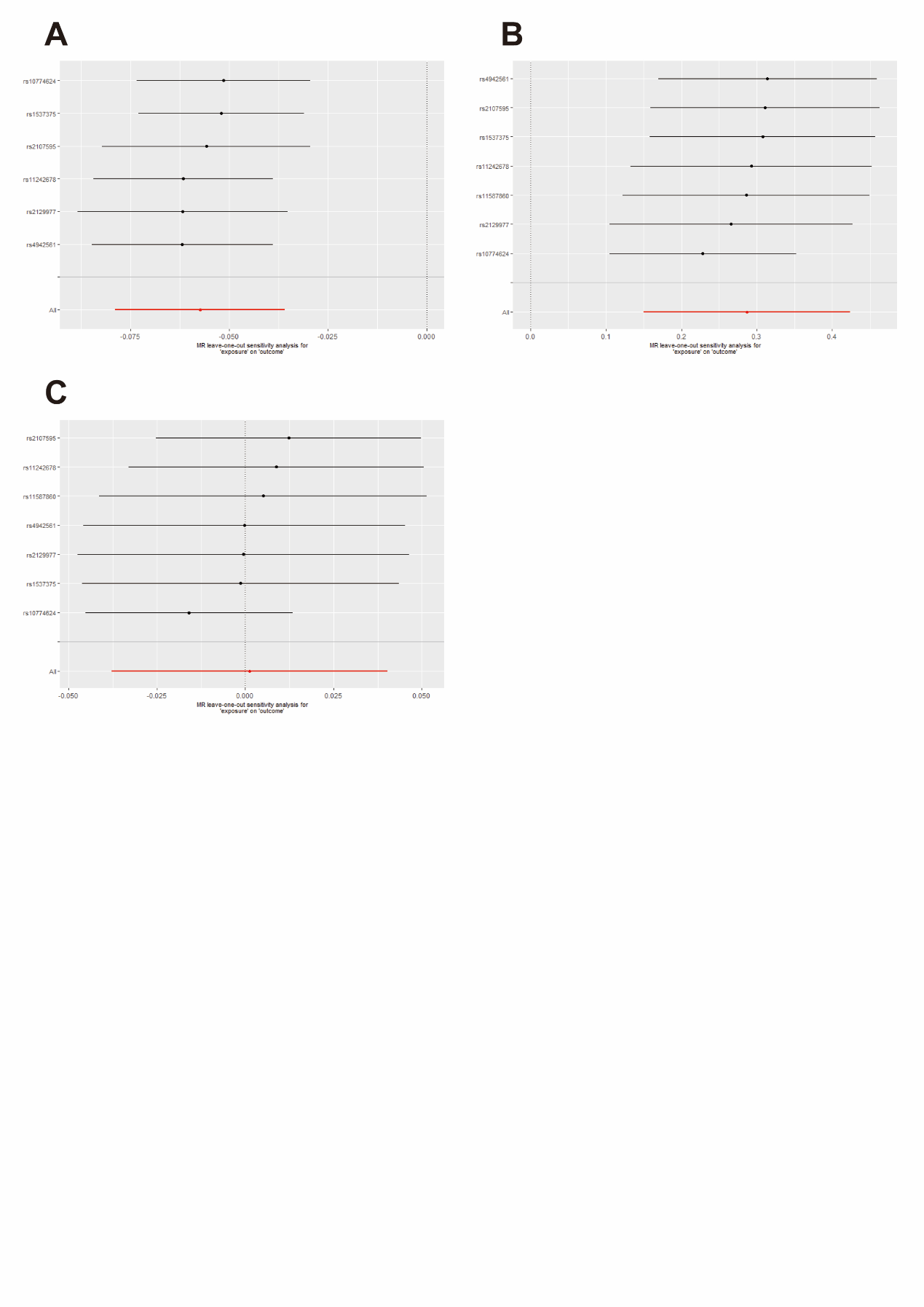


**Figure S14. Leave-one-out plots of MR tests from any ischemic stroke on kidney function.**

(A). any ischemic stroke on eGFR; (B) any ischemic stroke on CKD.

Abbreviations: MR, Mendelian randomization; eGFR, estimated glomerular filtration rate; CKD, chronic kidney disease.


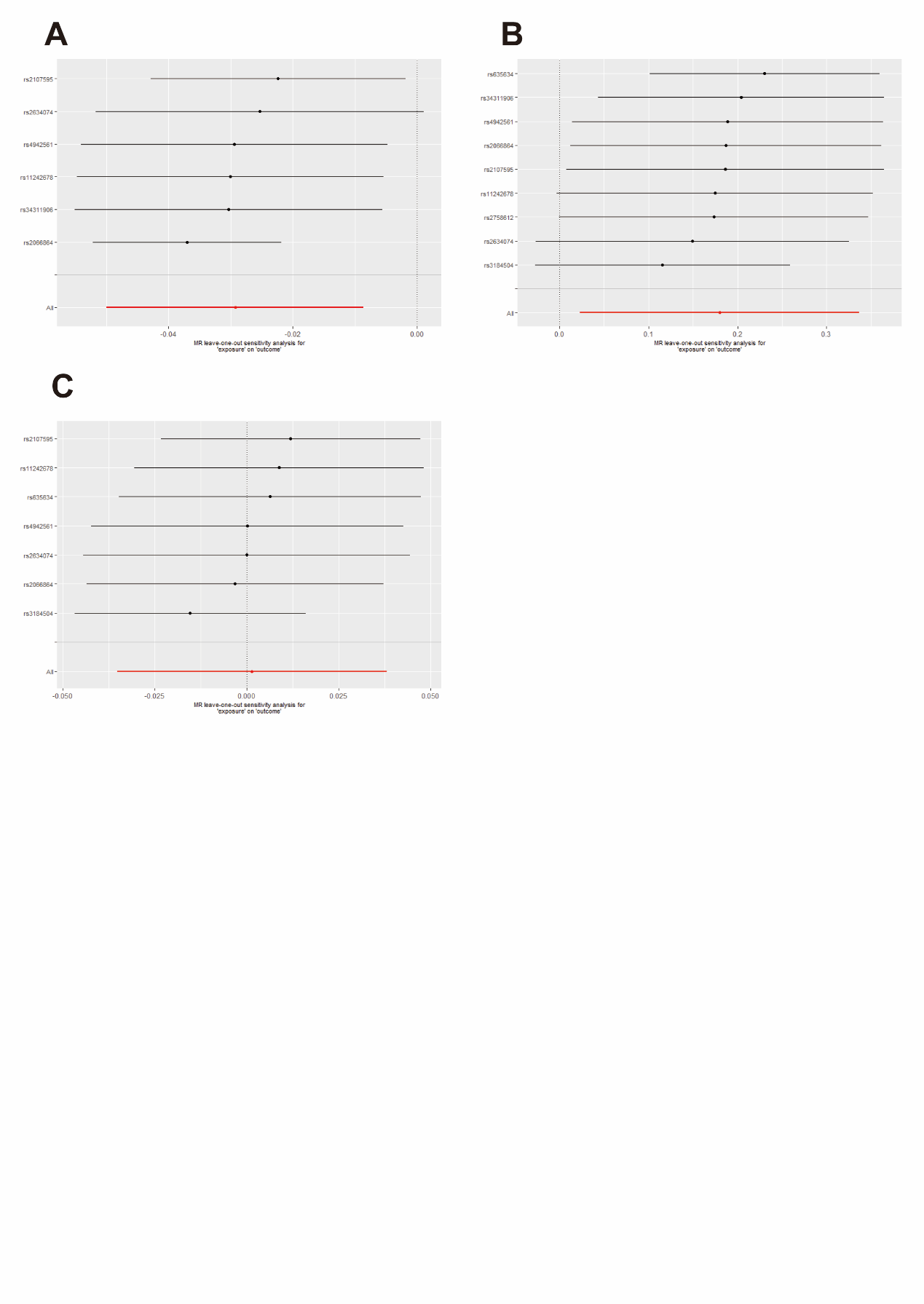


**Figure S15. Funnel plots of MR tests from eGFR on cardiovascular diseases.**

(A). eGFR on atrial fibrillation; (B). eGFR on coronary artery disease; (C). eGFR on heart failure; (D). eGFR on any stroke; (E). eGFR on any ischemic stroke.

Abbreviations: MR, Mendelian randomization; eGFR, estimated glomerular filtration rate.


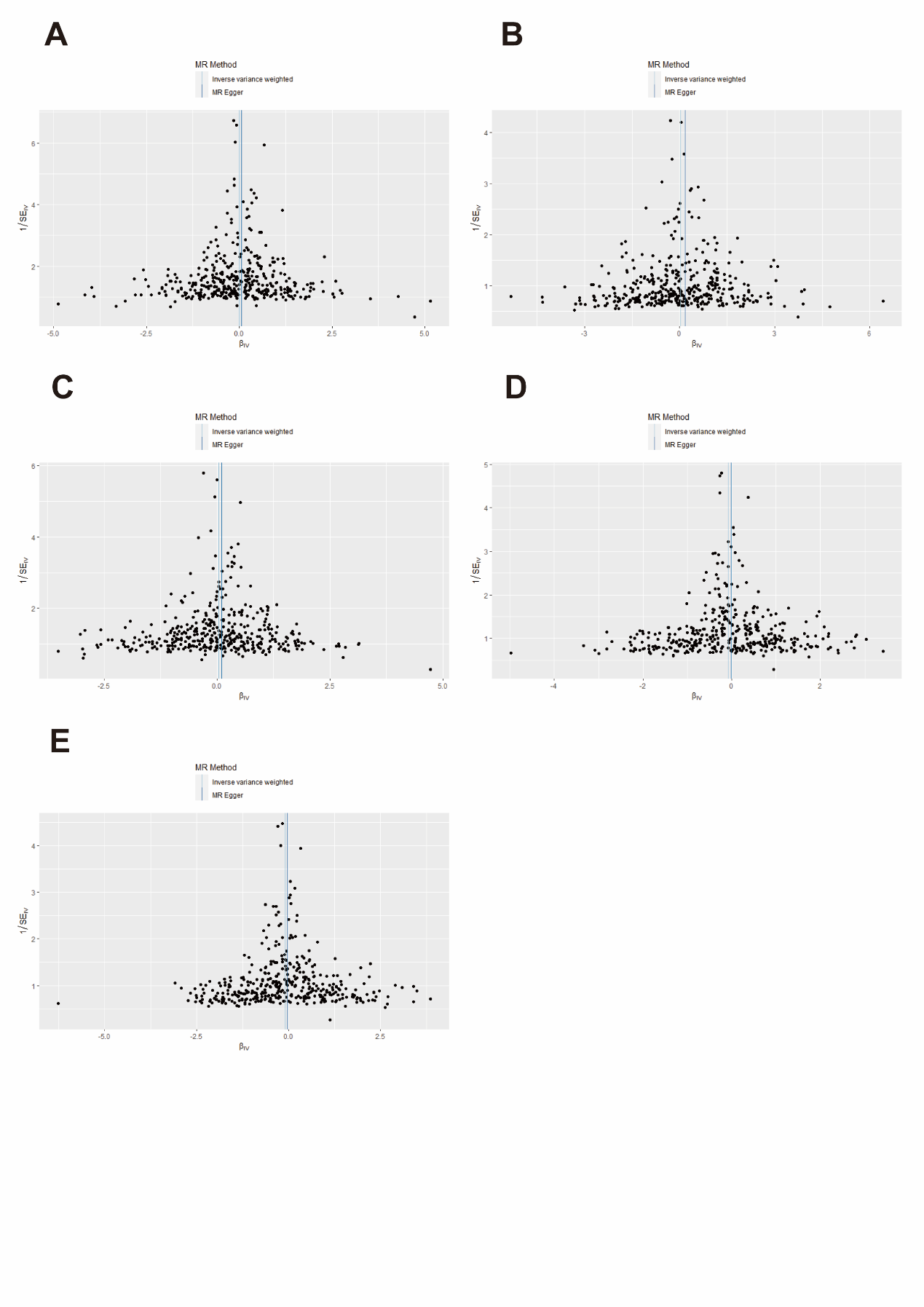


**Figure S16. Funnel plots of MR tests from CKD on cardiovascular diseases.**

(A). CKD on atrial fibrillation; (B). CKD on coronary artery disease; (C). CKD on heart failure; (D). CKD on any stroke; (E). CKD on any ischemic stroke.

Abbreviations: MR, Mendelian randomization; CKD, chronic kidney disease.


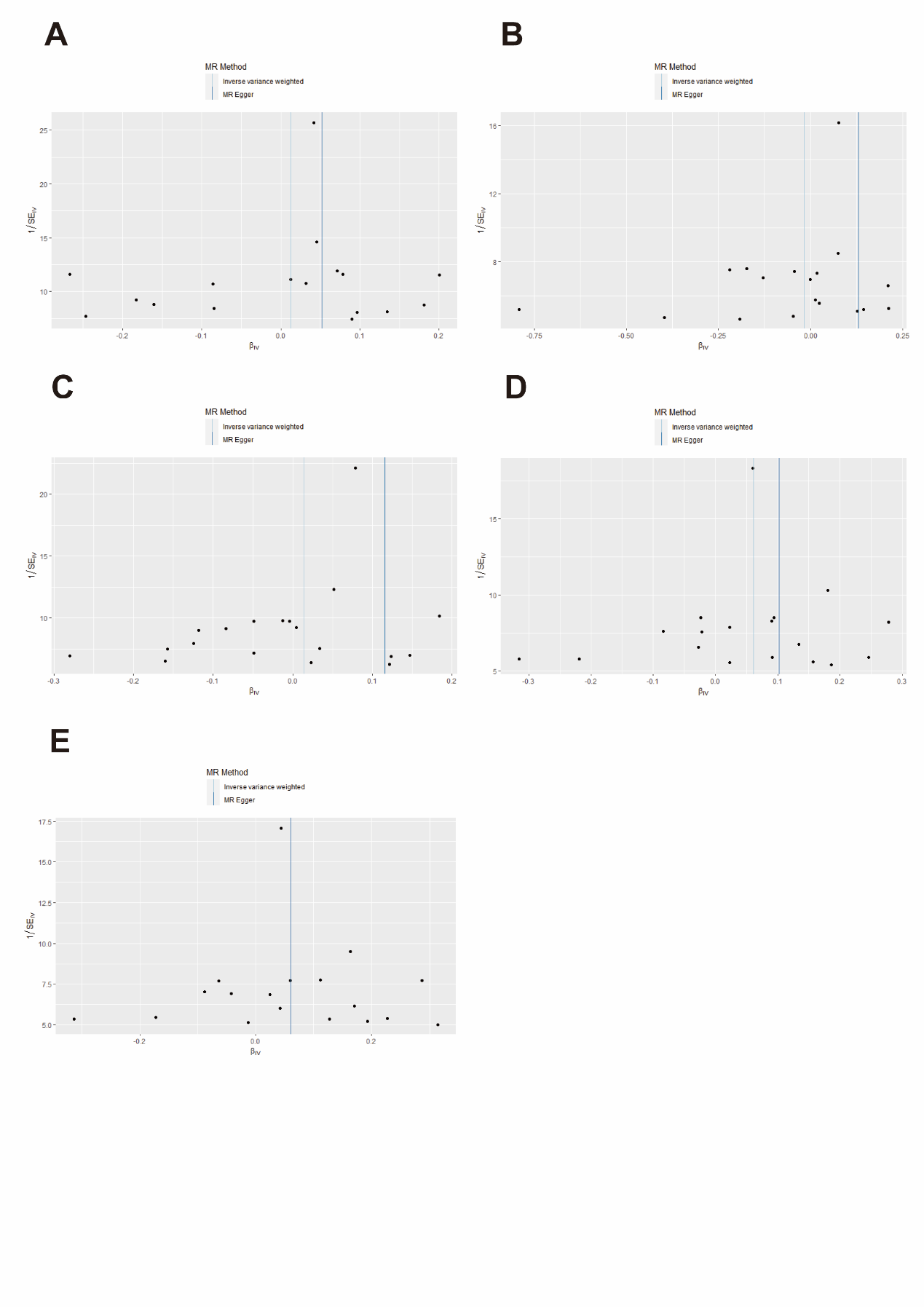


**Figure S17. Funnel plots of MR tests from atrial fibrillation on kidney function.**

(A). atrial fibrillation on eGFR; (B) atrial fibrillation on CKD.

Abbreviations: MR, Mendelian randomization; eGFR, estimated glomerular filtration rate; CKD, chronic kidney disease.


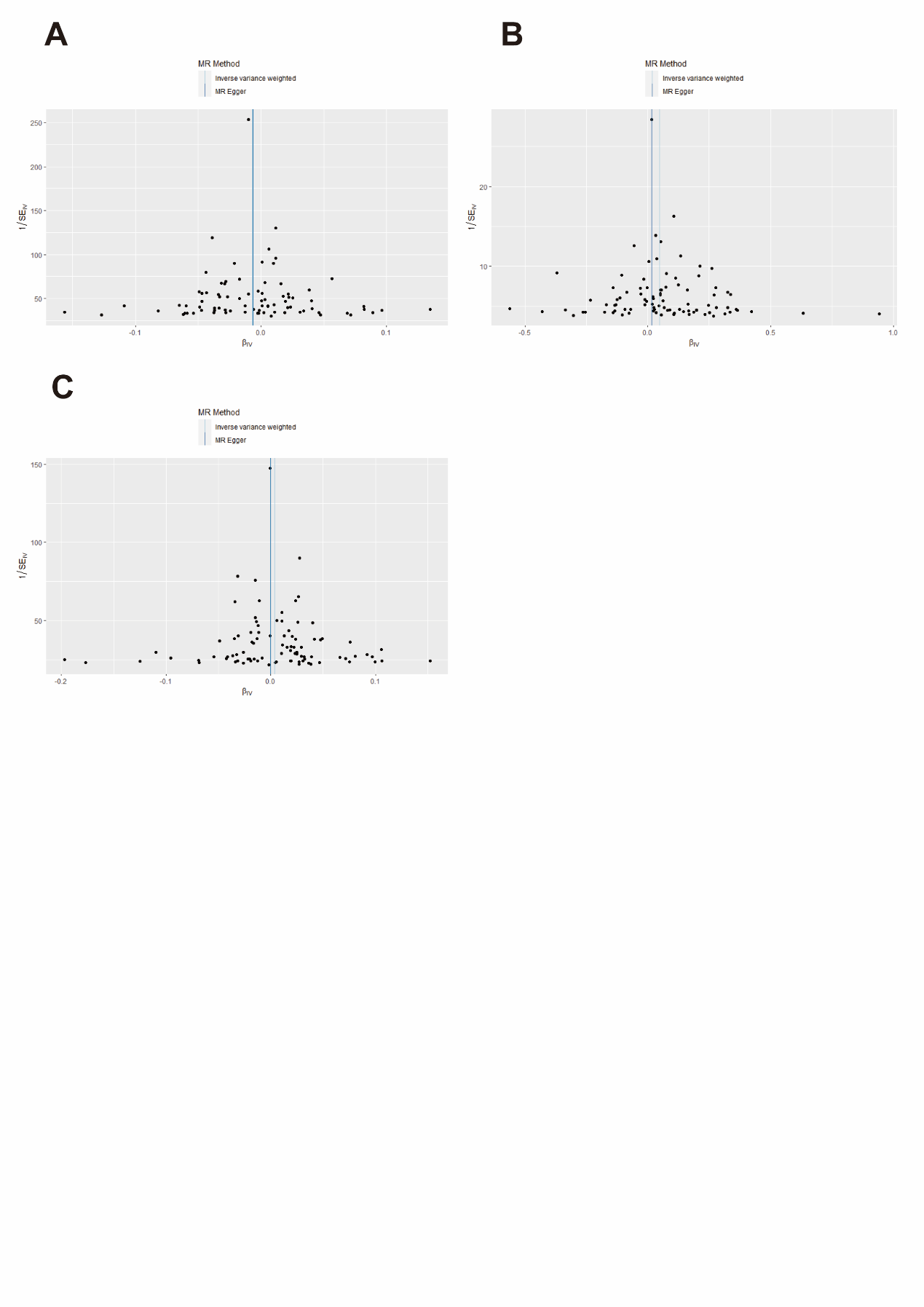


**Figure S18. Funnel plots of MR tests from coronary artery disease on kidney function.**

(A). coronary artery disease on eGFR; (B) coronary artery disease on CKD.

Abbreviations: MR, Mendelian randomization; eGFR, estimated glomerular filtration rate; CKD, chronic kidney disease.


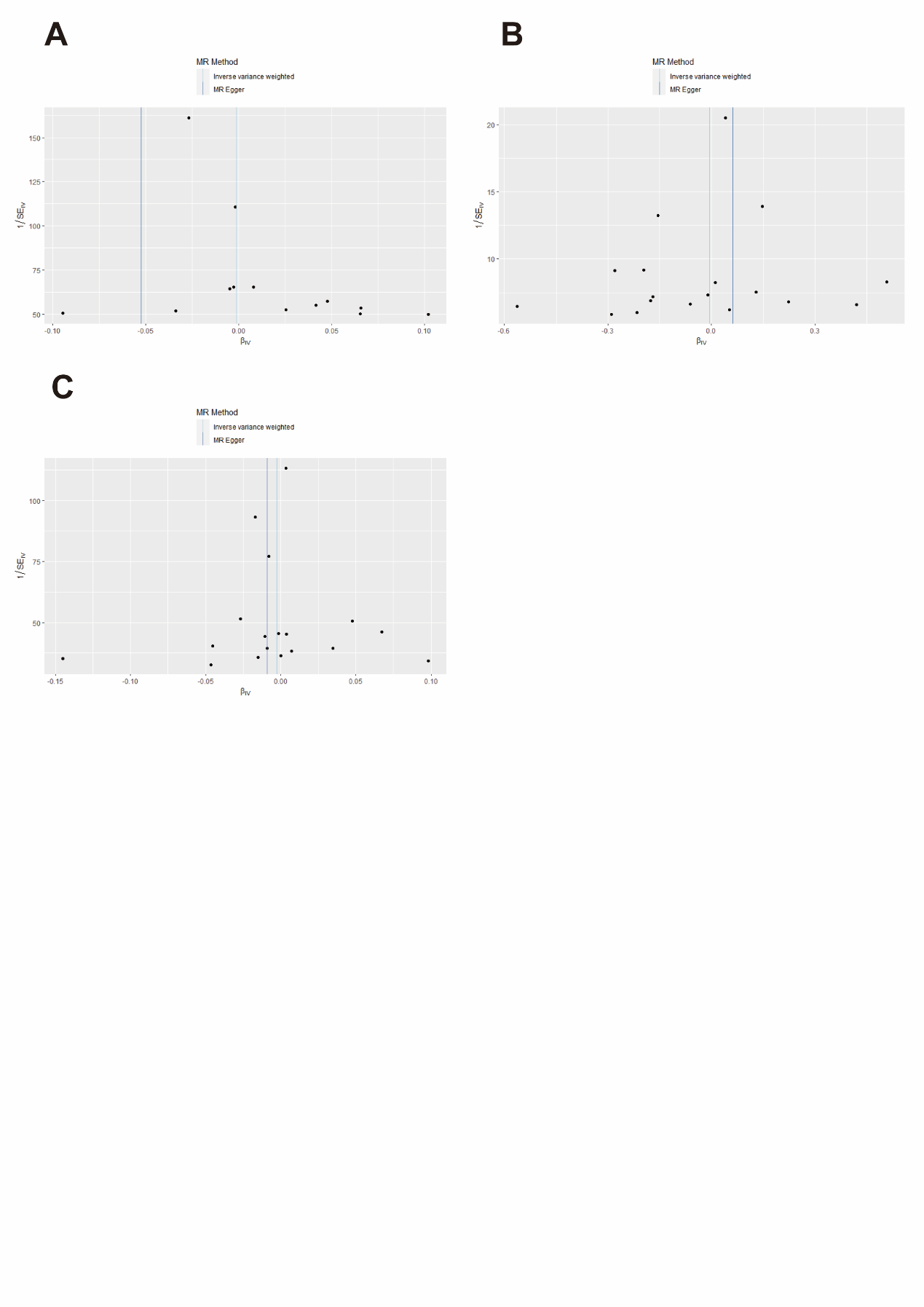


**Figure S19. Funnel plots of MR tests from heart failure on kidney function.**

(A). heart failure on eGFR; (B). heart failure on CKD.

Abbreviations: MR, Mendelian randomization; eGFR, estimated glomerular filtration rate; CKD, chronic kidney disease.


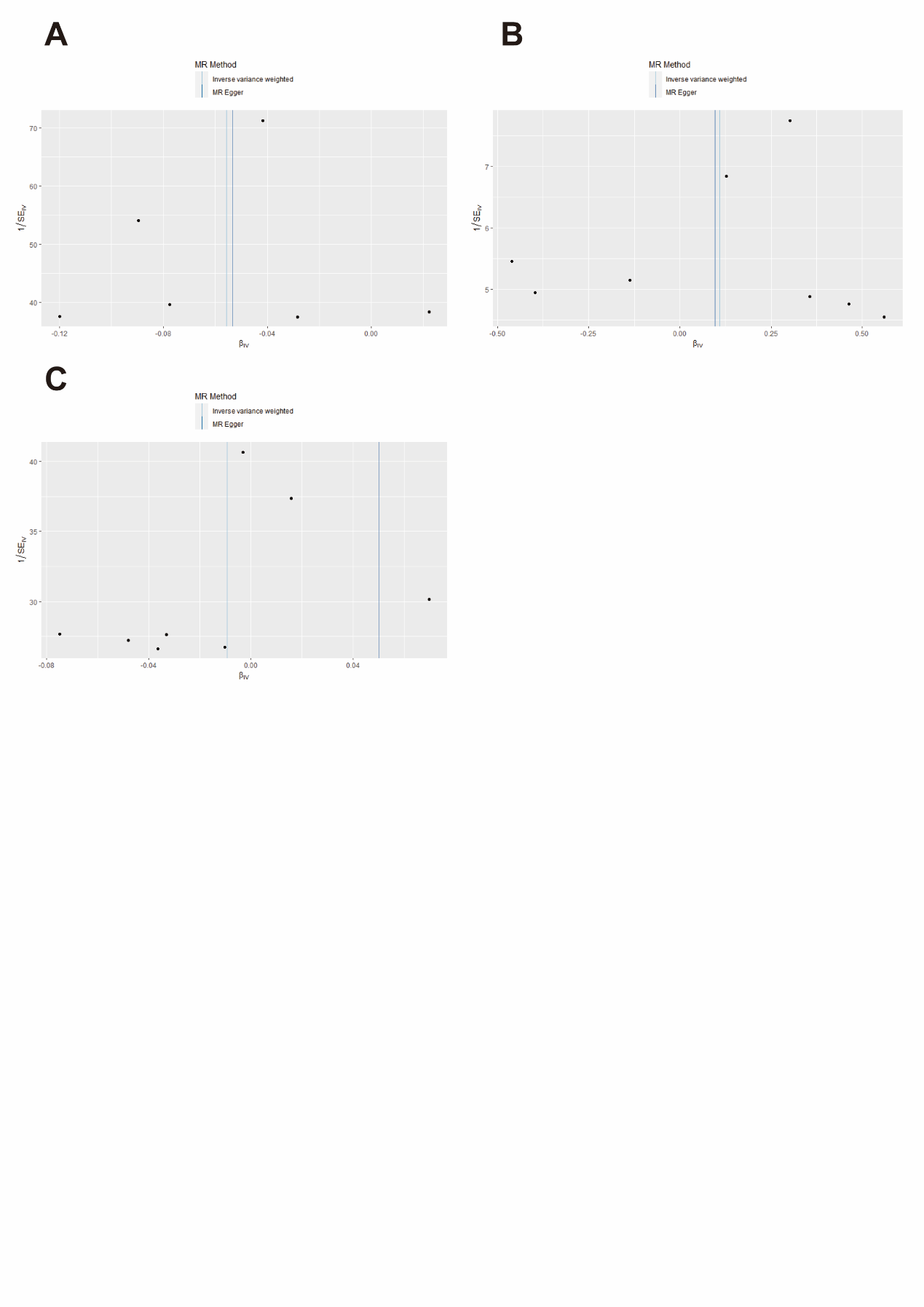


**Figure S20. Funnel plots of MR tests from any stroke on kidney function.**

(A). any stroke on eGFR; (B). any stroke on CKD.

Abbreviations: MR, Mendelian randomization; eGFR, estimated glomerular filtration rate; CKD, chronic kidney disease.


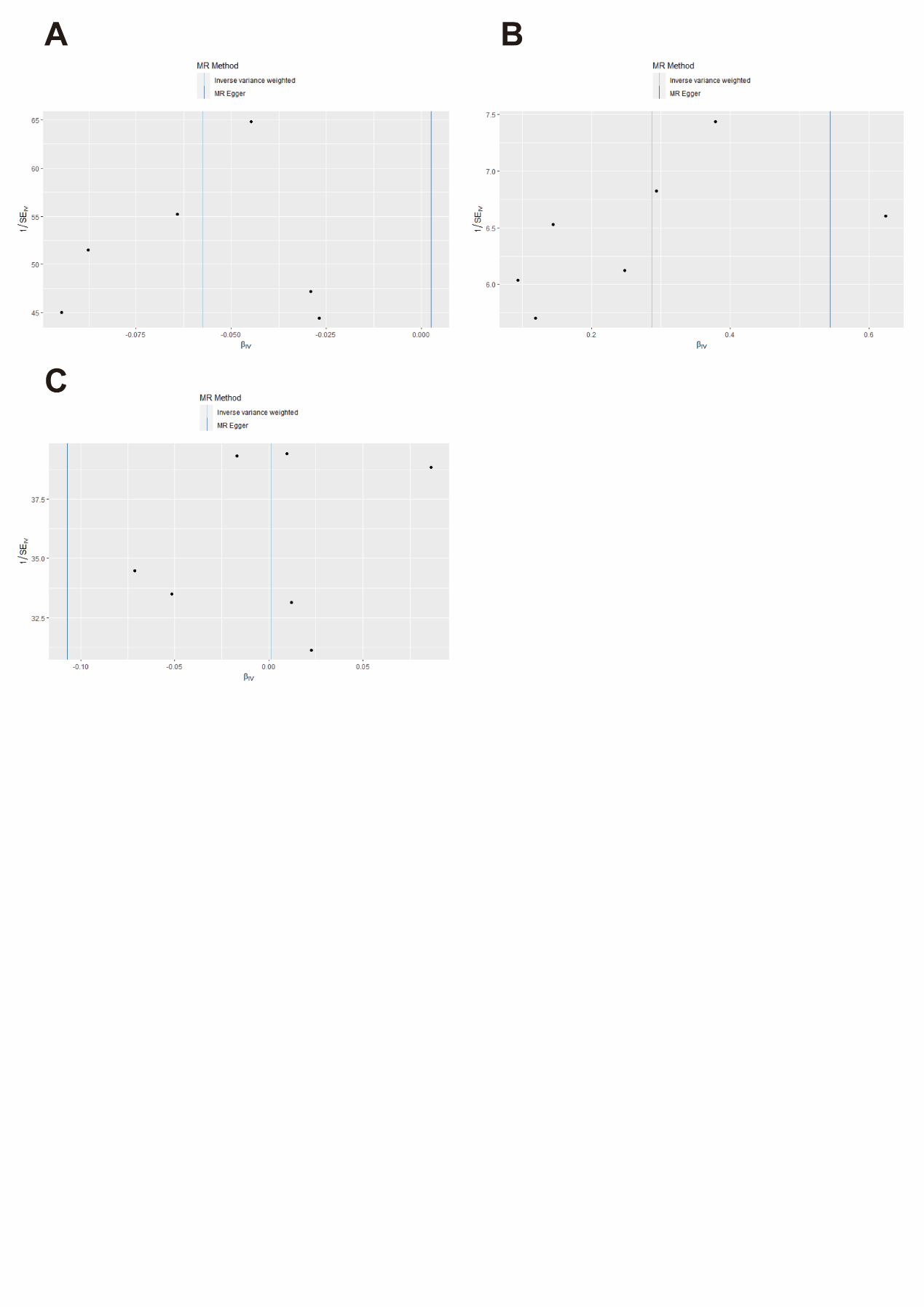


**Figure S21. Funnel plots of MR tests from any ischemic stroke on kidney function.**

(A). any ischemic stroke on eGFR; (B) any ischemic stroke on CKD.

Abbreviations: MR, Mendelian randomization; eGFR, estimated glomerular filtration rate; CKD, chronic kidney disease.


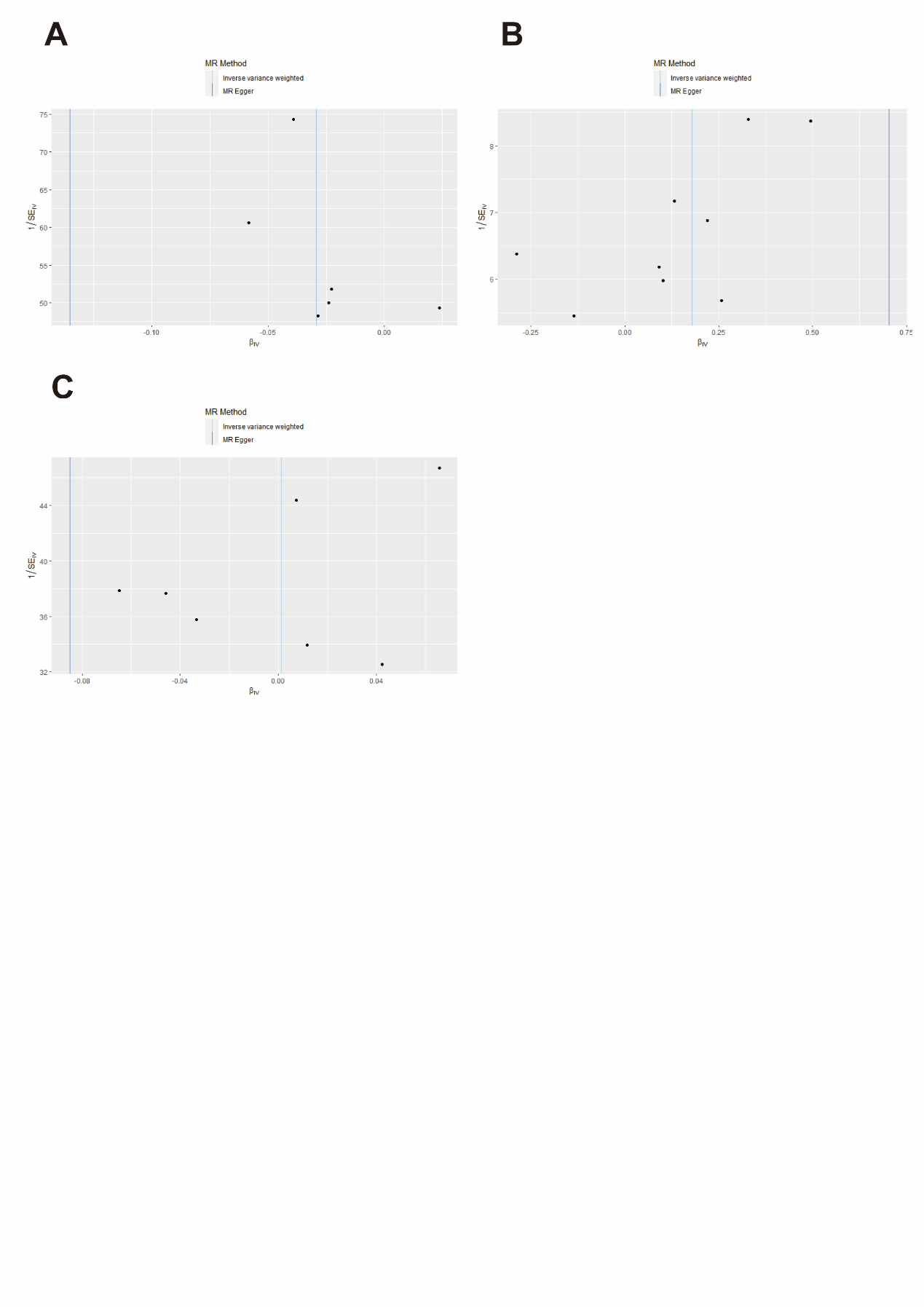


**Acknowledgments**

We wish to acknowledge the participants and investigators of the eICU-CRD, the UK Biobank, the CKDGen Consortium, the MVP, the PAGE, the SUMMIT consortia, the HUNT, the deCODE, the MGI, the DiscovEHR, the AFGen, the CARDIoGRAMplusC4D, the HERMES and the METASTROKE.

**Publication, author and funding related to the eICU-CRD**

Pollard TJ, Johnson AEW, Raffa JD, Celi LA, Mark RG, Badawi O. The eICU Collaborative Research Database, a freely available multi-center database for critical care research. *Sci Data*. 2018;5:180178. doi:10.1038/sdata.2018.178.

This work was supported by grants NIH-R01-EB017205, NIH-R01-EB001659, and NIH-R01-GM104987 from the National Institutes of Health. The MIT Laboratory for Computational Physiology received funding from Philips Healthcare to undertake work on the database described in this paper. O.B. is an employee of Philips Healthcare.

**Publication, author and funding related to the eGFR GWAS:**

Liu H, Doke T, Guo D, Sheng X, Ma Z, Park J, Vy HMT, Nadkarni GN, Abedini A, Miao Z, Palmer M, Voight BF, Li H, Brown CD, Ritchie MD, Shu Y, Susztak K. Epigenomic and transcriptomic analyses define core cell types, genes and targetable mechanisms for kidney disease. *Nat Genet*. 2022 Jul;54(7):950-962. doi: 10.1038/s41588-022-01097-w.

This work was supported by the Molecular Pathology and Imaging Core (grant no. P30-DK050306 to K.S.) and Diabetes Research Center (grant no. P30-DK19525 to K.S.) at the University of Pennsylvania for their services. The work in K.S.’s laboratory has been supported by the NIH (grant nos. R01DK087635, R01DK076077 and R01DK105821 to K.S.).

**Publication, author and funding related to the CKD GWAS:**

Wuttke M, Li Y, Li M, Sieber KB, Feitosa MF, Gorski M, Tin A, Wang L, Chu AY, Hoppmann A, Kirsten H, Giri A, Chai JF, Sveinbjornsson G, Tayo BO, Nutile T, Fuchsberger C, Marten J, Cocca M, Ghasemi S, Xu Y, Horn K, Noce D, van der Most PJ, Sedaghat S, Yu Z, Akiyama M, Afaq S, Ahluwalia TS, Almgren P, Amin N, Ärnlöv J, Bakker SJL, Bansal N, Baptista D, Bergmann S, Biggs ML, Biino G, Boehnke M, Boerwinkle E, Boissel M, Bottinger EP, Boutin TS, Brenner H, Brumat M, Burkhardt R, Butterworth AS, Campana E, Campbell A, Campbell H, Canouil M, Carroll RJ, Catamo E, Chambers JC, Chee ML, Chee ML, Chen X, Cheng CY, Cheng Y, Christensen K, Cifkova R, Ciullo M, Concas MP, Cook JP, Coresh J, Corre T, Sala CF, Cusi D, Danesh J, Daw EW, de Borst MH, De Grandi A, de Mutsert R, de Vries APJ, Degenhardt F, Delgado G, Demirkan A, Di Angelantonio E, Dittrich K, Divers J, Dorajoo R, Eckardt KU, Ehret G, Elliott P, Endlich K, Evans MK, Felix JF, Foo VHX, Franco OH, Franke A, Freedman BI, Freitag-Wolf S, Friedlander Y, Froguel P, Gansevoort RT, Gao H, Gasparini P, Gaziano JM, Giedraitis V, Gieger C, Girotto G, Giulianini F, Gögele M, Gordon SD, Gudbjartsson DF, Gudnason V, Haller T, Hamet P, Harris TB, Hartman CA, Hayward C, Hellwege JN, Heng CK, Hicks AA, Hofer E, Huang W, Hutri-Kähönen N, Hwang SJ, Ikram MA, Indridason OS, Ingelsson E, Ising M, Jaddoe VWV, Jakobsdottir J, Jonas JB, Joshi PK, Josyula NS, Jung B, Kähönen M, Kamatani Y, Kammerer CM, Kanai M, Kastarinen M, Kerr SM, Khor CC, Kiess W, Kleber ME, Koenig W, Kooner JS, Körner A, Kovacs P, Kraja AT, Krajcoviechova A, Kramer H, Krämer BK, Kronenberg F, Kubo M, Kühnel B, Kuokkanen M, Kuusisto J, La Bianca M, Laakso M, Lange LA, Langefeld CD, Lee JJ, Lehne B, Lehtimäki T, Lieb W; Lifelines Cohort Study, Lim SC, Lind L, Lindgren CM, Liu J, Liu J, Loeffler M, Loos RJF, Lucae S, Lukas MA, Lyytikäinen LP, Mägi R, Magnusson PKE, Mahajan A, Martin NG, Martins J, März W, Mascalzoni D, Matsuda K, Meisinger C, Meitinger T, Melander O, Metspalu A, Mikaelsdottir EK, Milaneschi Y, Miliku K, Mishra PP; V. A. Million Veteran Program, Mohlke KL, Mononen N, Montgomery GW, Mook-Kanamori DO, Mychaleckyj JC, Nadkarni GN, Nalls MA, Nauck M, Nikus K, Ning B, Nolte IM, Noordam R, O'Connell J, O'Donoghue ML, Olafsson I, Oldehinkel AJ, Orho-Melander M, Ouwehand WH, Padmanabhan S, Palmer ND, Palsson R, Penninx BWJH, Perls T, Perola M, Pirastu M, Pirastu N, Pistis G, Podgornaia AI, Polasek O, Ponte B, Porteous DJ, Poulain T, Pramstaller PP, Preuss MH, Prins BP, Province MA, Rabelink TJ, Raffield LM, Raitakari OT, Reilly DF, Rettig R, Rheinberger M, Rice KM, Ridker PM, Rivadeneira F, Rizzi F, Roberts DJ, Robino A, Rossing P, Rudan I, Rueedi R, Ruggiero D, Ryan KA, Saba Y, Sabanayagam C, Salomaa V, Salvi E, Saum KU, Schmidt H, Schmidt R, Schöttker B, Schulz CA, Schupf N, Shaffer CM, Shi Y, Smith AV, Smith BH, Soranzo N, Spracklen CN, Strauch K, Stringham HM, Stumvoll M, Svensson PO, Szymczak S, Tai ES, Tajuddin SM, Tan NYQ, Taylor KD, Teren A, Tham YC, Thiery J, Thio CHL, Thomsen H, Thorleifsson G, Toniolo D, Tönjes A, Tremblay J, Tzoulaki I, Uitterlinden AG, Vaccargiu S, van Dam RM, van der Harst P, van Duijn CM, Velez Edward DR, Verweij N, Vogelezang S, Völker U, Vollenweider P, Waeber G, Waldenberger M, Wallentin L, Wang YX, Wang C, Waterworth DM, Bin Wei W, White H, Whitfield JB, Wild SH, Wilson JF, Wojczynski MK, Wong C, Wong TY, Xu L, Yang Q, Yasuda M, Yerges-Armstrong LM, Zhang W, Zonderman AB, Rotter JI, Bochud M, Psaty BM, Vitart V, Wilson JG, Dehghan A, Parsa A, Chasman DI, Ho K, Morris AP, Devuyst O, Akilesh S, Pendergrass SA, Sim X, Böger CA, Okada Y, Edwards TL, Snieder H, Stefansson K, Hung AM, Heid IM, Scholz M, Teumer A, Köttgen A, Pattaro C. A catalog of genetic loci associated with kidney function from analyses of a million individuals. *Nat Genet*. 2019 Jun;51(6):957-972. doi: 10.1038/s41588-019-0407-x.

This work received funding from sources specified at https://static-content.springer.com/esm/art%3A10.1038%2Fs41588-019-0407-x/MediaObjects/41588_2019_407_MOESM1_ESM.pdf (Page 23 to 32)

**Publication, author and funding related to the atrial fibrillation GWAS:**

Nielsen JB, Thorolfsdottir RB, Fritsche LG, Zhou W, Skov MW, Graham SE, Herron TJ, McCarthy S, Schmidt EM, Sveinbjornsson G, Surakka I, Mathis MR, Yamazaki M, Crawford RD, Gabrielsen ME, Skogholt AH, Holmen OL, Lin M, Wolford BN, Dey R, Dalen H, Sulem P, Chung JH, Backman JD, Arnar DO, Thorsteinsdottir U, Baras A, O'Dushlaine C, Holst AG, Wen X, Hornsby W, Dewey FE, Boehnke M, Kheterpal S, Mukherjee B, Lee S, Kang HM, Holm H, Kitzman J, Shavit JA, Jalife J, Brummett CM, Teslovich TM, Carey DJ, Gudbjartsson DF, Stefansson K, Abecasis GR, Hveem K, Willer CJ. Biobank-driven genomic discovery yields new insight into atrial fibrillation biology. *Nat Genet*. 2018 Sep;50(9):1234-1239. doi: 10.1038/s41588-018-0171-3.

The Nord-Trøndelag Health Study (the HUNT Study) is a collaboration between the HUNT Research Centre (Faculty of Medicine, Norwegian University of Science and Technology (NTNU)), Nord-Trøndelag County Council, the Central Norway Health Authority, and the Norwegian Institute of Public Health. The K.G. Jebesen Center for Genetic Epidemiology is financed by Stiftelsen Kristian Gerhard Jebsen, the Faculty of Medicine and Health Sciences Norwegian University of Science and Technology (NTNU), and the Central Norway Regional Health Authority. This research has been conducted using the UK Biobank Resource under application number 24460. J.B.N. was supported by grants from the Danish Heart Foundation (16-R107-A6779) and the Lundbeck Foundation (R220-2016-1434). T.J.H. was supported by an American Heart Association Scientist Development Grant (0735464Z). J.A.S. was supported by National Institutes of Health grant R01-HL124232. C.J.W. was supported by National Institutes of Health grants R35-HL135824, R01-HL127564, R01-HL117626–02-S1, and R01-HL130705.

**Publication, author and funding related to the coronary artery disease GWAS:**

Nikpay M, Goel A, Won HH, Hall LM, Willenborg C, Kanoni S, Saleheen D, Kyriakou T, Nelson CP, Hopewell JC, Webb TR, Zeng L, Dehghan A, Alver M, Armasu SM, Auro K, Bjonnes A, Chasman DI, Chen S, Ford I, Franceschini N, Gieger C, Grace C, Gustafsson S, Huang J, Hwang SJ, Kim YK, Kleber ME, Lau KW, Lu X, Lu Y, Lyytikäinen LP, Mihailov E, Morrison AC, Pervjakova N, Qu L, Rose LM, Salfati E, Saxena R, Scholz M, Smith AV, Tikkanen E, Uitterlinden A, Yang X, Zhang W, Zhao W, de Andrade M, de Vries PS, van Zuydam NR, Anand SS, Bertram L, Beutner F, Dedoussis G, Frossard P, Gauguier D, Goodall AH, Gottesman O, Haber M, Han BG, Huang J, Jalilzadeh S, Kessler T, König IR, Lannfelt L, Lieb W, Lind L, Lindgren CM, Lokki ML, Magnusson PK, Mallick NH, Mehra N, Meitinger T, Memon FU, Morris AP, Nieminen MS, Pedersen NL, Peters A, Rallidis LS, Rasheed A, Samuel M, Shah SH, Sinisalo J, Stirrups KE, Trompet S, Wang L, Zaman KS, Ardissino D, Boerwinkle E, Borecki IB, Bottinger EP, Buring JE, Chambers JC, Collins R, Cupples LA, Danesh J, Demuth I, Elosua R, Epstein SE, Esko T, Feitosa MF, Franco OH, Franzosi MG, Granger CB, Gu D, Gudnason V, Hall AS, Hamsten A, Harris TB, Hazen SL, Hengstenberg C, Hofman A, Ingelsson E, Iribarren C, Jukema JW, Karhunen PJ, Kim BJ, Kooner JS, Kullo IJ, Lehtimäki T, Loos RJF, Melander O, Metspalu A, März W, Palmer CN, Perola M, Quertermous T, Rader DJ, Ridker PM, Ripatti S, Roberts R, Salomaa V, Sanghera DK, Schwartz SM, Seedorf U, Stewart AF, Stott DJ, Thiery J, Zalloua PA, O'Donnell CJ, Reilly MP, Assimes TL, Thompson JR, Erdmann J, Clarke R, Watkins H, Kathiresan S, McPherson R, Deloukas P, Schunkert H, Samani NJ, Farrall M. A comprehensive 1,000 Genomes-based genome-wide association meta-analysis of coronary artery disease. *Nat Genet*. 2015 Oct;47(10):1121-1130. doi: 10.1038/ng.3396.

This work received funding from sources specified at https://static-content.springer.com/esm/art%3A10.1038%2Fng.3396/MediaObjects/41588_2015_BFng3396_MOESM57_ESM.pdf (Page 22 to 29)

**Publication, author and funding related to the heart failure GWAS:**

Shah S, Henry A, Roselli C, Lin H, Sveinbjörnsson G, Fatemifar G, Hedman ÅK, Wilk JB, Morley MP, Chaffin MD, Helgadottir A, Verweij N, Dehghan A, Almgren P, Andersson C, Aragam KG, Ärnlöv J, Backman JD, Biggs ML, Bloom HL, Brandimarto J, Brown MR, Buckbinder L, Carey DJ, Chasman DI, Chen X, Chen X, Chung J, Chutkow W, Cook JP, Delgado GE, Denaxas S, Doney AS, Dörr M, Dudley SC, Dunn ME, Engström G, Esko T, Felix SB, Finan C, Ford I, Ghanbari M, Ghasemi S, Giedraitis V, Giulianini F, Gottdiener JS, Gross S, Guðbjartsson DF, Gutmann R, Haggerty CM, van der Harst P, Hyde CL, Ingelsson E, Jukema JW, Kavousi M, Khaw KT, Kleber ME, Køber L, Koekemoer A, Langenberg C, Lind L, Lindgren CM, London B, Lotta LA, Lovering RC, Luan J, Magnusson P, Mahajan A, Margulies KB, März W, Melander O, Mordi IR, Morgan T, Morris AD, Morris AP, Morrison AC, Nagle MW, Nelson CP, Niessner A, Niiranen T, O'Donoghue ML, Owens AT, Palmer CNA, Parry HM, Perola M, Portilla-Fernandez E, Psaty BM; Regeneron Genetics Center, Rice KM, Ridker PM, Romaine SPR, Rotter JI, Salo P, Salomaa V, van Setten J, Shalaby AA, Smelser DT, Smith NL, Stender S, Stott DJ, Svensson P, Tammesoo ML, Taylor KD, Teder-Laving M, Teumer A, Thorgeirsson G, Thorsteinsdottir U, Torp-Pedersen C, Trompet S, Tyl B, Uitterlinden AG, Veluchamy A, Völker U, Voors AA, Wang X, Wareham NJ, Waterworth D, Weeke PE, Weiss R, Wiggins KL, Xing H, Yerges-Armstrong LM, Yu B, Zannad F, Zhao JH, Hemingway H, Samani NJ, McMurray JJV, Yang J, Visscher PM, Newton-Cheh C, Malarstig A, Holm H, Lubitz SA, Sattar N, Holmes MV, Cappola TP, Asselbergs FW, Hingorani AD, Kuchenbaecker K, Ellinor PT, Lang CC, Stefansson K, Smith JG, Vasan RS, Swerdlow DI, Lumbers RT. Genome-wide association and Mendelian randomisation analysis provide insights into the pathogenesis of heart failure. *Nat Commun*. 2020 Jan 9;11(1):163. doi: 10.1038/s41467-019-13690-5.

This project received funding from sources specified at https://static-content.springer.com/esm/art%3A10.1038%2Fs41467-019-13690-5/MediaObjects/41467_2019_13690_MOESM1_ESM.pdf (Page 39 to 46)

**Publication, author and funding related to the stroke and ischemic stroke GWAS:**

Malik R, Chauhan G, Traylor M, Sargurupremraj M, Okada Y, Mishra A, Rutten-Jacobs L, Giese AK, van der Laan SW, Gretarsdottir S, Anderson CD, Chong M, Adams HHH, Ago T, Almgren P, Amouyel P, Ay H, Bartz TM, Benavente OR, Bevan S, Boncoraglio GB, Brown RD Jr, Butterworth AS, Carrera C, Carty CL, Chasman DI, Chen WM, Cole JW, Correa A, Cotlarciuc I, Cruchaga C, Danesh J, de Bakker PIW, DeStefano AL, den Hoed M, Duan Q, Engelter ST, Falcone GJ, Gottesman RF, Grewal RP, Gudnason V, Gustafsson S, Haessler J, Harris TB, Hassan A, Havulinna AS, Heckbert SR, Holliday EG, Howard G, Hsu FC, Hyacinth HI, Ikram MA, Ingelsson E, Irvin MR, Jian X, Jiménez-Conde J, Johnson JA, Jukema JW, Kanai M, Keene KL, Kissela BM, Kleindorfer DO, Kooperberg C, Kubo M, Lange LA, Langefeld CD, Langenberg C, Launer LJ, Lee JM, Lemmens R, Leys D, Lewis CM, Lin WY, Lindgren AG, Lorentzen E, Magnusson PK, Maguire J, Manichaikul A, McArdle PF, Meschia JF, Mitchell BD, Mosley TH, Nalls MA, Ninomiya T, O'Donnell MJ, Psaty BM, Pulit SL, Rannikmäe K, Reiner AP, Rexrode KM, Rice K, Rich SS, Ridker PM, Rost NS, Rothwell PM, Rotter JI, Rundek T, Sacco RL, Sakaue S, Sale MM, Salomaa V, Sapkota BR, Schmidt R, Schmidt CO, Schminke U, Sharma P, Slowik A, Sudlow CLM, Tanislav C, Tatlisumak T, Taylor KD, Thijs VNS, Thorleifsson G, Thorsteinsdottir U, Tiedt S, Trompet S, Tzourio C, van Duijn CM, Walters M, Wareham NJ, Wassertheil-Smoller S, Wilson JG, Wiggins KL, Yang Q, Yusuf S; AFGen Consortium; Cohorts for Heart and Aging Research in Genomic Epidemiology (CHARGE) Consortium; International Genomics of Blood Pressure (iGEN-BP) Consortium; INVENT Consortium; STARNET, Bis JC, Pastinen T, Ruusalepp A, Schadt EE, Koplev S, Björkegren JLM, Codoni V, Civelek M, Smith NL, Trégouët DA, Christophersen IE, Roselli C, Lubitz SA, Ellinor PT, Tai ES, Kooner JS, Kato N, He J, van der Harst P, Elliott P, Chambers JC, Takeuchi F, Johnson AD; BioBank Japan Cooperative Hospital Group; COMPASS Consortium; EPIC-CVD Consortium; EPIC-InterAct Consortium; International Stroke Genetics Consortium (ISGC); METASTROKE Consortium; Neurology Working Group of the CHARGE Consortium; NINDS Stroke Genetics Network (SiGN); UK Young Lacunar DNA Study; MEGASTROKE Consortium, Sanghera DK, Melander O, Jern C, Strbian D, Fernandez-Cadenas I, Longstreth WT Jr, Rolfs A, Hata J, Woo D, Rosand J, Pare G, Hopewell JC, Saleheen D, Stefansson K, Worrall BB, Kittner SJ, Seshadri S, Fornage M, Markus HS, Howson JMM, Kamatani Y, Debette S, Dichgans M. Multiancestry genome-wide association study of 520,000 subjects identifies 32 loci associated with stroke and stroke subtypes. *Nat Genet*. 2018 Apr;50(4):524-537. doi: 10.1038/s41588-018-0058-3.

This project received funding from sources specified at http://www.megastroke.org/acknowledgments.html
